# Polynomial, piecewise-Linear, Step (PLS): A Simple, Scalable, and Efficient Framework for Modeling Neurons

**DOI:** 10.3389/fninf.2021.642933

**Published:** 2021-05-06

**Authors:** Ruben A. Tikidji-Hamburyan, Matthew T. Colonnese

**Affiliations:** School of Medicine and Health Sciences, George Washington University, Washington, DC, United States

**Keywords:** neurons, biophysical models, neurodynamics, CPU, GPU, mobile devices, phenomenological models

## Abstract

Biological neurons can be modeled with different levels of biophysical/biochemical details. The accuracy with which a model reflects the actual physiological processes and ultimately the information function of a neuron, can range from very detailed to a schematic phenomenological representation. This range exists due to the common problem: one needs to find an optimal trade-off between the level of details needed to capture the necessary information processing in a neuron and the computational load needed to compute 1 s of model time. An increase in modeled network size or model-time, for which the solution should be obtained, makes this trade-off pivotal in model development. Numerical simulations become incredibly challenging when an extensive network with a detailed representation of each neuron needs to be modeled over a long time interval to study slow evolving processes, e.g., development of the thalamocortical circuits. Here we suggest a simple, powerful and flexible approach in which we approximate the right-hand sides of differential equations by combinations of functions from three families: Polynomial, piecewise-Linear, Step (PLS). To obtain a single coherent framework, we provide four core principles in which PLS functions should be combined. We show the rationale behind each of the core principles. Two examples illustrate how to build a conductance-based or phenomenological model using the PLS-framework. We use the first example as a benchmark on three different computational platforms: CPU, GPU, and mobile system-on-chip devices. We show that the PLS-framework speeds up computations without increasing the memory footprint and maintains high model fidelity comparable to the fully-computed model or with lookup-table approximation. We are convinced that the full range of neuron models: from biophysical to phenomenological and even to abstract models, may benefit from using the PLS-framework.

## 1. Introduction

Biological neurons are complex computational devices. They combine spatial and temporal input integration with non-linear membrane electrical properties subject to modulation on timescales from milliseconds to days. Almost a century of effort has resulted in detailed knowledge of these processes and equipped us with a mathematical toolbox for their numerical modeling. Current state-of-art multicompartment and single-compartment, conductance-based models (which we will call biophysical models) allow for accurate modeling of a single neuron behavior. However, biophysical models require many parameters that are often non-existent or not feasible to measure, requiring computationally expensive optimization procedures to fit the free parameters. Furthermore, because these models demand extensive computational resources, simulations of even small networks utilizing the biophysical accuracy of cellular dynamics requires supercomputer power.

The burden of computational load becomes a critical factor when the studied biological phenomena evolve on a scale of hours or even days. Computing a large-scale biophysical model over such timescales is not feasible even for modern supercomputers. For example the early stages of thalamocortical and subcortical network development takes more than 2 weeks. During this period, sensory organs generate spontaneous activity in various forms, and this activity sculpts ascending connections initially established by gradients of chemical cues (Cang and Feldheim, 2013). This process of activity-guided synaptic formation and elimination that leads to circuit rearrangements, requires synaptic plasticity that relies on local and whole-cell integration of voltage, ionic conductances and secondary messenger systems that are not easily captured by simple phenomenological models. For example, during the period of initial circuit formation in the visual thalamus and cortex, neurons produce plateau potentials (a long-duration, spikeless depolarization) in response to retinal drive (Colonnese, 2014). Because early synaptic transmission mostly relies upon the slow voltage-sensitive NMDA current, plateau-potentials can potentially change mechanisms for synaptic plasticity similar to those of dendritic plateau-potentials in adult neurons (Bono and Clopath, 2017). Furthermore, in addition to the continual network reconfigurations, in both thalamic and cortical neuron excitability is also maturing, and these processes exist in a feedback loop. For these reasons, modeling the development of brain networks is one of the most challenging simulation problems.

Traditionally the issues of computational load and speed of numerical simulation are resolved using phenomenological models which simulate only the behavior of the cross-membrane potential (usually called voltage *v*) without the details of underlying biophysical/biochemical processes. They are very popular because of the simplicity, small memory footprint, and minimal number of mathematical operations per millisecond of model time. Most critically, they usually come with parameter sets for some neuron intrinsic dynamics (cell-types, such as regular firing, intrinsic bursting, chattering, fast firing, subthreshold resonance, etc.). Phenomenological models allow for large network simulations to be carried out on ordinary desktop computers, graphic accelerators, and mobile system-on-chip (SoC) devices. There are several popular phenomenological models, including: Izhikevich's model (Izhikevich, 2003, 2004), exponential leaky integrate-and-fire model (Brette and Gerstner, 2005; Fontaine et al., 2014; Brette, 2015), generalized linear integrate-and-fire model (Mihalaş and Niebur, 2009), and generalized leaky integrate-and-fire model (Teeter et al., 2018) and many more.

While undeniably useful for many network simulations, modeling of network and cellular dynamics during development and plasticity requires more biophysical detail than current phenomenological models can provide. However, it is not possible to carry out simulations using fully biophysically realistic models as computation of a few minutes of development can take hours or even days of calculations on High-Performance Computing platforms (HPC). Rather, it needs a modeling framework with an optimal balance between (1) model fidelity, i.e., representation of biophysical details, (2) simplicity of mathematical foundations i.e the minimization of the amount of computer power needed for computing 1 s of model time, and (3) flexibility for reconfiguring the modeling network and neuron excitability during a simulation. In addition to modeling evolving circuits, such as during development, improving the biophysical detail of physiological models without a significant increase of computational load may be of use in robotics. Current packages for spiking neurons in robotics include only a limited set of phenomenological models so that package developers can only focus on the code optimization and efficiency for **real-time** performance [for example, CARLsim (Beyeler et al., 2015)].

Here we consider a framework for developing models that can satisfy some of the problems outlined above. It consists of three general and well-known families of functions: Polynomial, piecewise-Linear, and Step (PLS). The components themselves are not novel and have been widely used in many simulations and analyses, implicitly or explicitly for many decades, if not centuries. However, here we show that when combined into one framework based on four core principles, they present a single, well-balanced approach that allows one to simplify and speed up simulations, reduce memory footprint, improve flexibility, scalability, and easily map neuron models onto computational hardware without sacrificing model fidelities. There is no “PLS neuron”; the PLS is a framework, which supports the full range of models from biophysical to phenomenological ones. One can implement an unlimited number of neuron models in the PLS-framework. For example, the FitzHugh-Nagumo model (FitzHugh, 1961) is one of the many possible models in the PLS-framework. Models implemented in PLS-framework can run on the full range of computational devices, including graphical processing units (GPU), central processing units (CPU), microcontrollers, and system-on-chip (SoC) mobile hardware. Moreover, the representation of a neuron in the PLS-framework can help future implementation of this model into dedicated electronic or optical devices.

This paper is organized as follows: In section 2, we go through several kinds of reductions for a biophysical model. This section aims to develop the reader's intuition and provide benchmarks for performance, memory footprint, and fidelity of reduced models compared with the original one. This section is not obligatory and can be skipped. Section 3 defines PLS-framework and gives general guidelines for implementation. In section 4, we consider another example of how to build phenomenological models using the PLS-framework. Section 5 compares two other popular phenomenological neuron models with the PLS-framework in a concrete example of modeling neuron-wise plateau-potentials. Finally, we discuss the advantages and potential drawbacks of the PLS framework.

We stress that **all models we discuss below are examples** used to develop an intuition of how to make a neuron model in the PLS-framework.

## 2. Example: Reduction of Wang and Buzsáki, Single-Compartment, Conductance-Based Model

Here we will consider four different kinds of reduction for a single-compartment model of hippocampal PV+ fast-spiking basket cell, suggested by Wang and Buzsáki (WBM) (Wang and Buzsáki, 1996). This model is one of the most well-studied conductance-based models on the one hand, and it is simple enough for developing intuition on the other. It represents membrane potential (voltage *v*) of the neurons in the standard Hodgkin-Huxley (HH) formalism, where the gating variable for fast sodium activation *m* is instantaneous and substituted by steady-state function [*m*_∞_(*v*)]:

(1) $$\begin{array}{r} c\frac{dv}{dt}=I+g_{l}\left(E_{l}-v\right)+g_{k}n^{4}\left(E_{k}-v\right)+g_{Na}m_{\infty }^{3}\left(v\right)h\left(E_{Na}-v\right) \end{array}$$

where *c* = 1*μ*F is a membrane capacitance, *g*_*l*_ = 0.1, *g*_*Na*_ = 35, *g*_*K*_ = 9 (mS) are conductance for leak, sodium, and potassium ion-channels with reversal potentials *E*_*l*_ = −65, *E*_*Na*_ = 55, and *E*_*K*_ = −90 (mV), correspondingly. *I* is an input current, which may be a constant applied current (*I* = *I*_*app*_) or sum of synaptic currents if the model is embedded into a network.

Two other gating variables are modeled by first-order ordinary differential equations, in which steady-states and time constants depend upon voltage:

(2) $$\begin{array}{l} \begin{array}{c} \frac{dn}{dt}=\phi \left(\alpha_{n}\left(v\right)\left(1-n\right)-\beta_{n}\left(v\right)n\right)=\frac{\phi }{\tau_{n}\left(v\right)}\left[n_{\infty }\left(v\right)-n\right]\\ \frac{dh}{dt}=\phi \left(\alpha_{h}\left(v\right)\left(1-h\right)-\beta_{h}\left(v\right)h\right)=\frac{\phi }{\tau_{h}\left(v\right)}\left[h_{\infty }\left(v\right)-h\right] \end{array} \end{array}$$

where the indices *h* and *n* indicate sodium slow-inactivation and potassium activation gating variables, respectively. *α*_*x*_(*v*) and *β*_*x*_(*v*) are rate functions for an increase or decrease of gating variables (*x* can be *h* or *n*: *x* ∈ {*h n*}). The equations for gating variables can be transformed into a form where time-constants *τ*_*x*_(*v*) = 1/(*α*_*x*_(*v*) + *β*_*x*_(*v*)) and steady-state values *x*_∞_(*v*) = *α*_*x*_(*v*)/*τ*_*x*_(*v*) are explicitly separated (most right, right-hand-side in the Equation 2). The *m*_∞_(*v*) in the Equation (1) is obtained in the same way. Overall the WBM has six rate functions, one *α*(*v*) and one *β*(*v*) function for each *m*, *h*, and *n*. These functions were fitted to represent the biological neuron accurately and, therefore, will be considered here as the target model for different approximations. Each rate function contains one exponential function and needs to be computed at least 100 times per millisecond. We refer to the solution obtained with computing all the required exponential functions as the original or “fully computed” model. Computation of 600 exponential functions for each millisecond of model time is not an enormous computational load and can be done on modern desktop computers in real-time. However, as soon as the network size increases, the computations needed to compute a millisecond of model time increase proportionally. As a result, modeling 1 s of dynamics of the rat CA1 hippocampal inhibitory network, which consists of ~6,000 interneurons, requires HPC (Tikidji-Hamburyan et al., 2019).

### 2.1. Lookup-Table Approximation

The lookup-table approximation is a classical method for computation acceleration in a numerical problem of these kinds, known for centuries. It is extensively used in such software as NEURON and GENESIS (Hines, 1984; Tikidji-Hamburyan et al., 2017). This approximation is based on a straightforward algorithm: First, before simulation, one needs to pre-compute lookup tables for values of *m*_∞_(*v*), *h*_∞_(*v*), *n*_∞_(*v*), *τ*_*h*_(*v*), and *τ*_*n*_(*v*) in a full range of voltages. Usually, this range goes between the lowest possible to the highest possible voltages. The range is divided into intervals with constant steps. For example, this may be a range from −100 to 60 mV with 1 mV step. With pre-computed tables, one solves differential equations (1, 2) using linear interpolation between table rows instead of computing exponential functions. The voltage at a current time moment of a numerical solution is used to find indices of two rows in the lookup table closest to the membrane voltage. Using these two indices, one can query values for all steady-states and time constants of gating variables and linearly interpolate between these values (see Supplementary Figure 1 and pseudo code below). The table size defines the deviation of a result obtained with this approximation from the fully computed solution. Due to the digital nature of modern computers, this approximation error is approaching zero when the lookup table's step approaches the double float point precision; therefore, the accuracy of a solution is limited only by lookup-table size and the available memory. The usual rule of thumb is to compute the lookup-table with 1 mV. This step size gives <5% error for the classical HH model, while the 0.1 mV step decreases the approximation error to <3% (Tikidji-Hamburyan et al., 2017).

One of the critical features of the lookup-table approximation is that all computations can be simply mapped on the hardware. Indeed, linear interpolation can be performed in the constant and fixed numbers of operations, known in advance. In other words, the number of elementary operations is invariant to the dynamical system state and the accuracy of the approximation. The table has a constant voltage offset between rows; therefore, row indexing is a matter of subtraction, division, and casting a result to an integer number. The pseudo-code is shown in Listing 2.1.

**Listing 2.1:**
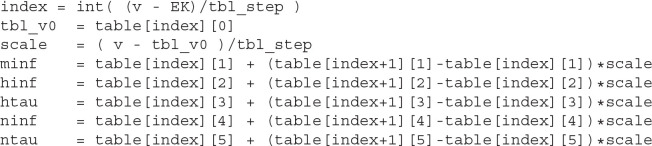
Pseudo-code of lookup table implementation.

**Figure 1:**
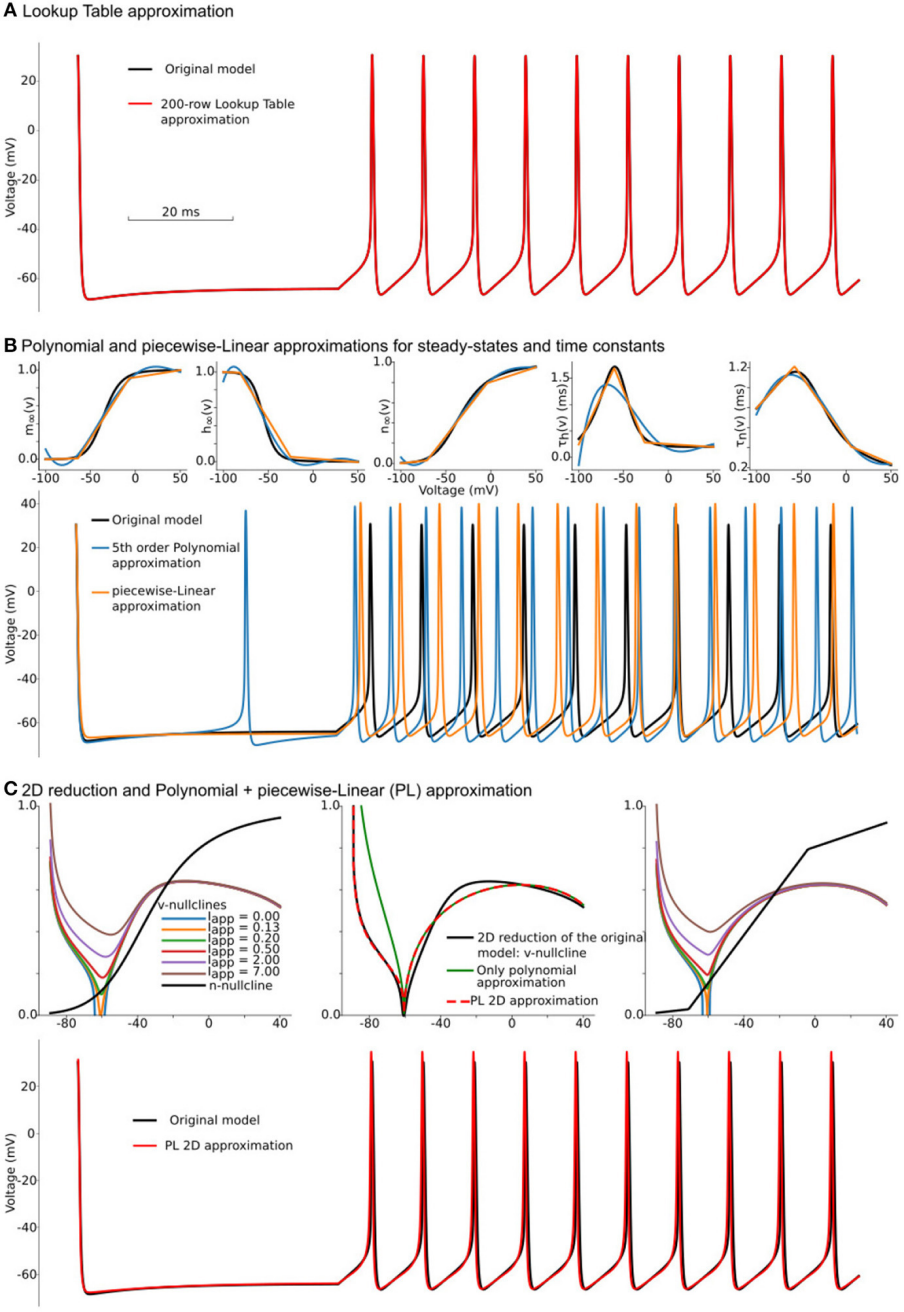
Constructing approximations of Wang and Buzsáki biophysical model. **(A)** Voltage traces for the original model (black line) overlapped with solution based upon lookup-table approximation (red line). **(B)** *Top from left to right*: Steady-state for fast sodium activation (*m*_∞_(*v*)), inactivation (*h*_∞_(*v*)), and delay-rectifier potassium activation (*n*_∞_(*v*)) and time constants for sodium inactivation (*τ*_*h*_(*v*)) and potassium activation [*τ*_*n*_(*v*)] for the original model and two approximations: black lines—original model, blue lines—5^th^ order polynomial interpolation, orange lines—piecewise-Linear approximation. *Bottom*: Voltage traces for the same approximations. **(C)** *Top left*: voltage nullclines at different applied currents (*I* = *I*_*app*_) for the original model reduced to 2 dimensions. *Top center*: Construction of only polynomial (green line) or Polynomial+piecewise-Linear approximation (PL 2D, red dashed line) from the original model after the reduction (black line). *Top right*: nullclines for the PL2D model (the same color-code and applied currents as in the left plot) *Bottom*: Voltage traces for original model (black line) and PL2D approximation (red line).

In our example, we use the lookup table from *E*_*K*_ to *E*_*Na*_ with 200 rows (0.725 mV offset per row), which leads to a table size of 3.9 kilobytes. Figure 1A shows voltage traces for the original model's solution (black line) and a solution obtained with a 200-row lookup table (red line). Although, as we will show later, the lookup table approximation is one of the fastest and most accurate methods to simplify computations in right-hand-sides (RHS) of differential equations, it comes with a significant increase of memory footprint. There are two major problems with lookup tables—large memory footprint and unpredictable random access to the table rows. During a simulation, a solver randomly accesses the rows in the table. That is not a sequential reading from the table, and therefore order in which the rows are read is unpredictable. For modern CPUs, this random access requires a memory allocation for the whole table in a relatively small cache memory. Even for our toy-model, the lookup table holds more than 6% of CPU L1 cache. With an increase in the number of ion-channels and the number of segments with heterogeneous channels, the size of the required memory for lookup tables increases rapidly. As a result, the frequent access to the main memory acts as a “bottle-neck” in massive lookup-table based computations. Moreover, suppose the model consists of a heterogeneous population of neurons. In this case, the dynamical property of some of the ion-channels may gradually change within the population; requiring that lookup tables for these channels are pre-computed for **each neuron in the model independently**. This heterogeneity may easily challenge even the size of the main memory in modern computers.

One can try to overcome the first obstacle by reducing the number of rows in the lookup-tables. However, this results in a second problem: due to the requirement of even steps between rows, the table's accuracy deteriorates with a decrease in the number of rows. For a small number of rows, the lookup-table may have several segments where there should only be one long section, and, at the same time, one segment of the table can linearize a part of the function where a few short pieces can dramatically increase accuracy. That means the points where linear interpolation switches to another slope should not be evenly distributed in the range of voltages (see further discussion of lookup-tables in the subsection 6.3). This problem is solved by the piecewise-linear approximation.

### 2.2. Polynomial and piecewise-Linear Approximations

Other approaches widely used in theoretical analyses are to use polynomial (*P*) or piecewise-linear (*L*) approximations for the same functions *m*_∞_(*v*), *h*_∞_(*v*), *n*_∞_(*v*), *τ*_*h*_(*v*), and *τ*_*n*_(*v*). For our example, we will use just a 2 or 3 point L-functions as follows:

(3) $$L_{2}(x,a_{0},x_{0},y_{0},a_{1})=\{\begin{array}{ll} y_{0}+a_{0}(x-x_{0}) & x\leq x_{0}\\ y_{0}+a_{1}(x-x_{0}) & x>x_{0} \end{array}$$

(4) $$L_{3}(x,a_{0},x_{0},y_{0},x_{1},y_{1},a_{2})=\{\begin{array}{ll} y_{0}+a_{0}(x-x_{0}) & x\leq x_{0}\\ y_{0}+a_{1}(x-x_{0}) & x_{0}<x\leq x_{1}\\ y_{1}+a_{2}(x-x_{1}) & x>x_{1} \end{array}$$

where $a_{1}=\frac{y_{1}-y_{0}}{x_{1}-x_{0}}$ and $a_{2}=\frac{y_{2}-y_{1}}{x_{2}-x_{1}}$ should be pre-computed before simulations. Polynomial (*P*) functions can also approximate the same functions for voltage-dependencies of steady-states and taus. Taking into account that the number of operations in the *L*_3_ function is equivalent to the number of operations in the 5^*th*^ order polynomial series, we compare these approximations against each other. Although *L* approximation produces slightly better results, both *P* (blue lines) and *L* (orange lines) approximations do not show a good match to the original model (black lines) in Figure 1B. Moreover, due to a bigger error, *P* approximation spikes even at zero current. As discussed in section 6.8, these results can be improved by increasing the polynomial order or number of points in piecewise-linear optimizations. However, both approaches increase the number of computations and the amount of memory needed to hold coefficients. Note that nested (*P*, *L*, and *S*) functions can deliver a much better approximation than was achieved here (see section 6.8 for a brief discussion). For the further increase in speed of the Wang–Buzsáki, we can perform Rinzel's reduction of a 3-dimensional dynamical system (1, 2) into a 2-dimensional one.

### 2.3. 2D Reduction and PL Approximation

As Rinzel pointed out in the seminal paper (Rinzel, 1985), if the dynamics of *h* and *n* gate variables have similar time constants and trajectory in the *h* − *n* plane and can be approximated as a linear function [for example, in the standard Hodgkin-Huxley model (Hodgkin and Huxley, 1952)], the dimensionality of the dynamical system is lower than the full system. The Supplementary Figure 3 shows the WBM trajectory and best linear approximation of it in the *h* − *n* plane, which reaches more than 90% accuracy. Therefore the reduced dynamical system can be derived as follows:

(5) $$\begin{array}{l} c\frac{dv}{dt}=I+g_{l}(E_{l}-v)+g_{k}n^{4}(E_{k}-v)\\ \;+g_{Na}m_{\infty }^{3}(v)(\epsilon +\kappa n)(E_{Na}-v)\\ \;\frac{dn}{dt}=\frac{\phi }{\tau_{n}(v)}\left[n_{\infty }(v)-n\right] \end{array}$$

where *ϵ* and *κ* are coefficients of linear regression. The system (5) can be analyzed using the standard graphical method on the phase-plane. The vector field switches the direction along the lines *dv*/*dt* = 0 and *dn*/*dt* = 0, called *nullclines*. Nullclines for the system (5) are shown in Figure 1C (*top row left*). With an increase of the applied current *I* = *I*_*app*_, the N-shaped voltage nullcline (*dv*/*dt* = 0) is rising (color lines for different *I*_*app*_) and loses two intersections with the n-nullcline (*dn*/*dt* = 0, black line). The intersection points manifest stable and unstable fixed points, which collide and annihilate, and therefore the dynamic system undergoes the saddle-node bifurcation.

To simplify this system, we can approximate nullclines by one of the *P* or *L*-functions above. At *I* = *I*_*app*_ = *I*_0_ ≈ 0.13*μ*A, the *v*-nullcline touches *n* = 0 line at *v*_0_ = 60.6 mV. The second intersection with *n* = 0 appears at sodium reversal potential *v*_1_ = *E*_*Na*_ = 55 mV. Therefore one can try to substitute *v*-nullcline by a 3rd order polynomial $a_{0}{\left(v-v_{0}\right)}^{2}\left(v_{1}-v\right)$ (green curve on Figure 1C *top row center*). Although this approximation is close to the right branch of the voltage nullcline, it cannot approximate the left branch well. To fix this discrepancy, we can divide the polynomial term by *L*_1_ function, with **the point of the slope changing at the same** ***v*_0_** **voltage**, and **therefore the change of slopes in** ***L*_1_** **function cannot change the smoothness of the overall nullcline** (red curve).

That is the critical concept of the PLS-framework, which allows us to have **a smooth and continuous solution** of the dynamical system, even though **the governing system consists of non-smooth or discontinuous functions**. Finally, we can substitute n-nullcline with the same *L*_3_ curve as in the previous L-approximation. Overall, our approximation shows a good match for voltage nullclines at different currents (Figure 1C *top right* and in the Supplementary Figure 4). By substitution of *τ*_*n*_(*v*) to *L*_3_ approximation and by introduction voltage time constant as *L*_1_ function we can construct a reduced PL-approximation of WBM as follows:

(6) $$\begin{array}{l} \begin{array}{c} {\;L}_{1}\left(v,s_{v0},v_{\tau },\tau_{v0},s_{v1}\right)\frac{dv}{dt}=I-I_{0}+a_{0}\frac{{\left(v-v_{0}\right)}^{2}\left(v_{1}-v\right)}{L_{2}\left(v,1,v_{0},1,0\right)}+g_{k}n^{4}\left(E_{k}-v\right)\\ L_{3}\left(v,s_{n_{\tau },0},v_{n_{\tau },0},n_{n_{\tau },0},v_{n_{\tau },1},n_{n_{\tau },1},s_{n_{\tau },2}\right)\frac{dn}{dt}=L_{3}\left(v,s_{n_{\infty },0},v_{n_{\infty },0},n_{n_{\infty },0},v_{n_{\infty },1},n_{n_{\infty },1},s_{n_{\infty },2}\right)-n \end{array} \end{array}$$

Although this system contains a lot of constants, which can be found in Supplementary Code along with full reduction and tests, surprisingly, it provides a better approximation for the original model than P or L separately (Figures 1B,C *bottom*) and close to lookup table approximation (Figure 1A *bottom*). We will refer to this system as the *PL2D reduction*.

### 2.4. Quantitative Assessment of WBM Approximations

**Figure 2:**
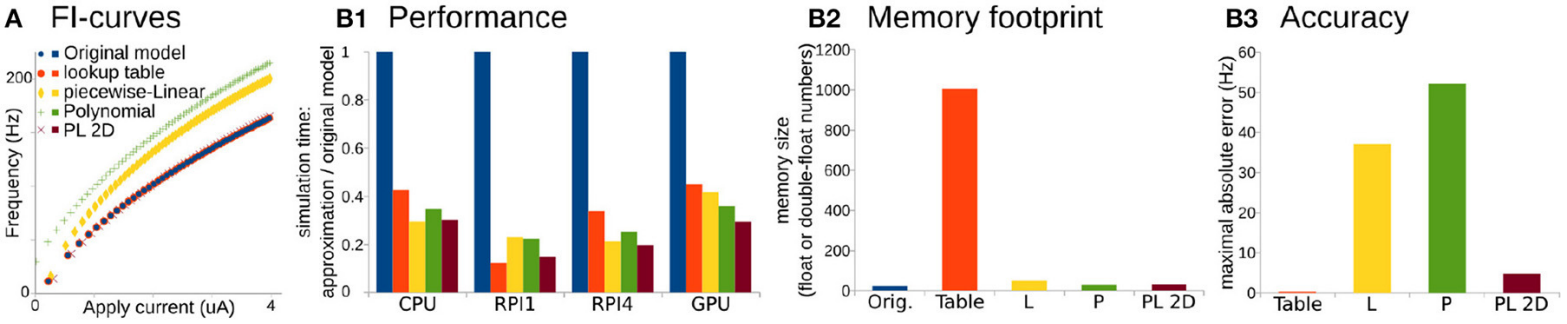
Comparison of original Wang and Buzsáki model with different approximations. **(A)** FI-curves, used for accuracy assessment. **(B1)** Performance Of 3D lookup table (red), 3D piecewise-Linear (L, yellow), 3D Polynomial (P, green), and 2D Polynomial+piecewise-Linear approximations (PL2D, brown) in comparison with the original 3D model (blue) computed on four different platforms: *CPU*—Intel Core i5-5257U, *RPI1*—Raspberry Pi 1, *RPI4*—Raspberry Pi 4, and *GPU*—NVidia GeForce RTX 2080 Ti. **(B2)** Assessment of memory size needed for model constants in the total number of single, double, or long double precision numbers. **(B3)** Standard squared error between FI-curve of the original model and four different approximations.

To quantitatively assess accuracy, performance, and memory footprint of different approximations, we perform two tests: (1) a single run of all approximations a single neuron model for 10 min of model time (60,000,000 iterations of Euler method with constant 0.01 ms time-step) on different computational platforms, and (2) a standard 10 s ramp-protocol to obtain F-I curves (Figure 2A). Note that we use the *odeint* method built into the *scipy* library for the second test because we seek an assessment of the approximation accuracy. However, the Euler method with a constant 0.01 ms step, produces similar results (see Supplementary Code with the Euler method). We did not use these runs for performance benchmarks because we could not control the total number of elementary steps that *odeint* performs due to the adaptive time-step algorithm.

For the performance assessment, all approximations were implemented in C-language. Tests were run on four different platforms: as a single thread process on Intel Core i5-5257U CPU, on Raspberry Pi 1 and 4 ARM CPUs, and as 256 independent threads on NVidia GeForce RTX 2080 Ti GPU. We consider ARM-based single-core CPU in Raspberry Pi 1 and quad-core CPI in Raspberry Pi 4 as representative examples of system-on-chip mobile devices. Unfortunately, we cannot present the same benchmarks for microcontrollers because the lookup-table size exceeds the 2 kb static random-access memory of the Arduino microcontroller available for this project. We assess performance as the minimum wall-clock time required to compute the task normalized by the minimum wall-clock time required to compute the original model (see Materials and Methods for more details).

Overall any approximation is twice as fast compared to the original model (Figure 2B1). Again, PL2D approximation offers the best or the second after best speedup of the performance, potentially because differential equations were reduced to 2D. Although, as expected, the lookup-table approximation has the largest memory footprint (Figure 2B2) and offers the best approximation of the original model (Figure 2B3), it is not always the fastest approximation. An accuracy of just *P* or just *L* approximation is low: the maximum absolute error for *P* exceeds 30% of the model dynamic range, the error for *L* approximation is higher than 20% of the dynamic range, while the PL2D error is <4% of the dynamic range (Figure 2B3). Thus, the *P* or *L* approximations of the original model cannot offer a better approximation than a mix of PL functions, although these approximations model a full 3D dynamical system, not the 2D reduction. Overall, this example shows that **combinations** of *P* and *L*-functions can provide appropriate accuracy of neuron dynamics, significant computational speedup, and lower memory footprint compared to other reductions. With an additional class of Step functions, which appears in phenomenological models, we now can formally define the PLS-framework.

## 3. Definition of the PLS-Framework

A neuron's dynamics are modeled in PLS-framework as

- a system of **continuous (without resetting) dynamical variables,**
- the right-hand sides of differential equations consist of linear combinations of Polynomial, piecewise-Linear, and Step functions;
- specific points of these functions can be aligned to make governing differential equations smooth and continuous if needed;
- specific points of both families of *L* and *S* functions are not bifurcation points or fixed points of the dynamic system themselves, and therefore, right-hand sides of the system are smooth, continuous, and continuously differentiable in a neighborhood of bifurcation/fixed points.

The family of Polynomial functions, used in this framework is better to present as a recursive hierarchy with the first-order linear function *P*_1_(*x, x*_0_) = (*x*_0_ − *x*) as the core function. All higher orders can be defined as follows:

$$\begin{array}{l} P_{2}(x,x_{0},x_{1})\;=P_{1}(x,x_{0})P_{1}(x,x_{1})\\ P_{3}(x,x_{0},x_{1},x_{2})=P_{2}(x,x_{0},x_{1})P_{1}(x,x_{2})\\ \vdots \end{array}$$

As we saw in the example above, it is also useful to define *P*-functions with one root less than the order of the polynomial. For example, a polynomial of 3rd order with two roots (*P*_32_), etc.

$$\begin{array}{l} P_{32}(x,x_{0},x_{1})\;=P_{1}(x,x_{1}){\left[P_{1}(x,x_{0})\right]}^{2}\\ P_{43}(x,x_{0},x_{1},x_{2})=P_{2}(x,x_{1},x_{2}){\left[P_{1}(x,x_{0})\right]}^{2}\\ \vdots \end{array}$$

Similarly, we can define the core function of the piecewise-Linear family as *L*_0_(*x, x*_0_, *y*_0_, *a*_0_) = *y*_0_ + *a*_0_(*x* − *x*_0_). *L*_0_ function is a continuous linear function that represents a linear segment in all other *L* functions. Therefore, the *L*_1_ function can be defined as follows:

$$L_{1}(x,x_{0},y_{0},a_{0},a_{1})=\{\begin{array}{ll} L_{0}(x,x_{0},y_{0},a_{0}) & x\leq x_{0}\\ L_{0}(x,x_{0},y_{0},a_{1}) & x>x_{0} \end{array}$$

Recursively, *L*_2_ can be defined as

$$L_{2}(x,x_{0},y_{0},x_{1},y_{1},a_{0},a_{2})=\{\begin{array}{ll} L_{0}(x,x_{0},y_{0},a_{0}) & x\leq x_{0}\\ L_{1}(x,x_{1},y_{1},\frac{y_{1}-y_{0}}{x_{1}-x_{0}},a_{2}) & x>x_{0} \end{array}$$

and *L*_3_ as

$$\begin{array}{c} L_{3}(x,x_{0},y_{0},x_{1},y_{1},\\ x_{2},y_{2},a_{0},a_{3}) \end{array}=\{\begin{array}{ll} L_{0}(x,x_{0},y_{0},a_{0}) & x\leq x_{0}\\ L_{2}(x,x_{1},y_{1},x_{2},y_{2}\frac{y_{1}-y_{0}}{x_{1}-x_{0}},a_{3}) & x>x_{0} \end{array}$$

and so on.

Finally, the family of Step functions also has a core-function, that is

$$S_{1}(x,x_{0},y_{0},y_{1})=\{\begin{array}{ll} y_{0} & x<x_{0}\\ \frac{y_{1}+y_{0}}{2} & x=x_{0}\\ y_{1} & x>x_{0} \end{array}$$

and therefore all higher-order *S* functions can be defined recursively:

$$\begin{array}{l} S_{2}(x,x_{0},x_{1},y_{0},y_{1},y_{2})\;=S_{1}(x,x_{0},y_{0},S_{1}(x,x_{1},y_{1},y_{2}))\\ S_{3}(x,x_{0},x_{1},y_{0},y_{1},y_{2},y_{3})=S_{1}(x,x_{0},y_{0},S_{2}(x,x_{1},y_{1},y_{2},y_{)})\\ \vdots \end{array}$$

**Figure 3:**
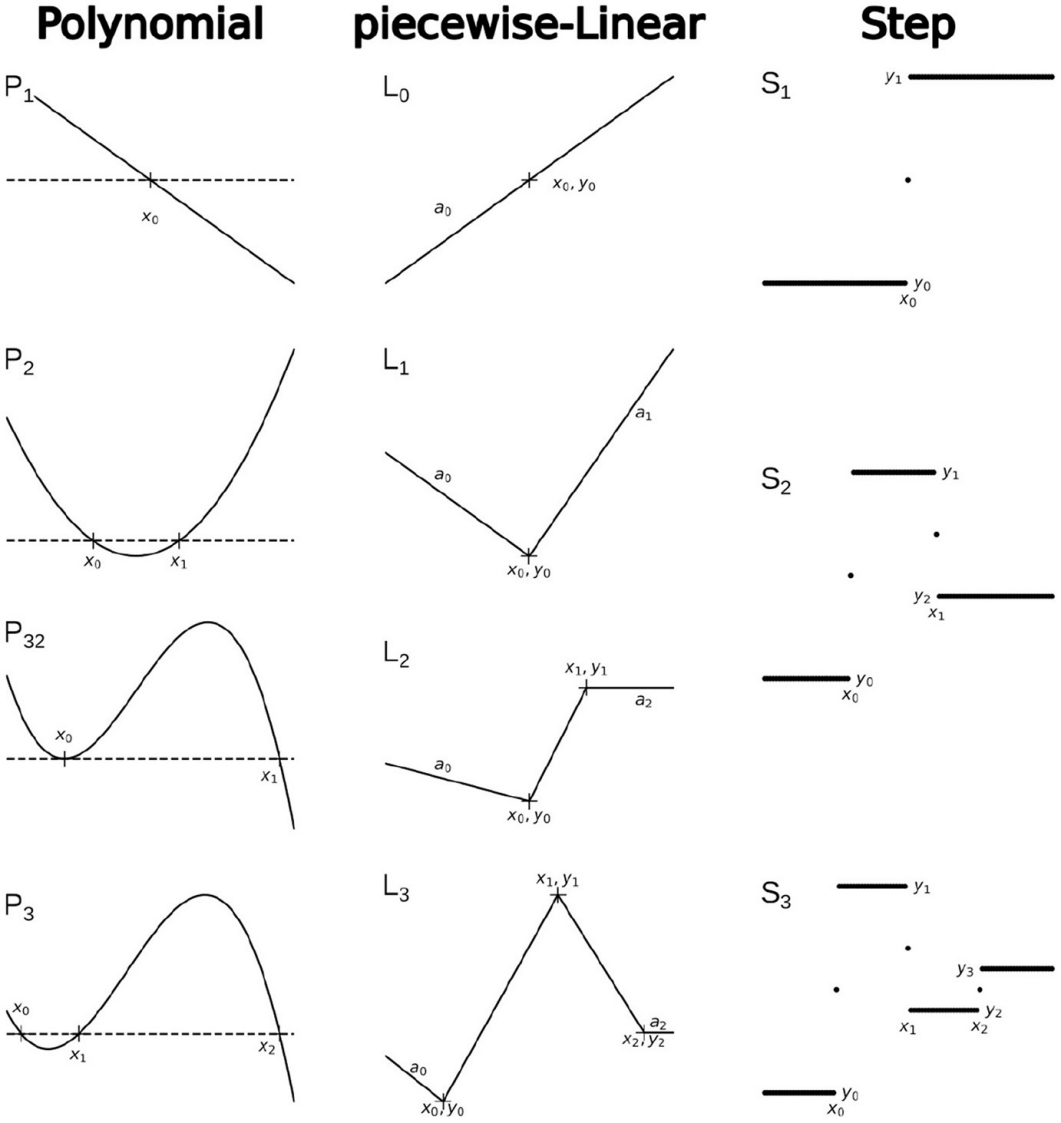
Examples of functions, which comprise the PLS-framework.

Examples of the basic functions and recursive hierarchical functions for each family are shown in Figure 3.

**Figure 4:**
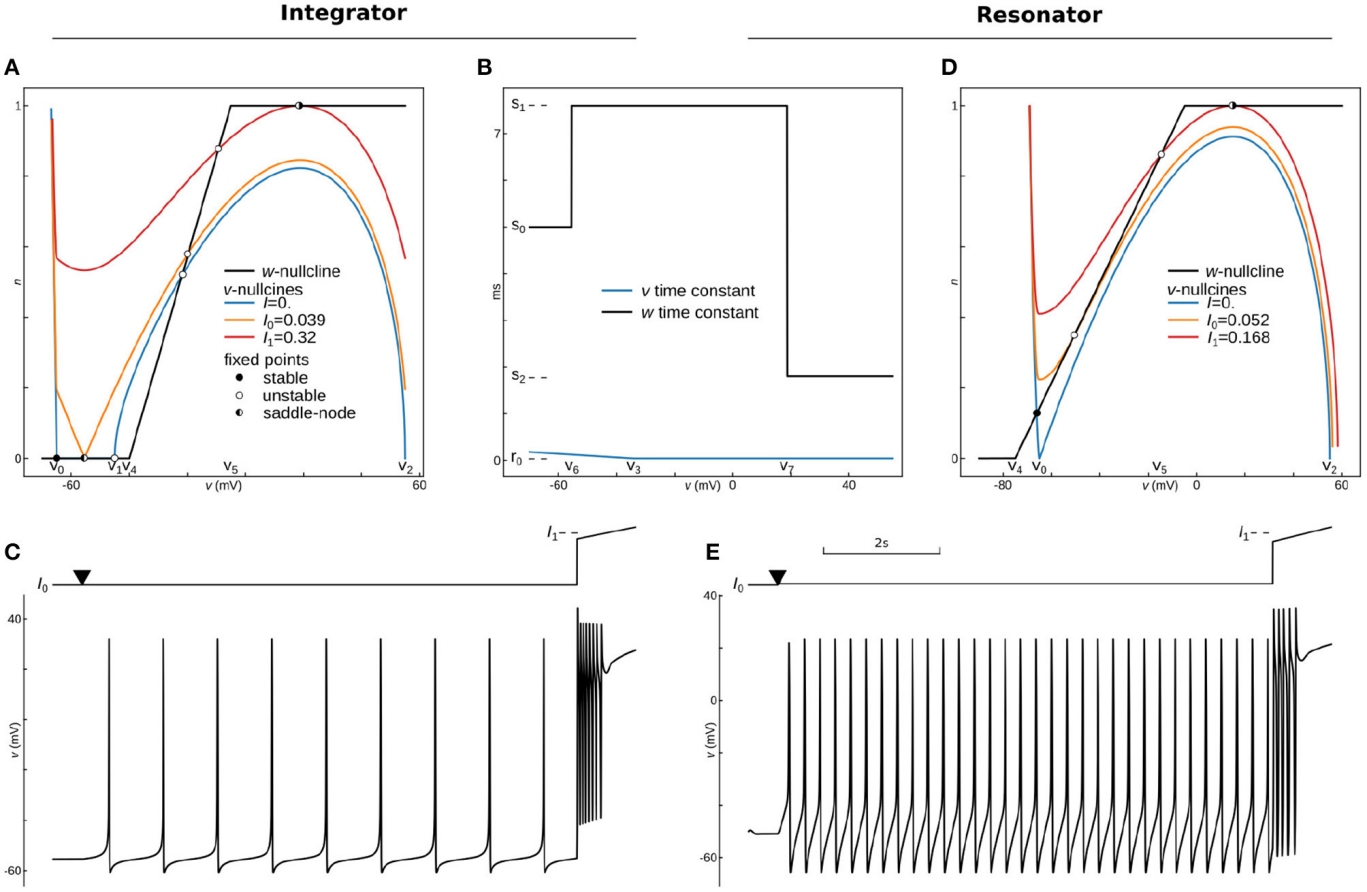
Phase-Plane Analysis and dynamics for phenomenological models of Integrating and Resonant neurons. Phase-portrait **(A)** and time constants **(B)** for the integrator model with parameters: *v*_0_ = −65 mV, *v*_1_ = −45 mV, *v*_2_ = 55 mV, *a*_0_ = 3.5 10^−6^, *a*_1_ = −10^−4^(mV)^−1^, *v*_3_ = −35 mV, *r*_0_ = 0.04 ms, *r*_1_ = −0.004 ms/mV, *v*_4_ = −40 mV, *v*_5_ = −5 mV, *v*_6_ = −55.45 mV, *v*_7_ = 18.78 mV, *s*_0_ = 5 ms, *s*_1_ = 7.6 ms, *s*_2_ = 1.8 ms, *k* = 2. Voltage nullclines are shown for zero current at resting (blue curve), for saddle-node bifurcation at the onset of pacing (*I*_0_, yellow curve), and for saddle-node bifurcation at the onset of depolarization block (*I*_1_, red curve). **(C)** Protocol (*top*) and voltage trace (*bottom*) of integrator dynamics. The model was held almost at the bifurcation point (*I*_0_) for 500 ms, and then the applied current slightly increases at the point marked by triangle for 9 s. At the last second, a ramp of the input current traverses from 0.9*I*_1_ to 1.1*I*_1_ to show the depolarization block. **(D)** Phase-plane analysis for the resonator, with the same as for integrator parameters except for *v*_1_—is not used in this model, and *a*_0_ = 3.25 10^−6^, *v*_4_ = −75 mV, *v*_5_ = −5 mV, *v*_6_ = −55.5 mV, *v*_7_ = 18 mV. Voltage nullclines are shown for zero current at resting (blue curve), for Andronov-Hopf bifurcation (*I*_0_, yellow curve), and for saddle-node bifurcation at the onset of depolarization block (*I*_1_, red curve). **(E)** Dynamics of the resonator. The protocol is the same as for the integrator.

### 3.1. Implementation Note

Although the recursive notation used above is extremely compact and easy to understand, it is not optimal for many programming languages. In C and C++, it is better to define a macro of a core family function and then define macros for each hierarchical function. Here we provide a possible implementation of PLS functions in C (Listing 3.1) and in Python (Listing 3.2).

**Listing 3.1:**
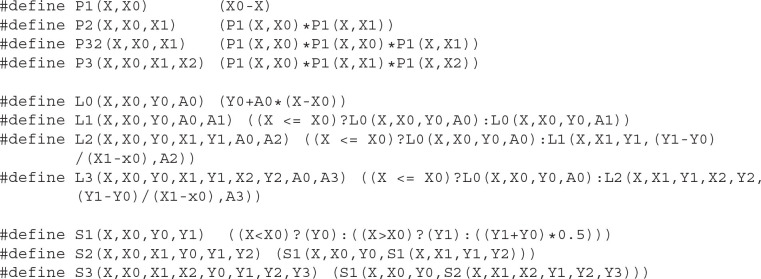
Implementation of PLS functions in C-language.

**Listing 3.2:**
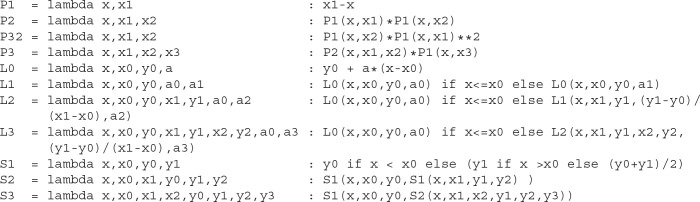
Implementation of PLS functions in Python.

The reader can find C/C++, Python, and XPP (Ermentrout, 2002) implementations as well as a module for Brian-2 simulator (Stimberg et al., 2019) in ModelDB (McDougal et al., 2017) at http://modeldb.yale.edu/266863. Note that C/C++ macros can be used directly in both Cython and NEURON's NMODL extensions (Hines and Carnevale, 2000).

### 3.2. Analytical Note

There is the most common potential mistake during the construction of neural models in the PLS-framework, that is constructing a bifurcation or fixed point as a specific point of *L* or *S* function **only**. One can use *L* or *S* functions to change slopes for *P* functions in RHS, as we did in the example above. In this case, RHS is still continuous and continuously differentiable at the bifurcation point, where the slope of the *P* function is zero, as at any fixed point. Alternatively, it is tempting to substitute a cubical *P*_32_ in our example by 3 or 4 branches *L*_3_ or *L*_4_ functions. However, this is a recipe for a model with **unpredictable behavior**. The roots of such strange and sometimes “paradoxical” behavior is, in fact, that a saddle-node bifurcation appears at the local minimum of *P*_32_, i.e., at the *v*_0_. If we substituted *P*_32_ by any *L*-functions, the bifurcation would happen at the *L* function's specific point, and this implicitly violates the last core principle. As a result, the RHS of differential equations would **not** be continuously differentiable at the saddle-node. This **dynamical system would not have a unique solution** in this case. In practice, this problem may manifest itself as extreme sensitivity to time-step size up-to picoseconds or as an exponential run away to nan when a model should be at resting stable fixed point, or a model can rest at currents way above the threshold. **To avoid such a behavior, any PLS model must be designed to be continuous, smooth, and continuously differentiable at any bifurcation or fixed point**. That is not obvious but critical for obtaining predictable and “well-behaved” models.

## 4. Example: Constructing Phenomenological Models of Integrating and Resonant Neurons

The PLS-framework provides a flexible approach to model neurons, from detailed biophysical to phenomenological models. This section shows how to build phenomenological, simplified models of an integrating neuron (also known as Type 1 or Class 1 neurons) and a resonant neuron (known as Type 2 or Class 2 neurons) in the PLS-framework. We develop here basic models without adaptation or any complex behaviors. However, a curious reader can find examples of linear and non-linear adaptation, as well as some non-traditional/non-biological adaptation models in the Supplementary Code. It should be stressed that all of **these models must be considered as just examples** (“use cases”), not as specific neuron models. In contrast to other phenomenological models, which usually suggest a single dynamical system (i.e., system of differential equations) with a set of parameters for each different neuron dynamics, the PLS-framework offers a set of functions and core principles for model development. A large variety of dynamical systems, dynamics, and models can be implemented in the PLS-framework.

Usage of the PLS-framework can speed up computations ranging from multicompartment models on hardware with limited memory, to point-model with simplified dynamics on GPUs. The goal of the section below is to illustrate the logic behind the development of a phenomenological model.

### 4.1. Integrating Neuron

Integrating neural excitability, also known as an integrator, was first described by Hodgkin as the class 1 excitability of giant squid axons. It can produce and sustain very slow spike rates (Hodgkin, 1948). Such an ability to produce an infinitely slow firing rate is usually linked to a specific dynamic system with a saddle-node bifurcation at the spiking threshold (Rinzel and Ermentrout, 1998; Izhikevich, 2007; Gerstner et al., 2009). The saddle-node bifurcation happens when stable and unstable fixed points collide and annihilate each-other. In a two-dimensional system, fixed points are the intersections of the system nullclines, i.e., lines where the rate of change of one of two dynamic variables is zero. The intersection of two nullclines indicates values of the variables where both rates are zero, and therefore in these particular points, the system does not move. Fixed points can be of two types—stable and unstable. For an unstable fixed point, a small perturbation will lead to run away from the fixed point, while in a stable fixed point, dynamics will return the system to the fixed point. For spiking neurons, such a system comprises an inverted N-shaped voltage nullcline ($dv/dt=\dot{v}=0$, aka *v*-nullcline) and sigmoidal slow variable nullcline.

In Rinzel's reduction, activation of a delayed rectifier potassium current is considered a slow variable, as in section 2 above. However, a different variable *w* is usually assigned for the slow variable in phenomenological models, referred to as an adaptation variable. We will use this notation here and consider *w* slow variable and *w*-nullcline (*dw*/*dt* = ẇ = 0) in the current and next sections.

To construct stable and unstable intersections, one can use cubical polynomial *v*-nullcline with three roots and *L*_3_ instead of a sigmoidal function for the construction of a *w*-nullcline. The *v*-nullcline left and right branches with a negative slope are stable branches, while a middle branch with a positive slope is unstable. However, in the biophysical model the left branch is formed by a hyperbolic curve that runs to infinity when voltage is approaching the potassium reversal potential. Therefore, the left branch on the voltage nullcline is much steeper than the middle or right branches, and a *P*_3_ function alone is not enough for accurate modeling of the spike's downstroke. This part is usually omitted in phenomenological models with resetting. As in the example above, one can make the left branch of the polynomial steeper by multiplying it by an *S* or *L* function, with a specific point at is the most negative root of *P*_3_. We will use the *P*_3_()*L*_1_() combination below to make the slope onset smoother and remove the none-biological artifact(notch) in after-spike hyperpolarization.

As the next step, one needs to choose scales for voltage and slow variables. It is most natural for physicists and mathematicians to normalize both variables so they would have the unit range. However, normalization can make challenging the interpretation of model results for biologists; therefore, the usual approach is to keep the standard biological range in millivolts for voltage variable.

The choice of the voltage scale requires to scale the slow variable in the whole model or on the right-hand side of the voltage equation. In the former approach, the slow variable is not limited. It may have arbitrary values, like in Izhikevich's model. In contrast, the scaling of the slow variable in RHS of the voltage equation requires a scaling factor, which can be seen as a “conductance-like” multiplayer. Such a multiplayer can be hidden by scaling the *P*_3_ function and the time-constant of voltage dynamics. Note that the Brian simulator (Goodman and Brette, 2008, 2009; Stimberg et al., 2014) converts dynamical variables, constants, and parameters into the International System of Units (SI) and balances units automatically. Therefore, if the voltage is set to be in mV or V units, the slow variable should have units of electric current (pA |nA |uA |mA |A) or voltage units (mV |V) if voltage time constant is aggregated into a common denominator and has units of time.

**Figure 5:**
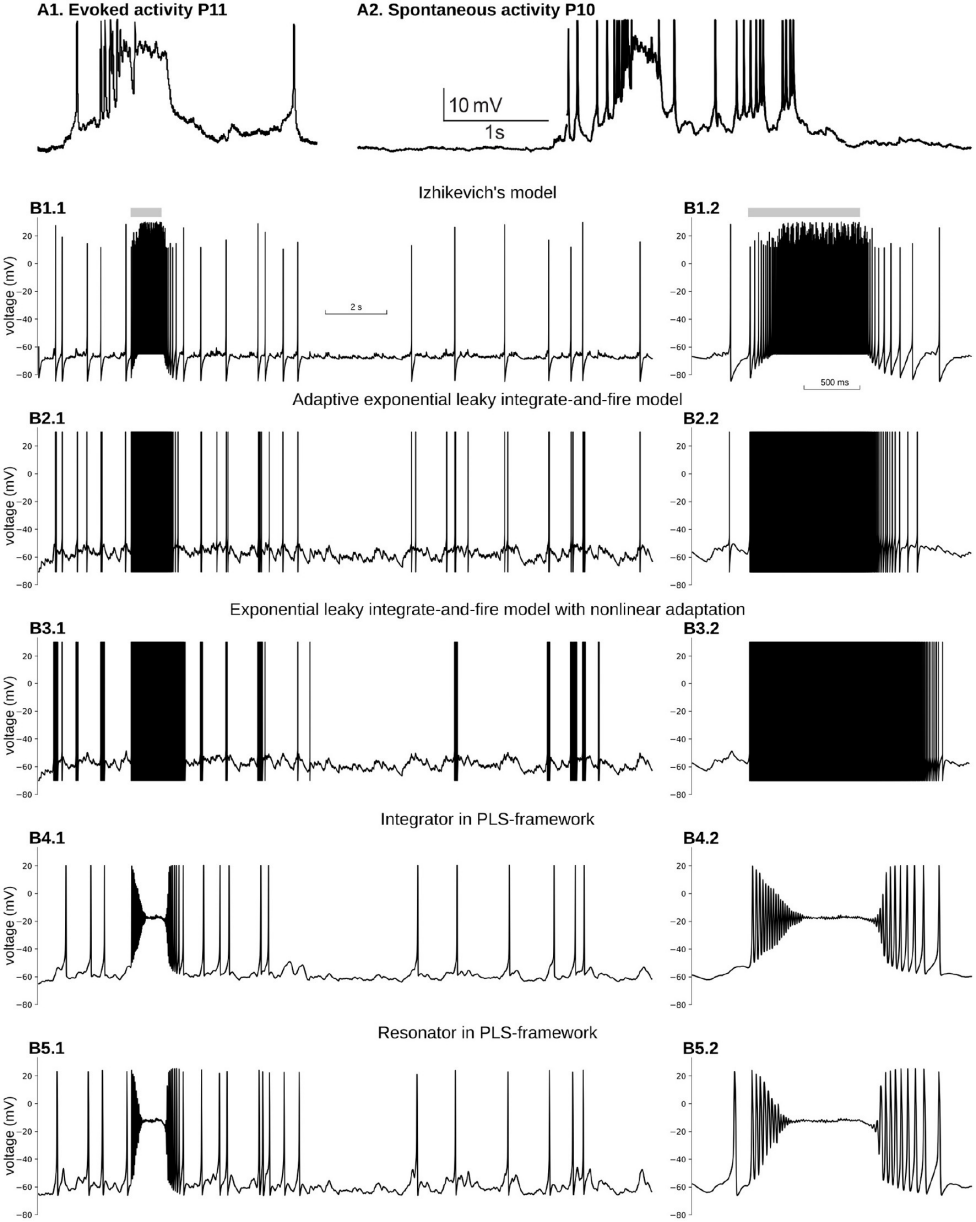
Reproduction of immature, neuron-wide Plateau-Potential in five phenomenological models. **(A1)** Evoked and **(A2)** Spontaneous Plateau-Potentials recorded *in vivo* in rat visual cortex. Graphs reproduced from the data and by the scripts reported previously (Colonnese, 2014). **(B)** Five phenomenological models responses on input similar to dLGN neuron firing during the same period of the development. **(B1)** Izhikevich's model in regular firing mode, **(B2)** Exponential Leaky Integrate-and-Fire model with linear adaptation, **(B3)** Exponential Leaky Integrate-and-Fire model with non-linear adaptation, **(B4)** Type 1 neuron implemented in PLS-network (parameters are the same as in Figures 4A–C), **(B5)** Type 2 neuron implemented in PLS-network (parameters are the same as in Figures 4B,D,F). For each model, subplot **BX.2** is the zoomed burst on subplot **BX.1**. Synaptic conductance was adjusted to obtain a realistic 0.7–1 Hz firing rate outside the burst. For **(B3)**, synaptic conductance was set higher than the adjusted level in an attempt to obtain PP for the stronger synaptic drive.

Saddle-node bifurcation is necessary but not sufficient to produce arbitrary slow oscillations because type 1 excitability needs a trajectory that passes through the half-stable fixed point at the bifurcation (Knowlton et al., 2020). In other words, a limit circle should go through the saddle-node, and therefore this bifurcation is known as a saddle-node on an invariant curve or cycle (SNIC). To guaranty SNIC bifurcation, we can slow down *w* in the range where *v*-nullcline has an unstable middle branch. The simplest way is to introduce *S*_2_ function for *w* time constant and *L*_1_ function for the *v* time constant, which leads us to the simple system of two ordinary differential equations.

(7) $$\begin{array}{l} L_{1}(v,v_{3},r_{0},r_{1},0)\dot{v}=P_{3}(v,v_{0},v_{1},v_{2})L_{1}(v,v_{0},a_{0},a_{1},0)+I-w^{k}\\ S_{2}(v,v_{6},v_{7},s_{0},s_{1},s_{2})\dot{w}=L_{2}(v,v_{4},0,v_{5},1,0,0)-w \end{array}$$

where *I* is an input current, *k* is the power for the slow variable (usually 1, 2, or 4), and the rest are parameters. All parameters are indicated on the phase-plane and time-constant graphs for this dynamic system, shown in Figures 5A,B. At rest (*I* = *I*_*app*_ = 0), the voltage nullcline (blue curve) intersects *w*-nullcline (black curve) at three fixed points. The most left fixed point is stable (filled circle), while the two others are unstable (open circles). An increase of input current moves *v*-nullcline up, and two stable and unstable fixed points shift toward each other. At the applied current *I* = *I*_*app*_ = *I*_0_ ≈ 0.039 (a yellow curve on Figure 5A), these two fixed points collide, creating one half-stable fixed point (half-filled circle). With a further increase in input current, nullclines lose intersection, stable and unstable fixed points annihilate, manifesting saddle-node bifurcation. The rate of change approaches zero as the dynamic variable gets close to its nullcline. Therefore these rates are minimal in the gap between the two nullclines at the bifurcation. Thus, the gap (also called a bottleneck or slow channel) can hold the trajectory arbitrarily long, making possible arbitrary low firing frequency for the integrator. Critically, the further increase in input current brings voltage nullcline closer to the upper branch of *w*-nullcline, creating another saddle-node bifurcation at (*I* = *I*_*app*_ = *I*_1_ ≈ 0.32, the red curve on Figure 5A), with a reverse sequence of fixed points annihilation. The model stops pacing at *I*_1_ and stays at high voltage due to the appearance of a stable fixed point between the right branch of *v*-nullcline and the upper branch of *w*-nullcline. This phenomenon is called the depolarization block in biology, and it plays a critical role in epilepsy (Connolly, 2016; Beck et al., 2019) or in blocking dopamine neurons (Richards et al., 1997; Lammel et al., 2008) for example. Figure 5C shows that the integrator model can spike at very low frequencies and blocks spiking exactly at *I*_1_. Note that the bifurcation of the depolarization block can be changed to more biologically plausible Poincaré–Andronov–Hopf bifurcation with a minor change in parameters given in Supplementary Code. Also, the same Supplementary Code shows how to include linear or non-linear adaptation into the model.

### 4.2. Resonant Neuron

Resonant neuron excitability, Hodgkin's class 2 excitability of giant axons, Type 2 excitability, or simply resonator, cannot spike sustainably at low frequencies. This type of excitability is usually phenomenologically modeled by a dynamical system with Poincaré–Andronov–Hopf bifurcation, also referred to as “Hopf.” A two-dimensional system undergoes this bifurcation when a stable fixed point loses stability, and a stable limit cycle appears around the unstable fixed point. A phenomenological model should undergo the subcritical Hopf bifurcation to model the phenomenon of sudden onset of high-amplitude oscillations.

We can recycle our integrating model (7). Because the dynamical system needs only one fixed point all the time for Hopf bifurcation, we can substitute *P*_3_() with a cubical polynomial with only two roots *P*_32_. To avoid any confusion, we will omit the *v*_1_ parameter in this model, where all other parameters will have the same meanings as in the integrating model. Therefore, the system became even simpler than 7):

(8) $$\begin{array}{l} \;L_{1}(v,v_{3},r_{0},r_{1},0)\dot{v}=P_{32}(v,v_{0},v_{2})L_{1}(v,v_{0},a_{0},a_{1},0)+I-w^{k}\\ S_{2}(v,v_{6},v_{7},s_{0},s_{1},s_{2})\dot{w}=L_{2}(v,v_{4},0,v_{5},1,0,0)-w \end{array}$$

We seek a system with only one fixed point. Because we chose only two roots in the polynomial, we can create a stable fixed point by setting *v*_4_ more hyperpolarized than *v*_0_. To maintain only one intersection with *v*-nullcline, we need to set the middle branch of the *w*-nullcline steeper than the unstable (middle) branch of *v*-nullcline. Finally, to have a reasonable range of currents between the onset of spiking and depolarization block, we slightly scaled-down the polynomial by the *a*_0_ coefficient. The *a*_0_ coefficient controls the overall scaling of the polynomial curve and, therefore, the decrease of *a*_0_ reduces the maximum of *P*_32_ function (the peak of the curve), and increases the gap between the *v*-nullcline and *w*-nullcline at higher voltages. As a result, the dynamical range of the model (where it spikes) increases and the model does not fall into a depolarization block shortly after the onset of spiking.

Overall, nullclines at rest are shown in Figure 5D (blue and black curves), while time-constants are the same for the integrator (Figure 5B). The resting potential can be found by solving the 4^*th*^ order equation analytically or numerically. An input current at the bifurcation point can also be found analytically or numerically. We used a semianalytical approach combining the *SymPy* symbolic solver to find Jacobian and *NumPy* to find its eigenvalues. Note that this analysis can be easily done in *PyDCTool* and in such software as *XPPAUTO* or *Matlab*. Nullclines at the bifurcation current are shown in Figure 5D (yellow and black curves). Finally, this model shows a depolarization block with saddle-node bifurcation (Figure 5D red and black curves) similar to the integrator. The dynamics of the resonant model with the same protocol as for integrator is shown in Figure 5E. Note that a firing rate around 4 spikes/s is the lowest frequency at which this model can oscillate. The reader can find two versions of adaptations that can be embedded in this model as well as a test for bistability in the Supplementary Code.

## 5. Example: Plateau-Potential in Phenomenological Neural Models

We have seen how the second, third, and fourth core principles of the framework work in biophysical and phenomenological models. However, it may not be obvious why we included the first requirement of continuity for model dynamics and did not relax the PLS-framework to include discontinuous models with resetting. This section will show an example of modeling a well-known biological phenomenon of a prolonged spikeless depolarization at about −20 mV called Plateau-potentials (PP) in five different phenomenological models. The section's main point is to show that the first core rule is essential and pivotal in the PLS approach.

In adult cortical pyramidal neurons, PP are generated in dendrites by NMDA glutamatergic receptors and are believed to play a crucial role in synaptic plasticity and learning (Bono and Clopath, 2017; Malik and Johnston, 2017). In the developing cortex, PP appears in response to spontaneous bursting activity generated in sensory organs and propagating into the cortex through the thalamus (Colonnese and Phillips, 2018). For example when a wave generated in the retina passes a location that projects to a specific thalamic relay neuron, the thalamocortical neurons' firing rates increase from 0.9 to 13 Hz (Murata and Colonnese, 2018). This increase in firing triggers neuron-wide PP in cortical neurons (Colonnese, 2014) (Figure 5A).

For the sake of simplicity, we considered a simple single-compartment phenomenological model, which receives inputs from 30 Poisson processes. The firing rate of sources is at 0.9 Hz all the time, except 1 s when it is 13 Hz. PP is a result of the non-linear activation of the dominant NMDA glutamate receptors. Therefore, we model two synaptic currents for each connection: a fast and weak AMPA current and a strong and slow NMDA current with magnesium block (see Materials and Methods for more details).

We first examined the two-dimensional model suggested by Izhikevich (2004, 2003, 2010). This model comprises a voltage variable with a quadratic polynomial on the right-hand side and a slow variable with a linear RHS. Because a quadratic polynomial does not have the second stable branch, the voltage increase is not limited. Therefore both the voltage and the slow variable must be reset to new values as soon as voltage crosses the 30 mV threshold. Parameters of Izhikeevich's model control time scale, the nullcline slope for the slow variable, and resetting values. The hybrid nature of this model allows mimicking different firing patterns of somatic spikes. Moreover, a modified version of Izhikevich's model was used for modeling active membrane properties in multicompartment models of hippocampal pyramidal neurons (Venkadesh et al., 2018); therefore, it is critical to test whether NMDA-driven PP can be reproduced in this dynamical system for modeling dendritic PP in these neurons.

Figure 5B1 shows voltage dynamics in Izhikevich's model in regular firing mode. We tried a few other modes, namely resonator, regular bursting, and chattering neurons, which produce similar firing patterns. None of those modes offers PP-like voltage dynamics. All of them produce bursts of spikes with unrealistically high firing rates. To reproduce PP-like voltage dynamics, the NMDA current needs to create a stable fixed point at potentials close to −20 mV. A synaptic current with zero reversal potential shifts *v*-nullcline to the right, bringing the minimum closer to zero voltage. As the *w*-nullcline must pass *w* = 0 at zero voltage in this model, and therefore the *w*-nullcline is not reachable for the stable branch of the *v*-nullcline. However, we did not pursue the further analysis of this model, but it seems unlikely that this system can lose and gain back stable intersection without non-linearity in *w*-nullcline.

A similar problem appears with an exponential leaky integrate-and-file model with linear adaptation (adExLIF) (Brette and Gerstner, 2005; Brette, 2015). This model combines linear (leaky) and exponential terms in the RHS of the voltage equation. The slow variable has a linear RHS; it is included in the voltage equation as a linear term. The behavior of adExLIF is similar to Izhikevich's model (Figure 5B2). Again because strong NMDA current shifts *v*-nullcline right, it is unlikely that there are parameter sets when the system loses and then gains stability with an increase of input current.

In contrast, an exponential leaky integrate-and-fire model with non-linear adaptation may potentially be able to reproduce PP. In this model, the slow variable controls the voltage at which the $dv/dt=\dot{v}$ changes the sign and exponentially increases (so-called knee point). Moreover, the slow variable non-linearly integrates membrane potential, and therefore, there may be a parameter set when prolonged depolarization promotes the slow variable to the value when a new stable fixed point appears at high voltage ($\dot{v}=ẇ=0$). Although it seems feasible to model PP in ExLIF with non-linear adaptation, we could not find an appropriate parameter set for that regime; therefore, we leave this question to the authors of the model and the readers. The dynamics of the membrane potential of non-linear adapting ExLIF with one of the parameter sets provided by the original publication (Fontaine et al., 2014) is shown in Figure 5B3. As with all other discontinuous models above, non-linear ExLIF in these parameters also produces an unrealistic high-frequency firing rate instead of PP.

**Figure 6:**
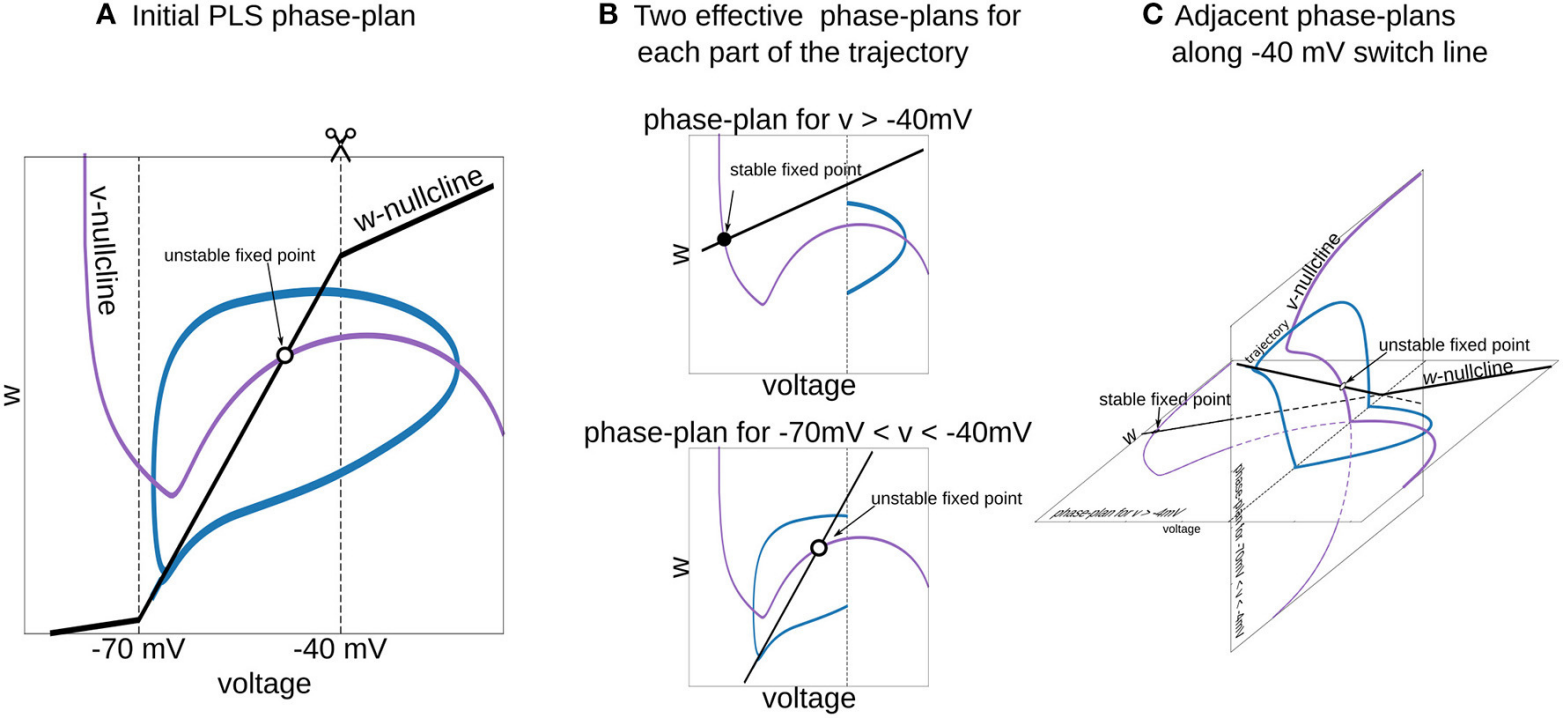
Interpretation of piecewise-Linear *w*-nullcline as a trajectory jumps between two phase-plans with pure linear *w*-nullclines. **(A)** The phase-plan of PLS model, similar to Figure 1C. Note that trajectory goes through two voltage ranges, where *w*-nullcline has different slopes. **(B)** Two phase-plans for each voltage range. The trajectory jumps from one phase-plan to another then crosses −40 mV line **(C)** Visualization of this two phase-plans as an adjacent spaces which intersect along −40 mV switching line. Horizontal phase-plan corresponds to the system when the voltage is higher than −40 mV and a vertical phase-plan for voltages between −70 and −40 mV. Similarly, for the *PL2D* reduction of the Wang and Buzsáki model (Figure 1), two phase-plans are *v*-*n* spaces.

One of the major advantages of the PLS-framework is the continuity of all dynamical variables. Instead of discontinuous variables, the governing system of equations switches from one continuous set of equations to another. One can imagine the trajectory jumps between adjoining phase-planes in regions with similar vector fields (see Interpretation of a Dynamical System With *L* and *S* Functions on the Right-Hand Side of Differential Equations in the Discussion and Figure 6). The continuous dynamics offers a critical property: close to bifurcation/fixed point—the dynamical system is continuous and simple due to reduction to just linear and polynomial functions. Therefore, for both phenomenological examples developed above (see Equations 7 and 8 in section 4), a strong NMDA current can “clamp” voltage just below zero millivolts. A dynamical system analysis shows that the NMDA current shifts the *v*-nullcline right, creating a stable fixed point at the intersection of two nullclines. This intersection is in the range of −30 to −10 mV, relatively close to the observed intracellularly *in vivo* values (Colonnese, 2014) (Figures 5B4,B5 for integrator and resonator, respectively).

Moreover, for both integrator and resonator, the system undergoes supercritical Hopf bifurcation, resulting in a gradual reduction of spike amplitude and spiraling into a stable fixed point. Such a behavior is a critical feature of PP's onset in the real neuron in the developing cortex, but with fewer periods of oscillation (spikes) before PP. Note that we use our phenomenological examples without any adjustment for modeling PP. Obviously, a better match for PP onset can be achieved with parameter fitting.

Overall this example illustrates the critical requirement for consideration of a continuous dynamical system of neural models. Understanding this requirement and, at the same time, searching for a simple, fast, and scalable framework led to the development of the PLS-approach.

## 6. Discussion

We suggested here a simple, fast, and scalable framework that consists of three simple and computationally inexpensive families of functions, namely Polynomial, piecewise-Linear, and Step functions (PLS). By combining these well-known functions such that specific points of the discontinuous functions (*L* and *S*) are aligned with the minima or maxima of the *P* functions allows of the generation of a continuously differentiable right-hand side of the differential equations at fixed and bifurcation points. Although some discontinuous models can be formally defined in PLS-framework (for example Izhikevich's model is a *P*_2_ function for voltage and a *L*_1_ function for the slow variable), we constrained our PLS-framework to continuous dynamics modeling only (section 3) and showed an example to support that constraint. Each PLS function can be performed in a fixed number of elementary operations and can be easily mapped on hardware and multithreaded software. We provided two use-case examples for conductance-based [Level III detailed model in classification (Herz et al., 2006)] and phenomenological (Level III–IV) models to show the main reasoning behind this approach. Using the first example as a benchmark, we showed that PLS-framework allows both 3- to 5-fold speed-up of computation and minimal, <50 single or double precision numbers memory footprint, while keeping the simulation results close to fully-computed original mode with maximal absolute error <4% of the model dynamic range (Figure 2). We believe that implementation of this framework will make it possible to model slow evolving processes (like cortical development) with biophysically accurate representations of single neurons, will enable long simulations of large neural networks on modern desktop computers, and will speedup biophysically accurate neuron models to the level when they can be used for real-time applications of spiking neural networks in robotics and AI.

### 6.1. Modeling Slowly Evolving Processes During Cortical Circuitry Development

The development of thalamocortical circuits is a complex process that spans a few weeks to years depending on the species. During this timeframe there are massive changes in the membrane electrical behavior of individual neurons which, in combination with synaptic rearrangements, transform network dynamics (Colonnese and Phillips, 2018). One of the developing cortex's critical features is the rapid change in intrinsic excitability and firing patterns in primary cortical neurons (McCormick and Prince, 1987; Etherington and Williams, 2011; Kroon et al., 2019). During development neurons decrease action potential duration while increasing amplitude, develop active conductances leading to the emergence of cell-type specific firing behaviors, and become less electrically compact (McCormick and Prince, 1987; Luhmann and Prince, 1991; MacLeod et al., 1997). Early circuits are characterized by high excitatory convergence and absent or even excitatory GABAergic inputs (Chen and Regehr, 2000; Ben-Ari et al., 2007; Murata and Colonnese, 2020). As result of these early cellular and circuit configurations the developing thalamocortex produces unique patterns of neural activity, such as “spindle-bursts” and “early gamma” which are generated in response to spontaneous activity in the sense organ (Leighton and Lohmann, 2016; Luhmann and Khazipov, 2018; Blumberg et al., 2020). *In-vivo* recordings in the maturating visual cortex showed intracellular membrane potential dynamics during the early developmental period that include prominent plateau potentials at the neural cell body: prolonged spikeless depolarizations with the membrane potential above −20 mV during spindle-bursts (Colonnese, 2014). Similar potentials are observed in the relay thalamus, where they are critical to the normal development of thalamic circuits (Dilger et al., 2011, 2015). Interestingly, plateau-potentials have also been observed in the basal dendrites of adult cortical pyramidal neurons where they play a pivotal role in synaptic plasticity (Gambino et al., 2014; Bono and Clopath, 2017; Malik and Johnston, 2017). The modeling of these phenomena during development is specifically challenging because it requires long simulations of large-size networks of cortical and thalamic neurons with the dynamics of each neuron reconstructed with enough fidelity for modeling plateau-potentials and network spindle-burst oscillations. We show here (Figures 1, 2, 5) that a PLS-approach allows model fidelity close to the fully-computed model, but with acceleration and small memory footprint sufficient to enable long enough simulations to study network formation during the development period.

The PLS-framework represents complex steady-state and tau functions as a combination of simpler *P*, *L*, and *S* functions. A PLS representation breaks full ranges of dynamic variables into segments between specific points of *L* and *S* functions, where the system comprises just linear and polynomial right-hand sides. In other words, a PLS representation segments the phase-space of the model into easier analyzable pieces. This may enable comparison between developmental stages and help answer questions, such as what is the role of different channels in developing neuron excitability, and what are the rules for channel homeostasis during cell development? Potentially, tracking similar phase-space segments at different ages will show the contribution of different channels at each segment and elucidate the evolution of this contribution along the developmental trajectory.

### 6.2. Continuous vs. Discontinuous Dynamics in Phenomenological Models

One of the salient features of PLS-framework is the explicit requirement of continuous dynamics (the first core principle in section 3). We demonstrated the importance of this requirement in the example in section 5. Note that any continuous phenomenological model, such as FitzHugh-Nagumo (FitzHugh, 1955, 1961) or Morris-Lecar (Morris and Lecar, 1981) can reproduce plateau-potentials, just because of the continuity of the dynamical system. On the other hand, models with resetting can reproduce phenomena which are not possible to model if the system is continuous and low-dimensional; for example, a model the intrinsic bursting cell-type requires a 3D continuous system (Hindmarsh and Rose, 1984; Drion et al., 2015; Knowlton et al., 2020), but can be reproduced in 2D discontinues models (Izhikevich, 2003, 2004; Brette and Gerstner, 2005; Destexhe, 2009). The hybrid nature of discontinuous models, which mix continuous dynamics with mapping [resetting (Izhikevich, 2010)], reduces the number of differential equations and the required elementary operations per second of model time. Although hybridization allows some authors to achieve astonishing precision in somatic voltage behavior at the onset of the spike (Brette, 2015), long-term spike prediction for somatic noise current injection (Gerstner and Naud, 2009), or visual similarity of spike patterns with intracellular recordings (Izhikevich, 2003, 2004; Destexhe, 2009), the real neuron dynamics are continuous and do not have any discontinuity. Therefore, hybrid models can catch the essence of neural dynamics in some subranges (spike initiation for continuous equations or spike-patterns for mapping). However, there is no hope that they cover the full range. That is true specifically for slow dendritic dynamics with calcium spike propagation and prolonged spikeless depolarizations, such as plateau-potentials or a depolarization block. The PLS-framework suggests another approach: keep the system dynamics **continuous** without resetting. Instead of a hybrid of continuous and discontinuous dynamic variables (for example, voltage—continuous until reach threshold and then discontinuously reset), the PLS-framework uses continuous and discontinuous functions on the right-hand side of differential equations but keeps all dynamic variables continuous. The only one potentially negative consequence of our approach is that the PLS instantiations demonstrated in section 4 could not generate intrinsic bursts in response to applied current injection. Both integrator and resonator are 2D continuous systems, and therefore, they require at least one additional differential equation, a “super slow” dynamic variable for controlling burst duration and spiking termination. Although an introduction of such a dynamic variable is not a problem, it requires additional computations, while hybrid models can burst without extra computational expenses.

### 6.3. PLS vs. Lookup-Table Approximations

Although different model approximations in section 2 aim to introduce the PLS concept, it may also raise a question: do we really need another framework, or is a lookup table approximation is good enough? To answer this question, one needs to consider at least two critical points: First, as we mentioned above, the lookup table size cannot fit into even middle-range microcontroller memory. The reduction of rows in the table may help to solve this problem at first glance. However, to reach the same accuracy as the PL2D approximation, the lookup table needs at least 20 rows, which requires almost three times more memory than the PL2D approach (see Supplementary Figure 2). In the case when computations are performed on a large network of lightweight microprocessors or microcontrollers, like SpiNNaker (Furber et al., 2013), where each neuron or very small subpopulation of neurons is computed at each node, having large tables for each channel can be challenging, even with a smaller footprint. Moreover, the “memory-hungry” algorithms may be a critical obstacle for GPU implementation of the heterogeneous network (see below), in which lookup tables should be precomputed for some subset of channels of each neuron independently.

The second critical disadvantage of the lookup table approximation can be clearly seen from the point of modeling brain network development. During the pre- and post-natal development, the excitability of neurons is gradually changing (McCormick and Prince, 1987; Kroon et al., 2019). This is presumably due to the maturation of compositions of subunits for many ion channels. To model development, one would have to recompute the lookup tables for each subunit composition, for each channel, at each development stage. In contrast, PLS-framework may need only adjustment of coefficients—a quick and simple change which can potentially be done “on the fly.”

### 6.4. Does PLS Framework Offer Good-Enough Model Fidelity?

As we stated several times above, PLS is a framework, which can be used in many different models and in many different ways; therefore, the model fidelity is the question of concrete realization and the concrete task at hand. The PLS-framework application and the choice of approximation accuracy depend upon how stable network dynamics and whether it operates closer to stability boundary. The closer the system is to this boundary, the better the accuracy of an approximation should be.

For example, during the first post-natal week in the rodent visual system, the spontaneous activity generated in the retina provides the initial drive for 90% of thalamic and cortical firing (Murata and Colonnese, 2016, 2019). In the absence of retinal drive the thalamocortical system remains fairly silent for many hours, if not days. Also, the thalamocortical network does not show prolonged “run-off” after the retinal burst and returns to the resting state after a few seconds of spiking. The approximation may not be perfect in this example, but the error will not accumulate due to resting between the bursts. Although the burst dynamics may be slightly different, the approximation must reproduce the salient features of the real neuron activity, such as plateau-potentials. Therefore it is critical to have a framework in which such an activity can be reproduced even in simple phenomenological models (see section 5).

What if the system is on the border of its stability, for example, a network of neurons in a “balance state,” when excitation and inhibition compensate each-other, keeping neurons in a subthreshold range with irregular firing? The general recommendation, in this case, is to construct an approximation in the PLS-framework with maximal accuracy in the subthreshold range of voltages, therefore reducing the accumulation error. Obviously, the approximation can diverge from the fully-computed model due to the non-linear nature of the neuron and network dynamics. However, the construction of an approximation with maximum accuracy in the most sensitive regions can mitigate this divergence or slow it down, at least. Giving such a recommendation, we should admit that at this moment, there are not good tools for the automated fitting of PLS functions to a given fully-computed model or detection of sensitive regions. These are the critical topics of future research and development in the PLS-framework (see below).

### 6.5. PLS Framework Can Improve Modeling Heterogeneous Population on GPU

Parallel computing on GPU, with single instruction multiple data (SIMD) architecture, can significantly accelerate many scientific computations from fluid dynamics to artificial intelligence. GPU can substantially speed up spiking neural networks (SNN) simulations and potentially be used to calculate biophysical accurate models. However, SIMD architecture, where each thread computes a single neuron, requires, if not homogeneous, but at least isotropic population of neurons, where each neuron has the same dynamic equations. The parameters of these equations can be different for each neuron. They are treated as data for each thread, which are passed through the same formulas. If one wants to accelerate GPU computations using a lookup table approximation, it can be easily done if all neurons within a population have the same steady-state and tau functions, i.e., the same composition of channels. The lookup table can then be precomputed and held in shared multiprocessor memory, which is serving for all threads. However, if the neuron population is truly heterogeneous and different neurons may express different subunits of ion channels and synaptic receptors (which resembles more closely the real brain networks), the lookup table should be precomputed for every subunit combination. Considering that the flagship NVidea Ampere (C) GPU vector accelerator has just 192 kB of combined super-fast shared memory and L1 cache per multiprocessor, these lookup tables could be fitted into larger but slower L2 40 mB cache or even move to DDR5 40 gB main memory. We believe it will significantly reduce the overall speed increase for lookup table approximation.

On the other hand, PLS approximation offers a much smaller memory footprint. In general, the heterogeneity in ion-channel compositions will create an independent set of parameters for each neuron, which can fit even in small but super-fast L1/shared-memory. Moreover, we anticipate that further development of the PLS-framework will identify a subset of nested functions for accurate approximation of sigmoid and bell-shaped functions (see below). This may potentially allow modeling even heterogeneous and anisotropic neuron populations on GPU multiprocessors. Again, this will be in the focus of future research and development of PLS-framework.

### 6.6. Interpretation of a Dynamical System With *L* and *S* Functions on the Right-Hand Side of Differential Equations

To choose a good approximation for the neural model in hand, it is useful to imagine a phase-portrait of a dynamical system. Discontinuity in RHS of differential equations can be a difficult obstacle for “mental manipulations” with such a system. In our practice, we usually consider *L* and *S* functions as a set of straight lines. For example, one can imagine the *n*_∞_(*v*) function in *PL2D* approximation as a set of 3 straight lines (branches of *L*_3_, Figure 1C). In a spiking regime, the trajectory goes into the range of voltages where only two of them are active. Therefore, one can imagine a trajectory jumping from one phase-plan with linear nullcline for the slow variable to another one (Figure 6). This figure shows that the system has a stable fixed point at higher voltages (black filled circle on the horizontal phase-plan). However, when the trajectory moves toward this stable fixed point and crosses −40 mV, it switches to the system with an unstable fixed point (open circle on the vertical phase-plane). Although these two nullcline configurations are very different, they have similar vector fields at the intersection line, and therefore, the phase-portrait for the simplified system is close to the original model. This example shows that appropriate specific points for *L* and *S* functions can be chosen from a consideration of a vector field close to the line (plan/space) at the intersection of two phase-plans (spaces).

### 6.7. Different *L* Approximations

There are two ways to construct an *L*-approximation. The first locates switching points on the curve (Hamann and Chen, 1994), creating lines at each arch base. The second approach uses linear regression for each arch and minimizes overall error for the approximation (Goncharenko and Gopinathan, 2008). However, the second *L*-approximation does not place switching points on the curve, and moreover, there is no guarantee that lines will intersect somewhere close to the segment borders. Although the first approximation can introduce a systematic error that is hard to debug, it can be performed automatically, while the second approach can reliably be used and find intersections only for strictly convex or concave curves (Goncharenko and Gopinathan, 2008). We were not able to develop a robust algorithm for reliable automatic construction of the second kind of L approximation for an arbitrary curve, which should be the topic for future research.

### 6.8. Order of *P*, Number of Branches in *L*, and Nested *S*(*P*) Functions

An increase in the order of *P* approximations and an increase in the number of branches in *L* and *S* functions can help improve model accuracy. However, while an increase in the number of rows in a lookup-table will not increase the number of elementary operations needed for computing one step of the simulation, an increase in order/branching of *P*, *L*, and *S* functions will add more computations in each step and slow-down simulations. Therefore, in practice, there is always a trade-off between accuracy and memory consumption for lookup-table approximation and a trade-off between accuracy and simulation speed for the PLS-framework.

For each additional order of *P* approximation, one multiplication and one addition operation is needed. Moreover one additional constant is required to be kept in the memory. Each additional branch of *L* and *S* functions adds at least three constants, one multiplication, one addition, and one comparison. Note that all operations in conditional statements are computed in modern GPU, but then the results are discarded due to the SIMD architecture. The order/branching for the *P*, *L*, and *S* functions should be carefully chosen to balance performance and model accuracy appropriately.

Alternatively, one can consider a nested *S*(*P*) function as a standard cubical-spline interpolation. For example *S*_3_(*v, V*_0_, *V*_1_, 0, *ψP*_32_(*v, V*_0_, *V*_2_), 1) can be a good approximation for standard sigmoidal (Boltzmann's) function. In this approximation, *ψ* scales the *P*_32_ function to have zero in the local minimum and one in local maximum, *V*_1_ is the argument of *P*_32_ in maximum, and *V*_0_ is an onset of and *V*_1_ a saturation of input-output transferring. Note that one can dramatically improve approximation accuracy without a heavy increase in orders or the number of branches in this case. This approximation is smooth and continuously differentiable at any point. We did not use such nested functions in this paper as our concern is to express the core principles of the PLS-framework, without including overcomplicated techniques. However, the PLS-framework is very flexible, and we encourage the usage of nesting *P*, *L*, and *S* functions.

### 6.9. Linear–Non-linear Models

The full range of models from Level I to Level IV can benefit from the usage of the PLS-framework, including cascade Linear–Non-linear (NL) models. NL models require at least one non-linear component for whole neurons, when it is a single compartment model or one non-linear component for each compartment, if it is a cascade NL model [for example, in each dendrite (Herz et al., 2006; Shai et al., 2015)]. Therefore NL significantly reduces the computational load for a single neuron compared with conductance-based multicompartment, multisegment models. The non-linear term in NL models is usually a sigmoid function with one exponential function in the denominator. However, taking into account the number of neurons and their diversity even in the same cortical region, this reduction in computation may not be enough for simulation of a large-scale model on a desktop computer. The PLS-framework offers reductions of the sigmoid function to *L*_3_–*L*_*n*_ non-smooth, multistep threshold function *S*_1_–*S*_*n*_, or nested *S*(*P*_32_) smooth approximations. In all cases, the number of elementary computations can be reduced by two orders of magnitude.

### 6.10. Future Research and Development of PLS-Framework

The main contribution of this paper is the introduction of the PLS-framework, its core functions and principles. We hope that the paper presents clearly the PLS-framework to a broad community of scientists and engineers and will speed up further development of tools and functions libraries in this framework. Here we outline the steps which should be the focus of future R&D.

- While we have uploaded Python, XPP, C/C++, and Brian-simulator implementations of core PLS function in the ModelDB record associated with this paper (see below), further optimization of the code is needed. We will launch a GitHub repository shortly after the publication of this paper and update the ModelDB record;
- Nested PLS functions can provide a better fit to standard sigmoidal (Boltzmann's) steady-state functions and bell-shaped tau functions. The development of a library that standardizes PLS approximations for these functions can significantly simplify applications of PLS-framework. The library above must be equipped with automatic tools to fit such standard approximations to the required functions;
- A toolkit that will detect all potential points of instability for PLS approximations is a critical step to the automated transformation of a fully-computed model into a PLS approximation;
- Finally, another fitting procedure should be developed to minimize overall error in model dynamics. In contrast to fitting independent steady-state and tau functions, this procedure will control the accuracy of the transformed model and help optimize the accuracy-speedup-memory footprint trade-off.

We deeply believe that the PLS-framework will be a useful and valuable tool in the computational neuroscience field, from detailed multi-compartment models to formal models and theoretical or semianalytical research.

## 7. Materials and Methods

For the benchmark simulations used in the first example, the original model and all its reductions were written in C-language and compiled with standard GCC 8.2 compiler under Linux operating system. Rasbian 2019-09-26 “buster-lite” version of Linux OS was used for Raspberry PI tests and Ubuntu 20.04 for desktop and GPU tests. Any graphical interfaces were disabled for all benchmarks, and only minimal necessary programs were running in the background during benchmark runs. Each benchmark was run ten times, the time of performance was recorded using standard Unix-shell command time, and user wall-time were collected. Although the minimum wall-time is reported in Figure 2B1 to avoid any issues from background processes, the differences between average and minimal time were below 1% and can be ignored.

Models for the second example were implemented as Python 3 scripts in the Jupyter-notebook environment.

Brian 2.4.2 simulator (Stimberg et al., 2014, 2019) was used for implementation models in section 5. The synaptic current was modeled as the sum of fast voltage-independent AMPA current and slow NMDA current with a magnesium block:

$$\begin{array}{l} \;I_{syn}=g_{ampa}s_{ampa}(E_{syn}-v)+g_{nmda}\\ \;(b_{nmda}-a_{nmda})(E_{syn}-v)/(1+[Mg^{2+}]e^{-0.062\;v/3.57})\\ \;{\dot{s}}_{ampa}=-s_{ampa}/\tau_{ampa}\\ {\dot{a}}_{nmda}=-a_{nmda}/\tau_{nmda1}\\ {\dot{b}}_{nmda}=-b_{nmda}/\tau_{nmda2} \end{array}$$

where *τ*_*ampa*_ = 2 ms, *τ*_*nmda*1_ = 1 ms, and *τ*_*nmda*2_ = 200 ms are time constants; *E*_*syn*_ = 0 mV is synaptic reversal potential; [*Mg*^2+^] = 1 is magnesium concentration. The conductance of NMDA receptors was set to be four times stronger than AMPA: *g*_*nmda*_ = 4*g*_*ampa*_ Simulations and code for this example also were developed as Python 3 scripts in the Jupyter-notebook environment.

## Code Availability Statement

The source code of the models and required scripts can be found in the supplementary zip archive. It will also be made publicly available via the ModelDB website (McDougal et al., 2017) after publication of this article: http://modeldb.yale.edu/266863.

## Data Availability Statement

The original contributions presented in the study are included in the article/Supplementary Material, further inquiries can be directed to the corresponding author/s.

## Author Contributions

MC provided experimental data and biologically motivated research, and provided the funding. RT-H designed the research, and performed the research and analysis. RT-H and MC wrote the paper.

## Conflict of Interest

The authors declare that the research was conducted in the absence of any commercial or financial relationships that could be construed as a potential conflict of interest.

## Supplementary Material

Supplementary Figure 1 Illustration of lookup table approximation, as a linear interpolation between values in the table (red lines at the base of each arch).

Supplementary Figure 2 Memory footprint **(A)** and accuracy **(B)** of lookup table approximation. Results for the PL2D approximation are also shown for comparison. Note that to reach the accuracy of the PL2D approximation, lookup table needs at least 20 rows, which requires three times larger memory footprint than PL2D one.

Supplementary Figure 3 The trajectory of the Wang and Buzsáki model in spiking regime on the *h*-*n* plane (black curve) and linear approximation of the trajectory obtained by linear regression (red line).

Supplementary Figure 4 Head-to-head comparison of voltage nullcline for original model 2D reduction (black curves) and PL2D reduction (red curves) at six different applied currents (*I*_*app*_).

Supplementary Data Sheet 1 Listing of Python 3 Jupyther-notebook page with all steps of model reduction in section 2.

Supplementary Data Sheet 2 Listing of Python 3 Jupyther-notebook page with two phenomenological models described in section 4.

Supplementary Data Sheet 3 Listing of Python 3 Jupyther-notebook page with all five model tests in section 5.
