# Supplementary figures and images for: Polynomial, piecewise-Linear, Step (PLS): A Simple, Scalable, and Efficient Framework for Modeling Neurons
Source: Front Neuroinform. 2021 May 6;15:642933. doi: 10.3389/fninf.2021.642933 (PMC8134741; doi:10.3389/fninf.2021.642933)

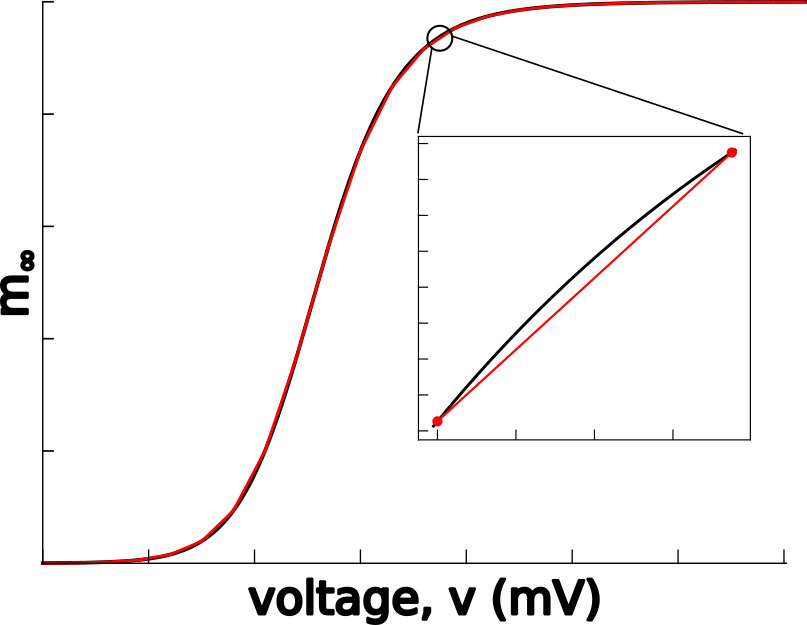

Supplement: Supplementary Figure 1 — Illustration of lookup table approximation, as a linear interpolation between values in the table (red lines at the base of each arch). [file Image_1.JPEG]

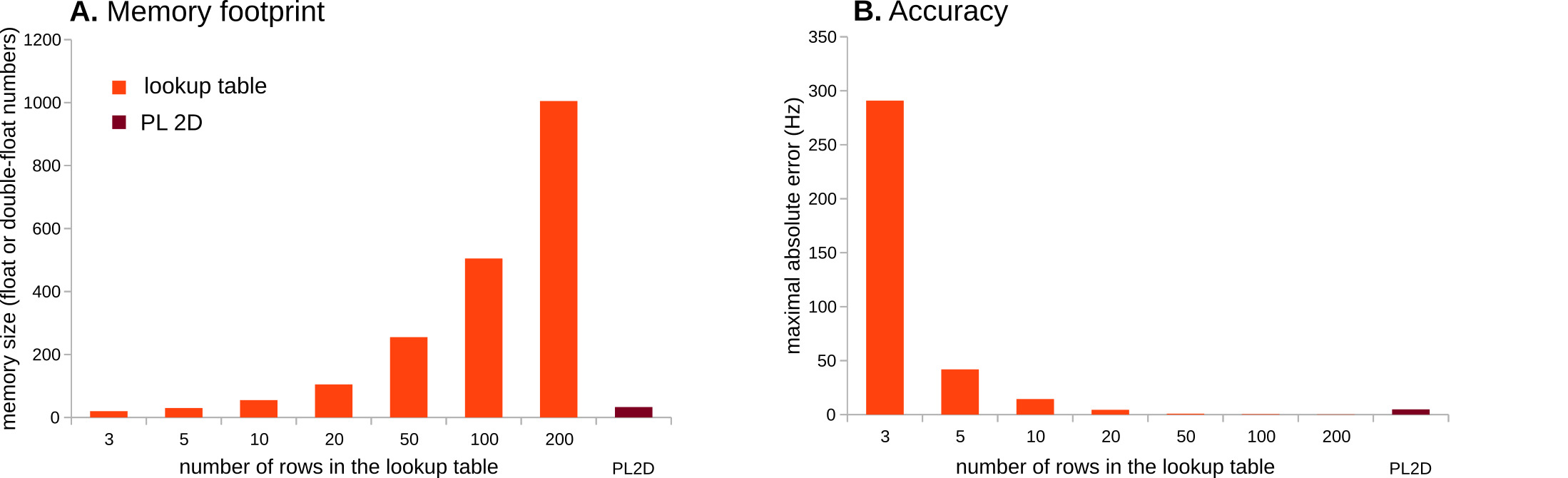

Supplement: Supplementary Figure 2 — Memory footprint (A) and accuracy (B) of lookup table approximation. Results for the PL2D approximation are also shown for comparison. Note that to reach the accuracy of the PL2D approximation, lookup table needs at least 20 rows, which requires three times larger memory footprint than PL2D one. [file Image_2.JPEG]

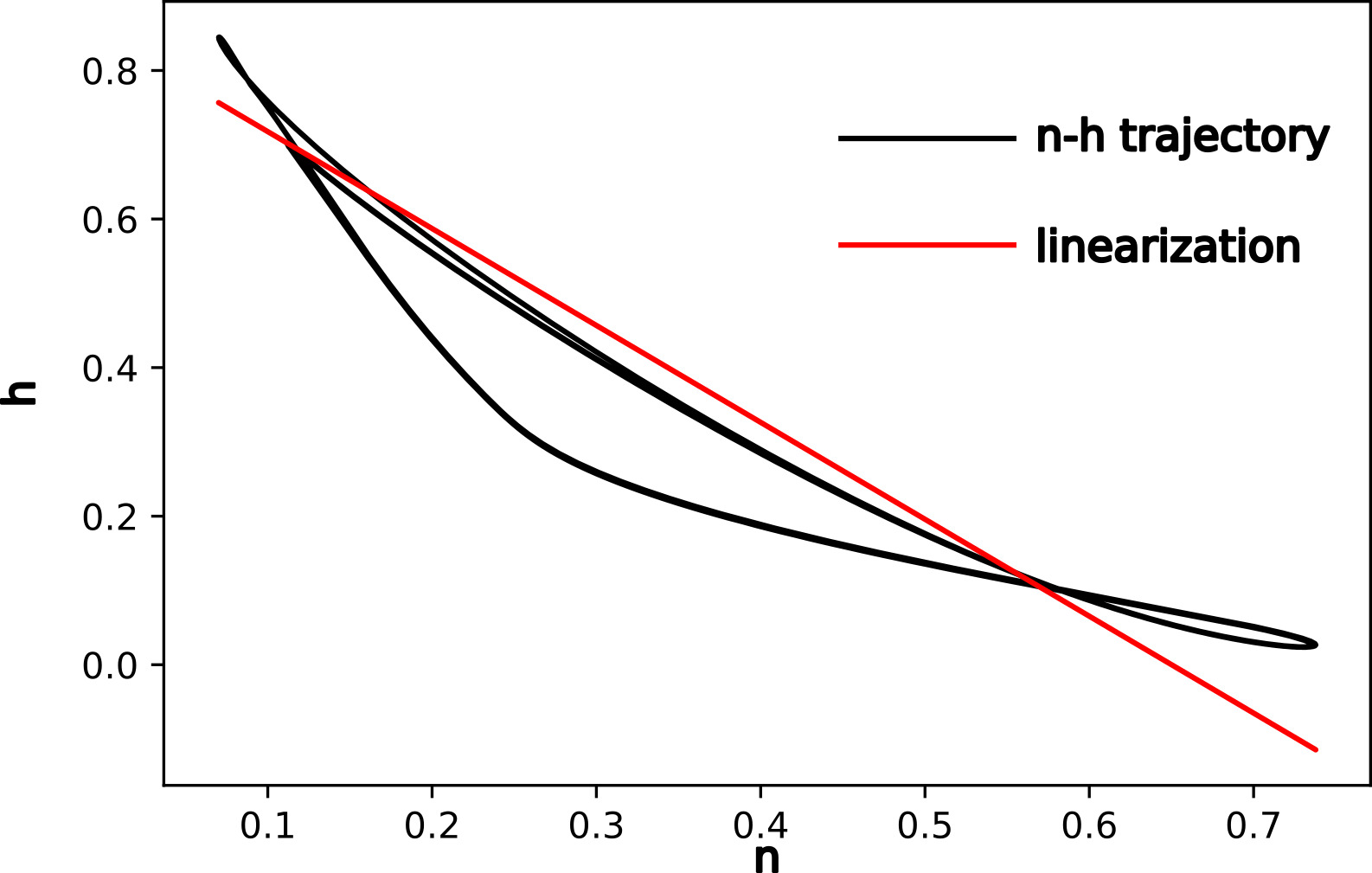

Supplement: Supplementary Figure 3 — The trajectory of the Wang and Buzsáki model in spiking regime on the h-n plane (black curve) and linear approximation of the trajectory obtained by linear regression (red line). [file Image_3.JPEG]

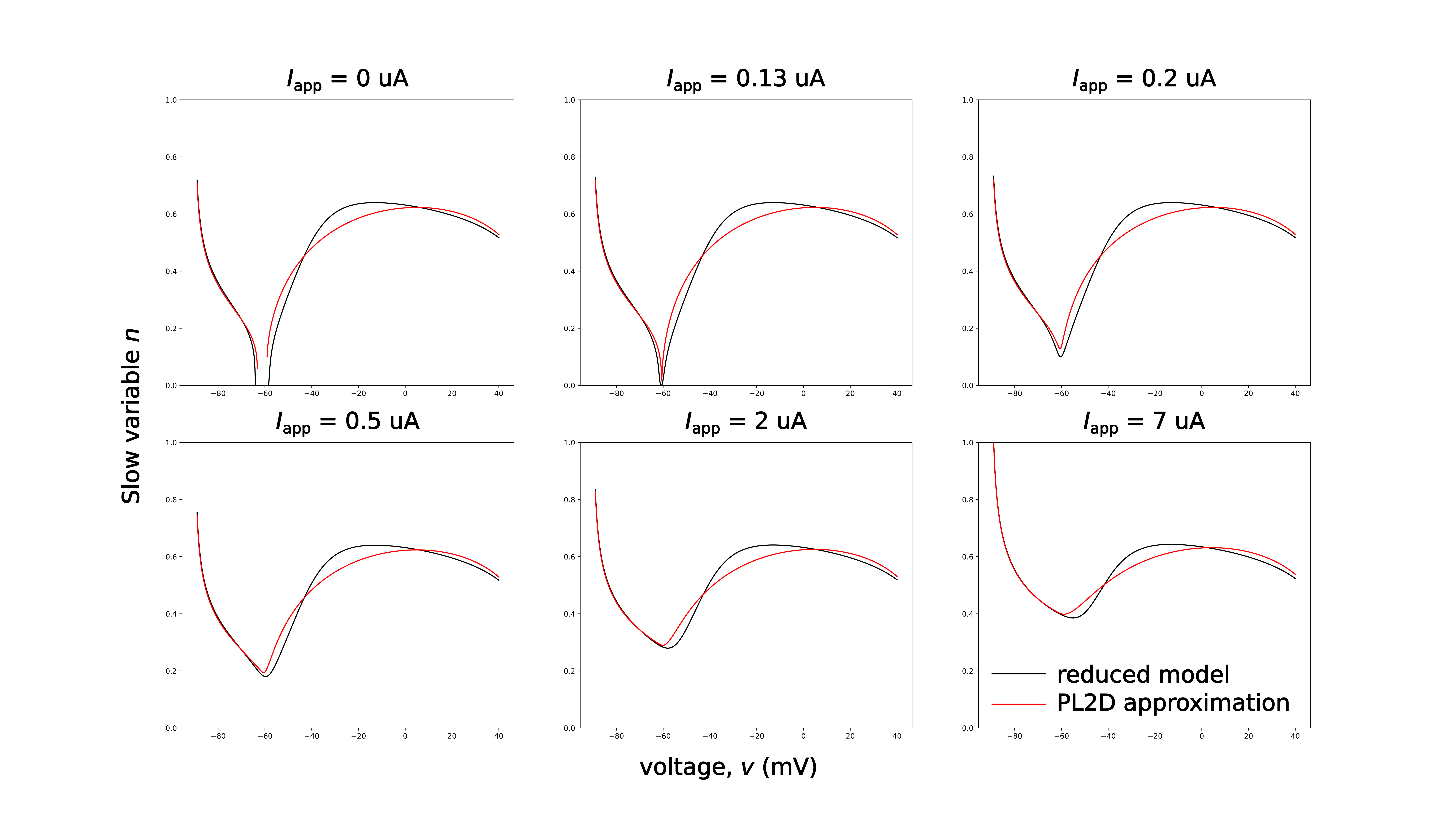

Supplement: Supplementary Figure 4 — Head-to-head comparison of voltage nullcline for original model 2D reduction (black curves) and PL2D reduction (red curves) at six different applied currents (Iapp). [file Image_4.JPEG]
