# Supplementary material for: Polynomial, piecewise-Linear, Step (PLS): A Simple, Scalable, and Efficient Framework for Modeling Neurons
Source: Front Neuroinform. 2021 May 6;15:642933. doi: 10.3389/fninf.2021.642933 (PMC8134741; doi:10.3389/fninf.2021.642933)
Supplement: Supplementary Data Sheet 1 — Listing of Python 3 Jupyther-notebook page with all steps of model reduction in section 2. [file Data_Sheet_1.PDF]

WangBuzsaki-model-reduction

March 1, 2021

1 Wang and Buzsaki Model

1.1 Voltage equation

$$\frac{dv}{dt} = I + g_l(E_l - v) + g_k n^4 (E_k - v) + g_{Na} m_\infty^3(v) h (E_{Na} - v) \tag{1}$$

1.2 Gate Variables

$$\frac{dn}{dt} = \phi * (\alpha_n(v)(1-n) - \beta_n(v)n) = \frac{\phi}{\tau_n} n_\infty - n \tag{2}$$

$$\frac{dh}{dt} = \phi * (\alpha_h(v)(1-h) - \beta_h(v)h) = \frac{\phi}{\tau_h} h_\infty - h \tag{3}$$

[17]:

```
%matplotlib inline
from mrth import *

v = linspace(-100.,80.,180)
phi = 5

am = lambda v:0.1*(v+35.)/(1.0-exp(-(v+35.)/10.))
bm = lambda v:4.0*exp(-(v+60.)/18.)
ah = lambda v:0.07*exp(-(v+58.)/20.)
bh = lambda v:1.0/(1.0+exp(-(v+28.)/10.))
an = lambda v:0.01*(v+34.)/(1.0-exp(-(v+34.)/10.))
bn = lambda v:0.125*exp(-(v+44.)/80.)

minf = lambda v:am(v)/(am(v)+bm(v))
ninf = lambda v:an(v)/(an(v)+bn(v))
hinf = lambda v:ah(v)/(ah(v)+bh(v))
ntau = lambda v:1. /(an(v)+bn(v))/phi
htau = lambda v:1. /(ah(v)+bh(v))/phi

figure(0,figsize=(22,12))
ax1=subplot(111)
lns1 = ax1.plot(v,vectorize(minf)(v),"r-",label=r"$m_{\infty}$",lw=4)
lns2 = ax1.plot(v,vectorize(ninf)(v),"b-",label=r"$n_{\infty}$",lw=4)
lns3 = ax1.plot(v,vectorize(hinf)(v),"g-",label=r"$h_{\infty}$",lw=4)
```

```
ax2 = ax1.twinx()
lns4 = ax2.plot(v,vectorize(ntau)(v),"y-", label=r"$\tau_{n}$",lw=4)
lns5 = ax2.plot(v,vectorize(htau)(v),"k-", label=r"$\tau_{h}$",lw=4)
lns = lns1+lns2+lns3+lns4+lns5
labs = [l.get_label() for l in lns]
ax1.legend(lns, labs, loc=1, fontsize=30)
ax1.set_ylim(0,1.1)
ax1.set_ylabel(r"Steady States: $m_{\infty}$,$h_{\infty}$,$n_{\infty}$",␣
 ↪fontsize=30 )
ax2.set_ylabel(r"Time Constants: $\tau_{h}$, $\tau_{n}$ (ms)", fontsize=30 )
ax1.set_xlabel("Voltage (mV)", fontsize=30 )
show()
```

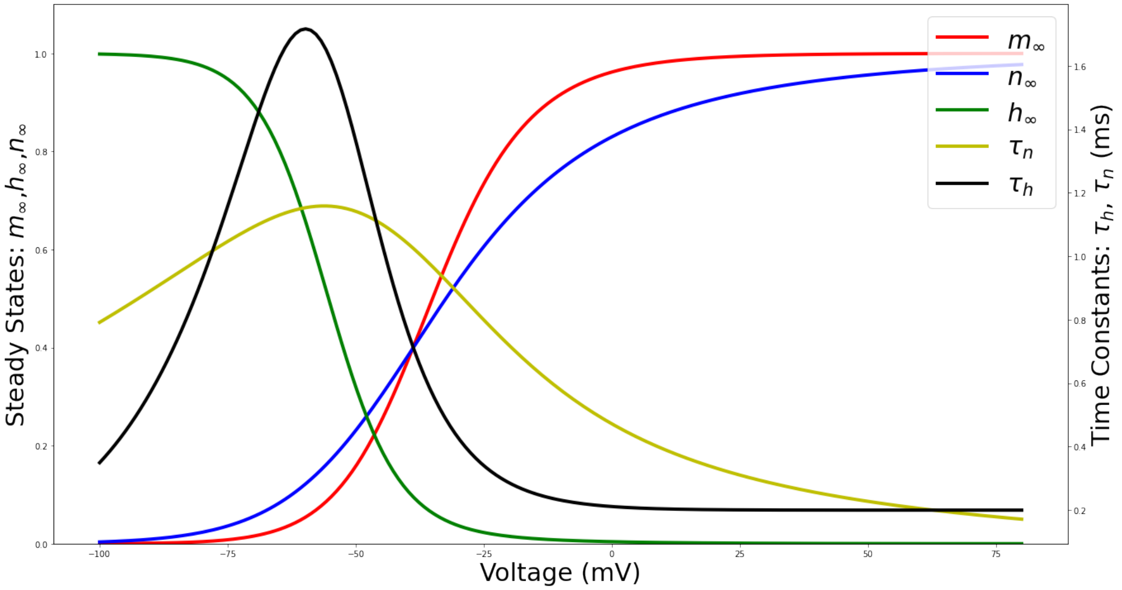

2 Part I: Complexity reduction.

2.1 Piecewise linearization

[18]:
```
"""
Two useful functions for curve fitting.
"""
linreg = lambda x,y: polyfit(x,y,1)
def pwlfit3(x,y):
    #First we want first order approximation, i.e. the liear regression
    b,y0 = linreg(x,y)
    # Another approach is a line from the beginning to the end of the interval
```

```
    #b=(y[-1]-y[0])/(x[-1]-x[0])
    #y0 = y[0]-b*x[0]
    #We are looking for maximums of mean squared deviation between original␣
 ↪curve and first order approximation
    drv =  (diff(sign(diff((y0+b*x - y)**2,n=2))) < 0).nonzero()[0]+1
    #For each interval between maximums we want to find linear approximation␣
 ↪again
    pwl = []
    li = 0
    for ri in drv:
        b,k = linreg(x[li:ri],y[li:ri])
        pwl.append( [b,k,x[li] ] )
        li = ri + 1
    b,k = linreg(x[li:],y[li:])
    pwl.append( [b,k,x[li] ] )
    #These linear regressions do not intersect in x[ri].
    #We have to recalcuate intersections
    for i in range(len(pwl)-1):
        pwl[i+1][2] = -(pwl[i+1][1]-pwl[i][1])/(pwl[i+1][0]-pwl[i][0])
    return pwl

def pwlfun(pwl, txt=False):
    #To minimize conditions, let's remove b and k for all 'right' slopes
    for i,(b,k,x0) in enumerate(pwl):
        for j in range(i+1,len(pwl)):
            pwl[j][0] -= b
            pwl[j][1] -= k
    #Now we can build a function:
    fn = "lambda x: ({}+{}*x)".format(pwl[0][1],pwl[0][0])
    for pro,pst in zip(pwl[1:-1],pwl[2:]):
        b,k,x0 = pro
        fn += "+({}+{}*x)*(x>{})".format(k,b,x0)
    fn += "+({}+{}*x)*(x>{})".format(pwl[-1][1],pwl[-1][0],pwl[-1][2])
    if txt: print( "PWL function :\n  ",fn )
    return eval(fn)

def polfun(pol, txt=False):
    """
    custom Polynomial function to be sure that both methods has the same␣
 ↪realization
    """

    fn = "lambda x: "
#### This gives the same speed as numpy version
#### So PWL is faster that 5th order
#    for i,c in enumerate(pol[::-1]):
```

```
#        fn += "{}".format(c) if i == 0 else "+{}*x".format(c) if i==1 else␣
↪"+{}*x**{}".format(c,i)

#### This is a much faster implementation
#### So PWL is slower than 5th order
    for i,c in enumerate(pol[::-1]):
        fn += "{}+x*(".format(c) if i != len(pol)-1 else "{}".format(c)
    for c in range(len(pol)-1):
        fn += ")"
    if txt: print( "POL function :\n  ",fn )
    return eval(fn)

##Here the check that numpy and custom functions yield the same results
#coef = np.polyfit(v,vectorize(minf)(v),pol_N)
#print coef
#fn = polfun(coef)
#plot(v, fn(v),"k-")
#plot(v,vectorize(pol_minf)(v),"r--",lw=3)
#show()
```

2.2 Fit $m_\infty(v)$, $h_\infty(v)$, $n_\infty(v)$, $\tau_h(v)$, and $\tau_n(v)$

[19]:
```
# m_inf
pwl_minf = pwlfit3(v,vectorize(minf)(v))
print( "\nMinf ", end="")
pwl_minf = pwlfun(pwl_minf,txt=True)

#h_inf
pwl_hinf = pwlfit3(v,vectorize(hinf)(v))
print( "\nHinf ", end="")
pwl_hinf = pwlfun(pwl_hinf,txt=True)

# n_inf
pwl_ninf = pwlfit3(v,vectorize(ninf)(v))
print( "\nNinf ", end="")
pwl_ninf = pwlfun(pwl_ninf,txt=True)
# tau_h
pwl_htau = pwlfit3(v,vectorize(htau)(v))
print( "\nHtau ", end="")
pwl_htau = pwlfun(pwl_htau,txt=True)
# tau_n
pwl_ntau = pwlfit3(v,vectorize(ntau)(v))
print( "\nNtau ", end="")
pwl_ntau = pwlfun(pwl_ntau,txt=True)
```

```
# =============== PLOT IT ! ==================
f1=figure(1,figsize=(22,12))
suptitle(r"Original functions vs. piecewise-Linear fit",fontsize=30)
subplot(321)
plot(v,vectorize(pwl_minf)(v),"k-",lw=3,label="piecewise-Linear fit")
plot(v,vectorize(minf)(v),"r-",lw=1,label="Original")
ylabel(r"$m_{\infty}$",fontsize=15)
xlabel(r"Voltage, v (mV)",fontsize=15)
legend(loc=0, fontsize=15)
subplot(322)
plot(v,vectorize(pwl_hinf)(v),"k-",lw=3)
plot(v,vectorize(hinf)(v),"r-",lw=1)
ylabel(r"$h_{\infty}$",fontsize=15)
xlabel(r"Voltage, v (mV)",fontsize=15)
subplot(323)
plot(v,vectorize(pwl_ninf)(v),"k-",lw=3)
plot(v,vectorize(ninf)(v),"r-",lw=1)
ylabel(r"$n_{\infty}$",fontsize=15)
xlabel(r"Voltage, v (mV)",fontsize=15)
subplot(324)
plot(v,vectorize(pwl_htau)(v),"k-",lw=3)
plot(v,vectorize(htau)(v),"r-",lw=1)
ylabel(r"$\tau_{h}$",fontsize=15)
xlabel(r"Voltage, v (mV)",fontsize=15)
subplot(325)
plot(v,vectorize(pwl_ntau)(v),"k-",lw=3)
plot(v,vectorize(ntau)(v),"r-",lw=1)
ylabel(r"$\tau_{n}$",fontsize=15)
xlabel(r"Voltage, v (mV)",fontsize=15)

ax1=subplot(326)
lns1 = ax1.plot(v,vectorize(pwl_minf)(v),"r-",label=r"PWL:$m_{\infty}$",lw=4)
lns2 = ax1.plot(v,vectorize(pwl_ninf)(v),"b-",label=r"PWL:$n_{\infty}$",lw=4)
lns3 = ax1.plot(v,vectorize(pwl_hinf)(v),"g-",label=r"PWL:$h_{\infty}$",lw=4)
ax2 = ax1.twinx()
lns4 = ax2.plot(v,vectorize(pwl_ntau)(v),"y-", label=r"PWL:$\tau_{n}$",lw=4)
lns5 = ax2.plot(v,vectorize(pwl_htau)(v),"k-", label=r"PWL:$\tau_{h}$",lw=4)
lns = lns1+lns2+lns3+lns4+lns5
labs = [l.get_label() for l in lns]
ax1.legend(lns, labs, loc=1, fontsize=15)
ax1.set_ylim(0,1.1)
ax1.set_ylabel(r"Steady States: $m_{\infty}$,$h_{\infty}$,$n_{\infty}$",␣
↪fontsize=15 )
ax2.set_ylabel(r"Time Constants: $\tau_{h}$, $\tau_{n}$ (ms)", fontsize=15 )
ax1.set_xlabel("Voltage (mV)", fontsize=15 )
#f1.savefig("WB-L.svg")
```

```
show()
```

```
Minf PWL function :
   lambda x: (0.03130775058256407+0.00032794170234354405*x)+(0.9842163211176729+
0.015406668782715469*x)*(x>-63.88248718774637)+(-0.0960057625861479+-0.014137559
007364468*x)*(x>-6.790830194670598)

Hinf PWL function :
   lambda x: (0.9617652153589281+-0.00037167628499264 77*x)+(-1.3260628344211058+
-0.016255777747902645*x)*(x>-81.57486248802813)+(0.39706401188385465+0.015850902
635657273*x)*(x>-25.0499306576171)

Ninf PWL function :
   lambda x: (0.09648374083111982+0.0009522189020595808*x)+(0.7430146812436749+0
.01046053058520931*x)*(x>-71.03030531685094)+(-0.03431219150587061 5+-0.008532156
604714406*x)*(x>-4.0215145004384425)

Htau PWL function :
   lambda x: (3.8998698682317046+0.03655767153938257*x)+(-4.738575320359597+-0.0
7845808930076661*x)*(x>-60.39626203735879)+(1.090615245017874+0.0407951344006680
65*x)*(x>-26.733953963882847)

Ntau PWL function :
   lambda x: (1.7804683137736268+0.00985969575027651*x)+(-1.2984598906696285+-0.
022548487217297717*x)*(x>-57.58523302057867)+(-0.049917117102098674+0.0088962276
4886577*x)*(x>5.611043137870116)
```

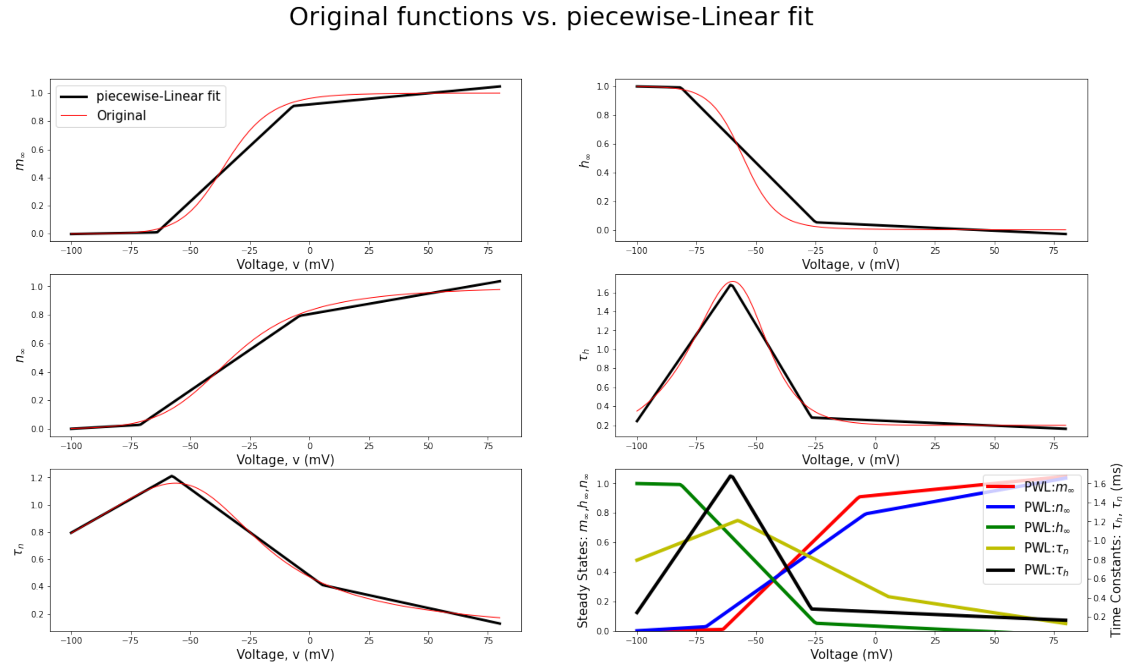

2.3 Polynomial Fit of Nth order

[20]:
```
pol_N = 5
print( "\nMinf", end="")
pol_minf = polfun(np.polyfit(v,vectorize(minf)(v),pol_N),txt=True)
print( "\nHinf", end="")
pol_hinf = polfun(np.polyfit(v,vectorize(hinf)(v),pol_N),txt=True)
print( "\nNinf", end="")
pol_ninf = polfun(np.polyfit(v,vectorize(ninf)(v),pol_N),txt=True)
print( "\nNtau", end="")
pol_ntau = polfun(np.polyfit(v,vectorize(ntau)(v),pol_N),txt=True)
print( "\nHtau", end="")
pol_htau = polfun(np.polyfit(v,vectorize(htau)(v),pol_N),txt=True)

# =============== PLOT IT ! ==================
f2 = figure(2,figsize=(22,12))
suptitle(r"Original functions vs. Polynomial fit of power %d"%pol_N,fontsize=30)
subplot(321)
plot(v,vectorize(pol_minf)(v),"k-",lw=3,label="Polynomial fit")
plot(v,vectorize(minf)(v),"r-",lw=1,label="Original")
ylabel(r"$m_{\infty}$",fontsize=15)
xlabel(r"Voltage, v (mV)",fontsize=15)
legend(loc=0, fontsize=15)
subplot(322)
plot(v,vectorize(pol_hinf)(v),"k-",lw=3)
plot(v,vectorize(hinf)(v),"r-",lw=1)
ylabel(r"$h_{\infty}$",fontsize=15)
xlabel(r"Voltage, v (mV)",fontsize=15)
subplot(323)
plot(v,vectorize(pol_ninf)(v),"k-",lw=3)
plot(v,vectorize(ninf)(v),"r-",lw=1)
ylabel(r"$n_{\infty}$",fontsize=15)
xlabel(r"Voltage, v (mV)",fontsize=15)
subplot(324)
plot(v,vectorize(pol_htau)(v),"k-",lw=3)
plot(v,vectorize(htau)(v),"r-",lw=1)
ylabel(r"$\tau_{h}$",fontsize=15)
xlabel(r"Voltage, v (mV)",fontsize=15)
subplot(325)
plot(v,vectorize(pol_ntau)(v),"k-",lw=3)
plot(v,vectorize(ntau)(v),"r-",lw=1)
ylabel(r"$\tau_{n}$",fontsize=15)
xlabel(r"Voltage, v (mV)",fontsize=15)
ax1=subplot(326)
```

```
lns1 = ax1.plot(v,vectorize(pol_minf)(v),"r-",label=r"POL%d:
↪$m_{\infty}$"%pol_N,lw=4)
lns2 = ax1.plot(v,vectorize(pol_ninf)(v),"b-",label=r"POL%d:
↪$n_{\infty}$"%pol_N,lw=4)
lns3 = ax1.plot(v,vectorize(pol_hinf)(v),"g-",label=r"POL%d:
↪$h_{\infty}$"%pol_N,lw=4)
ax2 = ax1.twinx()
lns4 = ax2.plot(v,vectorize(pol_ntau)(v),"y-", label=r"POL%d:
↪$\tau_{n}$"%pol_N,lw=4)
lns5 = ax2.plot(v,vectorize(pol_htau)(v),"k-", label=r"POL%d:
↪$\tau_{h}$"%pol_N,lw=4)
lns = lns1+lns2+lns3+lns4+lns5
labs = [l.get_label() for l in lns]
ax1.legend(lns, labs, loc=1, fontsize=15)
ax1.set_ylim(0,1.1)
ax1.set_ylabel(r"Steady States: $m_{\infty}$,$h_{\infty}$,$n_{\infty}$",␣
↪fontsize=15 )
ax2.set_ylabel(r"Time Constants: $\tau_{h}$, $\tau_{n}$ (ms)", fontsize=15 )
ax1.set_xlabel("Voltage (mV)", fontsize=15 )
#f2.savefig("WB-P.svg")
show()
```

```
MinfPOL function :
   lambda x: 0.9538819789279149+x*(0.007644362915280276+x*(-0.000176887049516215
57+x*(-1.9510874110134658e-07+x*(1.663851989829773e-08+x*(1.671055702310625e-11)
))))

HinfPOL function :
   lambda x: -0.031090447817729232+x*(0.0019265041362290661+x*(8.722279603254817
e-05+x*(-2.946886003553351e-06+x*(6.872964597465991e-11+x*(2.7067846046614394e-1
0)))))

NinfPOL function :
   lambda x: 0.8244944478680174+x*(0.0071982650648073805+x*(-0.00011073024032512
94+x*(-1.5311947856190683e-07+x*(9.398640449080108e-09+x*(3.033694426726568e-12)
))))

NtauPOL function :
   lambda x: 0.4924037413884465+x*(-0.012052097973031212+x*(0.000104713739320391
99+x*(1.6932665618325388e-06+x*(-1.4444933773685304e-08+x*(-1.1246206399456204e-
10)))))

HtauPOL function :
   lambda x: 0.16235026078929446+x*(-0.007686694069833764+x*(0.00029871171894912
344+x*(-8.228229274590399e-07+x*(-3.456921372430771e-08+x*(1.430040407066584e-10
)))))
```

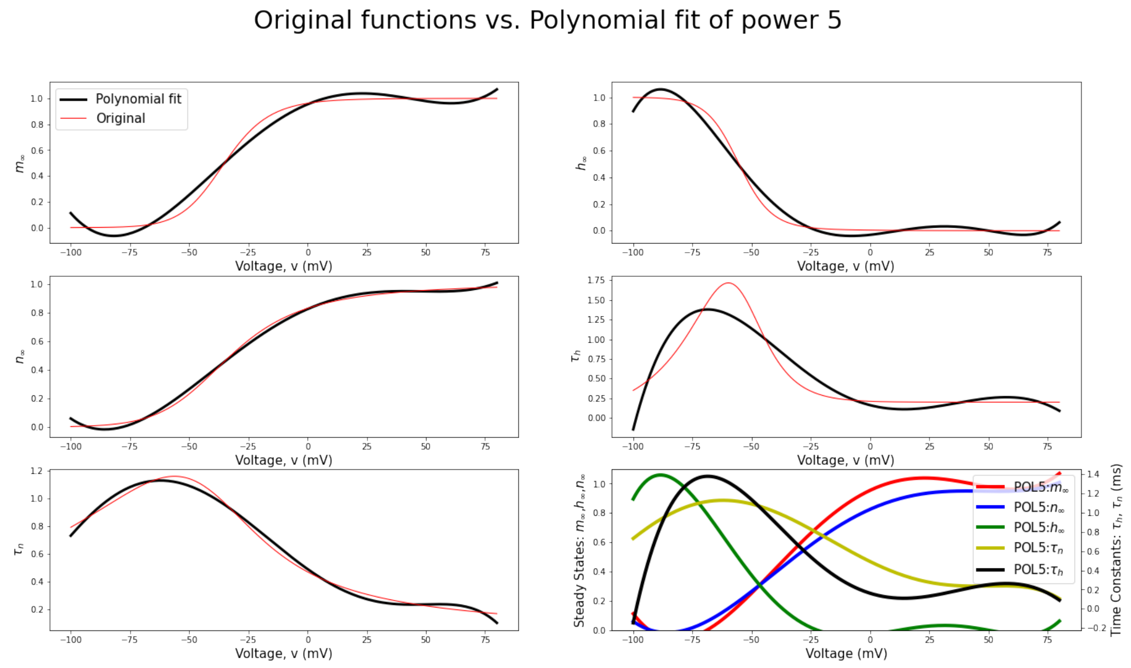

2.4 Solving with *SciPy*

[21]:
```
from scipy import integrate
#DB>
#print help(integrate)
#<DB
I=2.
El=-65.0
Ek=-90.0
ENa=55.0
gl=0.1
gk=9.
gNa=35
#    0  1  2  3  4  5
#Y=[v0,n0,h0,v1,n1,h1]
def rhs(Y,t):
    return[
        I*float(t>50.
 ↪)+gl*(El-Y[0])+gk*Y[1]**4*(Ek-Y[0])+gNa*minf(Y[0])**3*Y[2]*(ENa-Y[0]),
        (ninf(Y[0])-Y[1])/ntau(Y[0]),
        (hinf(Y[0])-Y[2])/htau(Y[0])
    ]

def pwl_rhs(Y,t):
    return[
```

```
        I*float(t>50.
↪)+gl*(El-Y[0])+gk*Y[1]**4*(Ek-Y[0])+gNa*pwl_minf(Y[0])**3*Y[2]*(ENa-Y[0]),
        (pwl_ninf(Y[0])-Y[1])/pwl_ntau(Y[0]),
        (pwl_hinf(Y[0])-Y[2])/pwl_htau(Y[0])
    ]

def pol_rhs(Y,t):
    return[
        I*float(t>50.
↪)+gl*(El-Y[0])+gk*Y[1]**4*(Ek-Y[0])+gNa*pol_minf(Y[0])**3*Y[2]*(ENa-Y[0]),
        (pol_ninf(Y[0])-Y[1])/pol_ntau(Y[0]),
        (pol_hinf(Y[0])-Y[2])/pol_htau(Y[0])
    ]
at=np.arange(0, 150.0, 0.01)
asol     = integrate.odeint(rhs,     [30.32243, 0.5407895, 0.1174041], at)
pwl_asol = integrate.odeint(pwl_rhs, [30.32243, 0.5407895, 0.1174041], at)
pol_asol = integrate.odeint(pol_rhs, [30.32243, 0.5407895, 0.1174041], at)

f3=figure(3,figsize=(20,15))
v = linspace(-100.,50.,180)
subplot2grid((5,5),(0,0))
plot(v,vectorize(minf)(v),    "k-")
plot(v,vectorize(pol_minf)(v),"-")
plot(v,vectorize(pwl_minf)(v),"-")
subplot2grid((5,5),(0,1))
plot(v,vectorize(hinf)(v),    "k-")
plot(v,vectorize(pol_hinf)(v),"-")
plot(v,vectorize(pwl_hinf)(v),"-")
subplot2grid((5,5),(0,2))
plot(v,vectorize(ninf)(v),    "k-")
plot(v,vectorize(pol_ninf)(v),"-")
plot(v,vectorize(pwl_ninf)(v),"-")
subplot2grid((5,5),(0,3))
plot(v,vectorize(htau)(v),    "k-")
plot(v,vectorize(pol_htau)(v),"-")
plot(v,vectorize(pwl_htau)(v),"-")
subplot2grid((5,5),(0,4))
plot(v,vectorize(ntau)(v),    "k-")
plot(v,vectorize(pol_ntau)(v),"-")
plot(v,vectorize(pwl_ntau)(v),"-")

subplot2grid((5,5),(1,0),colspan=5,rowspan=4)
plot(at, asol[:,0],    "k-",label="Original model")
plot(at, pol_asol[:,0], "-",label="Polynomial power %d model"%pol_N)
plot(at, pwl_asol[:,0], "-",label="piecewise-Linear model")

legend(loc=0)
```

```
xlim(0,150)

savefig("2.1-o-vs-l-vs-p.svg")
show()
```

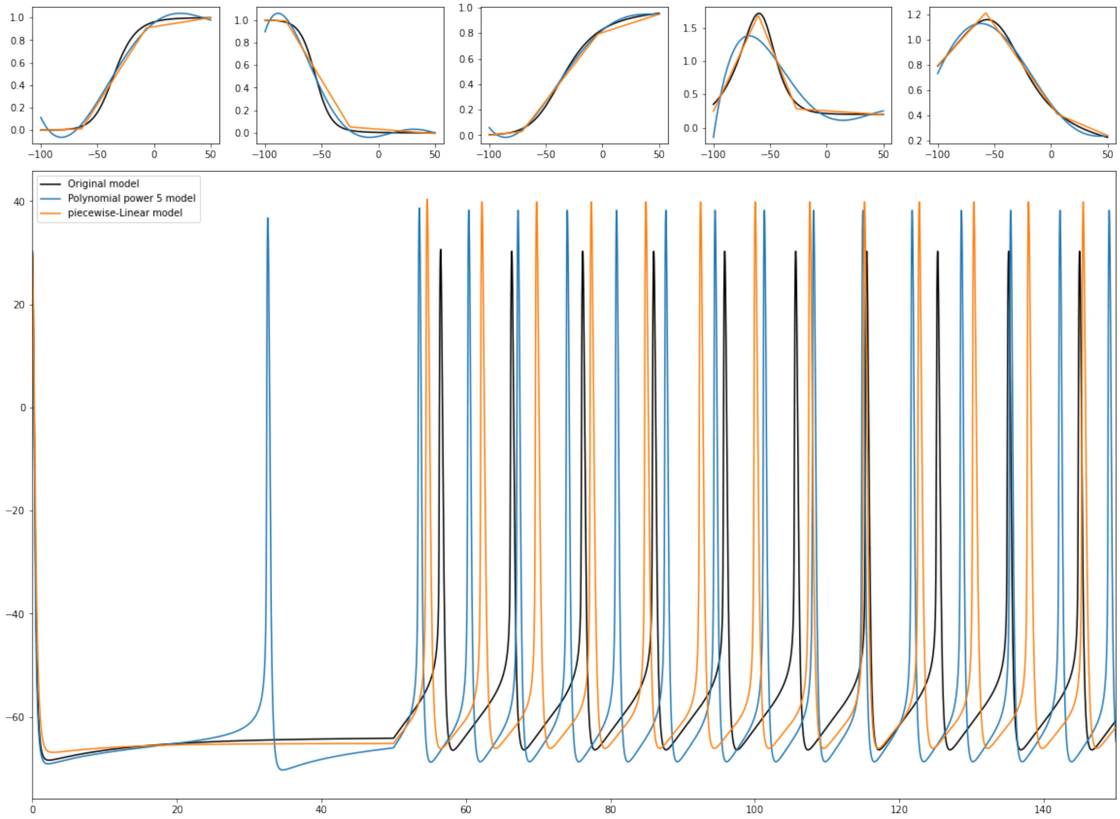

3 Part II Dimensionality Reduction

3.1 Standard h-to-n linearization

Let κ is a slope and h_0 is an offset of h vs. n - linean regression. The system became 2D

$$\frac{dv}{dt} = I + g_l(E_l - v) + g_k n^4 (E_k - v) + g_{Na} m_\infty^3(v)(\kappa n + h_0)(E_{Na} - v) \tag{4}$$

$$\frac{dn}{dt} = \phi * (\alpha_n(v)(1-n) - \beta_n(v)n) = \frac{\phi}{\tau_n} n_\infty - n \tag{5}$$

Let's find voltage nullcline by solving

$$I + g_l(E_l - v) + g_k n^4 (E_k - v) + g_{Na} m_\infty^3(v)(\kappa n + h_0)(E_{Na} - v) = 0$$

3.1.1 h-n slope and offset

[22]:
```
hn_slp,hn_ofs = linreg(asol[:,1],asol[:,2])
print( hn_slp,hn_ofs )
plot(asol[:,1],asol[:,2],"k-")
plot(asol[:,1], hn_ofs+asol[:,1]*hn_slp,"r-")
savefig("2.2-h-n-linearization.svg")
show()
```

```
-1.3046604849549275 0.8481147186576385
```

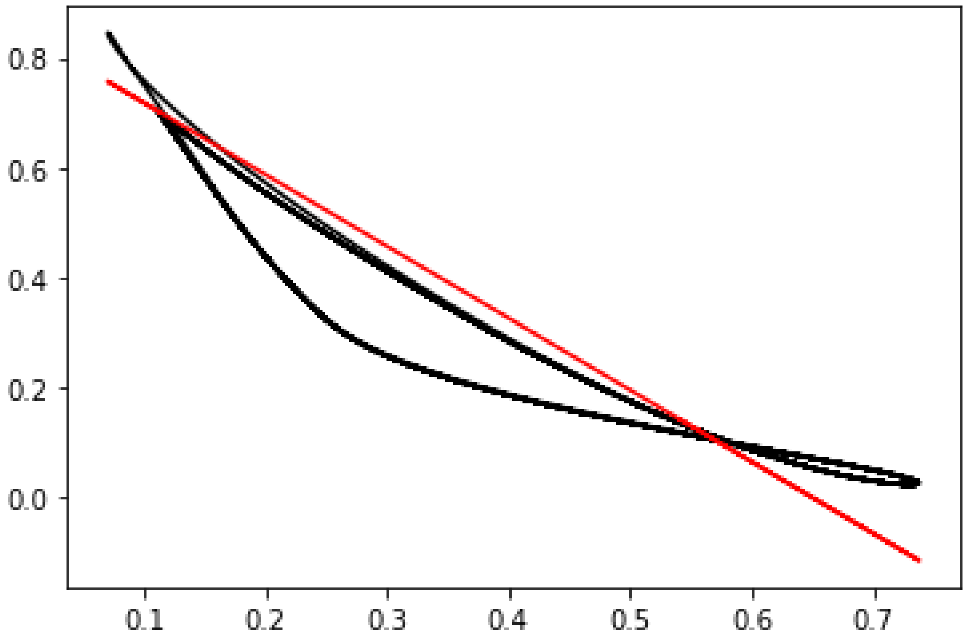

3.2 Nullcline approximation - mix model

[23]:
```
I=0#2#7.
El=-65.0
Ek=-90.0
ENa=55.0
gl=0.1
gk=9.
gNa=35
hn_slp, hn_ofs = -0.886966463871, 0.639251647788

def vfun(n,vm,I):
    return␣
 ↪I+gl*(El-vm)+gk*n**4*(Ek-vm)+gNa*minf(vm)**3*(hn_ofs+hn_slp*n)*(ENa-vm)
    #return I+gl*(El-vm)+gk*n**4*(Ek-vm)+gNa*minf(vm)**3*(1.-n)*(ENa-vm)
```

```
for I in 7,2,0.5,0.2,0:
    v0c=[]
    for vm in linspace(-89,40,200):
        n=sp.optimize.fsolve(vfun,0.5,args=(vm,I),xtol=0.01)[0]
        v0c.append((vm,n))
    v0c=array(v0c)
    plot(v0c[:,0],v0c[:,1],"-",label="v-nullcline,I={}".format(I))

vm=linspace(-89,40,150)
plot(vm,ninf(vm),"k-",label="n-nullcline")
legend(loc=0)
#ylim(0,1)
show()
```

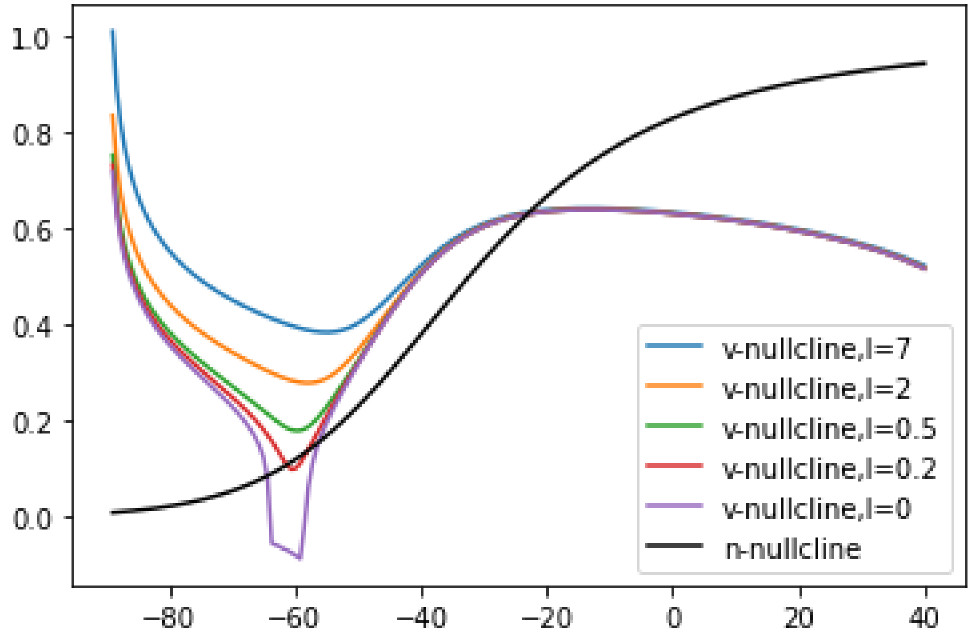

3.2.1 piecewise-Linear and Polynomial approximations

[24]:
```
v0c=[]
I=0.13
for vm in linspace(-89,40,2000):
    try:
        n=sp.optimize.fsolve(vfun,0.5,args=(vm,I),xtol=0.01)[0]
        v0c.append((vm,n))
    except:pass
v0c=array(v0c)
```

```
plot(v0c[:,0],v0c[:,1],"k-",label="2D-recuded nullcline",lw=2)
vc=v0c[:,0]
heav = vectorize(lambda x,x0:float(x>x0))
L2 =  vectorize( lambda x,s0,x0,y0,s1: (y0+s0*(x0-x)) if x<x0 else␣
↪(y0+s1*(x-x0)) )
L3 =  vectorize( lambda x,s0,x0,y0,x1,y1,s2: (y0+s0*(x0-x)) if x<x0 else (
    y0+(y1-y0)*(x-x0)/(x1-x0) if x<x1 else y1+s2*(x-x1)
       ) )

plot(vc,sqrt(sqrt((6e-4*(vc+60.6)**2*(55-vc))/(gk*(vc-Ek)                      ␣
↪ ))),"g-",label="Only polynomial approximation")
plot(vc,sqrt(sqrt((6e-4*(vc+60.6)**2*(55-vc))/(gk*(vc-Ek)*L2(vc,1.0,-60.6,1.,0.
↪0)))),"r--",label="piecewise-Linear and Polynomial approx.",lw=3)
#plot(vc,sqrt(sqrt((0.5e-6*(vc+60.6)**2*(55-vc))                        ␣
↪)),"b-",label="Only polynomial approximation")
#plot(vc,sqrt(sqrt((0.5e-6*(vc+60.6)**2*(55-vc))*L3(vc,2.,-80,0.7,-60.6,2.5,-1.
↪6e-2))),"r--",label="Piecewise and Polynomial approx.",lw=3)
legend(loc=0)
ylim(0,1)
savefig("2.2-pl-reduction.svg")
show()
```

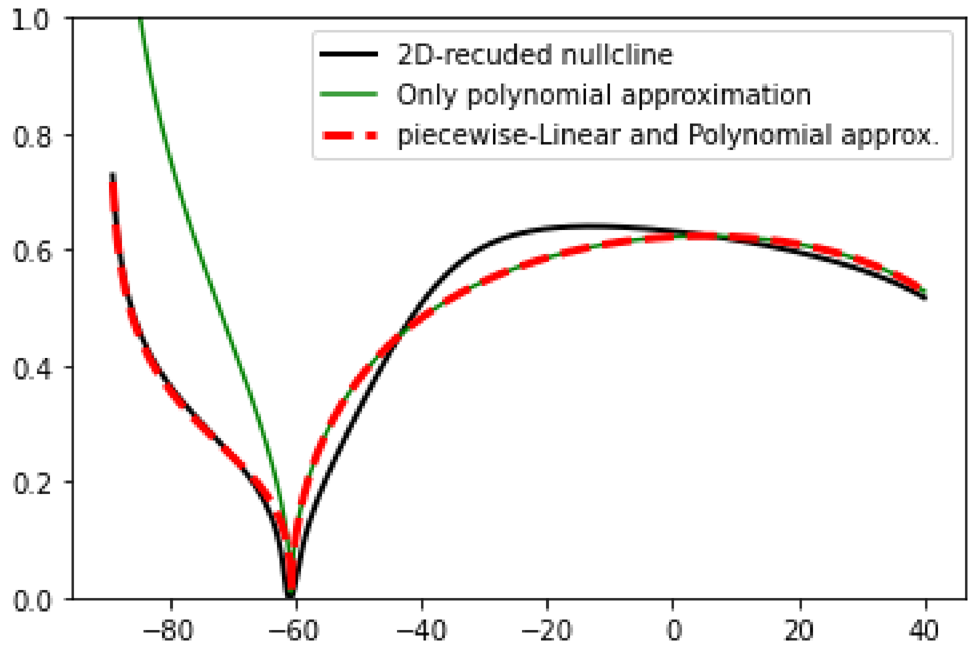

[25]:
```
def mixV0(vc,I):
    v0 = (I-0.13+6e-4*(vc+60.6)**2*(55-vc)/L2(vc,1.,-60.6,1.,0.0))/(gk*(vc-Ek))
```

```
    if v0 < 0: return nan,nan
    return vc,sqrt( sqrt(v0) )


mixV0 = vectorize(mixV0)
figure(1,figsize=(28,16))
Iapp = [0., 0.13, 0.2,0.5,2,7]
for i,I in enumerate(Iapp):
    subplot(231+i)
    v0c=[]
    for vm in linspace(-89,40,501):
        n=sp.optimize.fsolve(vfun,0.5,args=(vm,I),xtol=0.01)[0]
        v0c.append((vm,n))
    v0c=array(v0c)
    plot(v0c[:,0],v0c[:,1],"k-")
    #print mixV0(v0c[:,0],I)
    v0m = array(mixV0(v0c[:,0],I)).T
    plot(v0m[:,0],v0m[:,1],"r-")
    ylim(0.,1.)
show()
```

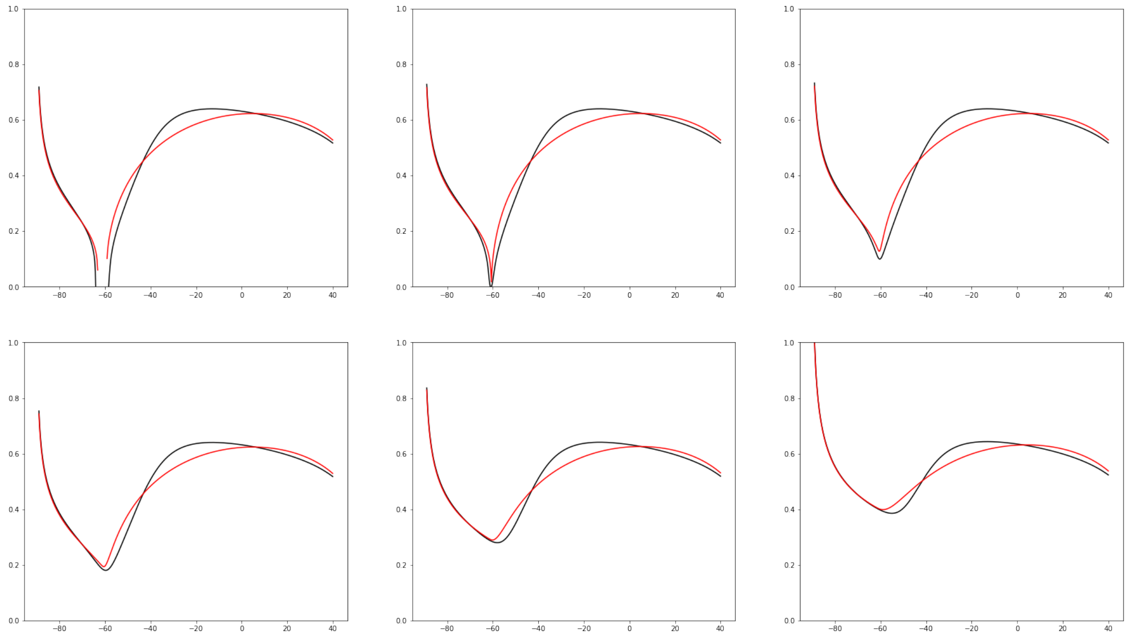

[26]:
```
figure(1,figsize=(20,5))
ax = subplot(131)
v0c=[]
I=0.13
for vm in linspace(-89,40,2000):
```

```
    try:
        n=sp.optimize.fsolve(vfun,0.5,args=(vm,I),xtol=0.01)[0]
        v0c.append((vm,n))
    except:pass
v0c=array(v0c)

plot(v0c[:,0],v0c[:,1],"k-",label="2D-recuded nullcline",lw=2)
plot(vc,sqrt(sqrt((6e-4*(vc+60.6)**2*(55-vc))/(gk*(vc-Ek)
 ↪ ))),"g-",label="Only polynomial approximation")
plot(vc,sqrt(sqrt((6e-4*(vc+60.6)**2*(55-vc))/(gk*(vc-Ek)*L2(vc,1.0,-60.6,1.,0.
 ↪0)))),"r--",label="Piecewise and Polynomial approx.",lw=3)
#plot(vc,sqrt(sqrt((0.5e-6*(vc+60.6)**2*(55-vc))
 ↪)),"b-",label="Only polynomial approximation")
#plot(vc,sqrt(sqrt((0.5e-6*(vc+60.6)**2*(55-vc))*L3(vc,2.,-80,0.7,-60.6,2.5,-1.
 ↪6e-2))),"r--",label="Piecewise and Polynomial approx.",lw=3)
legend(loc=0)
#ylim(0,1)

subplot(132,sharex=ax, sharey=ax)
#title("Classical model",fontsize=32)
for I in 0.,0.13,0.2,0.5,2.,7.:
    v0c=[]
    for vm in linspace(-89,40,200):
        n=sp.optimize.fsolve(vfun,0.5,args=(vm,I),xtol=0.01)[0]
        v0c.append((vm,n))
    v0c=array(v0c)
    plot(v0c[:,0],v0c[:,1],"-",label="v-nullcline, I={}".format(I),lw=3)

vm=linspace(-89,40,150)
plot(vm,ninf(vm),"k-",label="n-nullcline",lw=3)
legend(loc=0,fontsize=22)
ylim(0,1)

subplot(133,sharex=ax, sharey=ax)
#title("Mixed 2D model",fontsize=32)
for I in 0.,0.13,0.2,0.5,2.,7.:
    vm = linspace(-89,40,2000)
    v0m = array(mixV0(vm,I)).T
    plot(v0m[:,0],v0m[:,1],"-",label="v-nullcline,I={}".format(I),lw=3)

vm=linspace(-89,40,150)
plot(vm,pwl_ninf(vm),"k-",label="n-nullcline",lw=3)
legend(loc=0,fontsize=22)
ylim(0,1)
savefig("2.2-2DPL-nullclines-reduction.svg")
show()
```

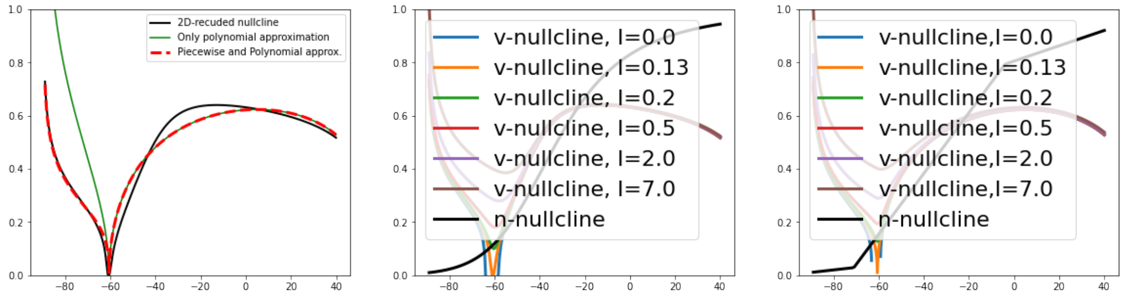

3.2.2 voltage time constant τ_v

Consider equation for voltage

$$\dot{v} = \frac{1}{c}\left[I_{app} - \bar{g}_{Na} m_\infty^3(v) h (v - E_{Na}) - \bar{g}_K n^4 (v - E_K) - g_L (v - E_L)\right]$$

Open parentheses and group terms to separate v

$$\dot{v} = \frac{\bar{g}_{Na} m_\infty^3(v) h + \bar{g}_K n^4 + g_L}{c}\left[\frac{I_{app} + \bar{g}_{Na} m_\infty^3(v) h E_{Na} + \bar{g}_K n^4 E_K + g_L E_L}{\bar{g}_{Na} m_\infty^3(v) h + \bar{g}_K n^4 + g_L} - v\right]$$

Therefore we can define:

$$\tau_v = \frac{c}{\bar{g}_{Na} m_\infty^3(v) h + \bar{g}_K n^4 + g_L}$$

and

$$v_\infty = \frac{I_{app} + \bar{g}_{Na} m_\infty^3(v) h E_{Na} + \bar{g}_K n^4 E_K + g_L E_L}{\bar{g}_{Na} m_\infty^3(v) h + \bar{g}_K n^4 + g_L}$$

[27]:
```
#Using simulation above let's find tau and linearly approximate it.
vtau  = 1./(gNa*minf(asol[:,0])**3*asol[:,2]+gl+gk*asol[:,1]**4)
vinf  = (2.+gNa*minf(asol[:,0])**3*asol[:,2]*ENa+gl*El+gk*asol[:,1]**4*Ek)*vtau
xvtau = vectorize(lambda v:0.4+(-38-v)*0.032*float(v<-38))

figure(figsize=(10,8))

plot(asol[:,0],vtau)
plot([-38,30],[0.4,0.1],"r--",lw=3)
plot([-60,-38],[7.8,0.4],"r--",lw=3)
plot(asol[:,0],xvtau(asol[:,0]),"b--")
#plot(asol[:,0],ntau(asol[:,0]),"y-")
#legend(loc=0,fontsize=16)
show()
```

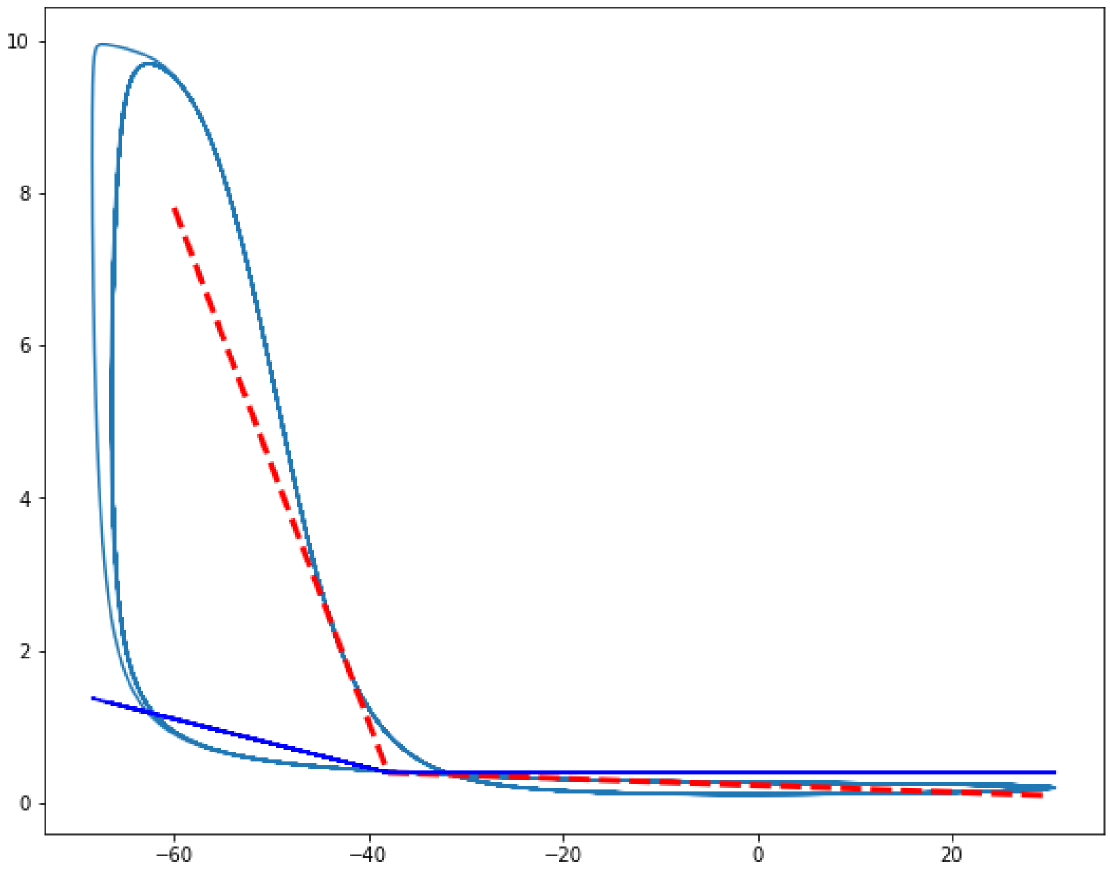

[28]:
```
from scipy import integrate
xvtau = lambda v:0.4+(-38-v)*0.032*float(v<-38)

I=2.#1.#7.
El=-65.0
Ek=-90.0
ENa=55.0
gl=0.1
gk=9.
gNa=35
#    0  1  2  3  4  5
#Y=[v0,n0,h0,v1,n1,h1]
def rhs(Y,t):
    return[
        I*float(t>50.
↪)+gl*(El-Y[0])+gk*Y[1]**4*(Ek-Y[0])+gNa*minf(Y[0])**3*Y[2]*(ENa-Y[0]),
        (ninf(Y[0])-Y[1])/ntau(Y[0]),
        (hinf(Y[0])-Y[2])/htau(Y[0])
    ]
```

```
def mix_rhs(Y,t):
    return[
        (I*float(t>50.)-0.13+6e-4*(Y[0]+60.6)**2*(55-Y[0])/L2(Y[0],1.,-60.6,1.
↪,0.0)+gk*Y[1]**4*(Ek-Y[0]))/xvtau(Y[0]),
        #(I*float(t>50.)-0.13+6e-4*(Y[0]+60.
↪6)**2*(55-Y[0])+gk*Y[1]**4*(Ek-Y[0])/L2(Y[0],1.,-60.6,1.,0.0))/xvtau(Y[0]),
        (pwl_ninf(Y[0])-Y[1])/pwl_ntau(Y[0]),
    ]

at=np.arange(0, 150.0, 0.01)
asol     = integrate.odeint(rhs,     [30.32243, 0.5407895, 0.1174041], at)
mix_asol = integrate.odeint(mix_rhs, [30.32243, 0.5407895],            at)


figure(3,figsize=(20,15))
subplot2grid((5,5),(0,0))
title("Classical model",fontsize=22)
for Iapp in 0.,0.13,0.2,0.5,2.,7.:
    v0c=[]
    for vm in linspace(-89,40,200):
        n=sp.optimize.fsolve(vfun,0.5,args=(vm,Iapp),xtol=0.01)[0]
        v0c.append((vm,n))
    v0c=array(v0c)
    plot(v0c[:,0],v0c[:,1],"-",label="v-nullcline, I={}".format(Iapp),lw=3)

vm=linspace(-89,40,150)
plot(vm,ninf(vm),"k-",label="n-nullcline",lw=3)
ylim(0,1)
legend(loc=0,fontsize=12)

subplot2grid((5,5),(0,3))
title("Mixed 2D model",fontsize=22)
for Iapp in 0.,0.13,0.2,0.5,2.,7.:
    vm = linspace(-89,40,2000)
    v0m = array(mixV0(vm,Iapp)).T
    plot(v0m[:,0],v0m[:,1],"-",label="v-nullcline,I={}".format(Iapp),lw=3)
vm=linspace(-89,40,150)
plot(vm,pwl_ninf(vm),"k-",label="n-nullcline",lw=3)




subplot2grid((5,5),(1,0),colspan=5,rowspan=4)
ylabel("Voltage (mV)", fontsize=20)
plot(at,    asol[:,0],"k-",lw=3,label="Original model")
plot(at,mix_asol[:,0],"r--",lw=3,label="Mixed 2D model")
```

```
legend(loc=0,fontsize=14)
savefig("2.2-orig-vs-pl-traces.svg")
show()
```

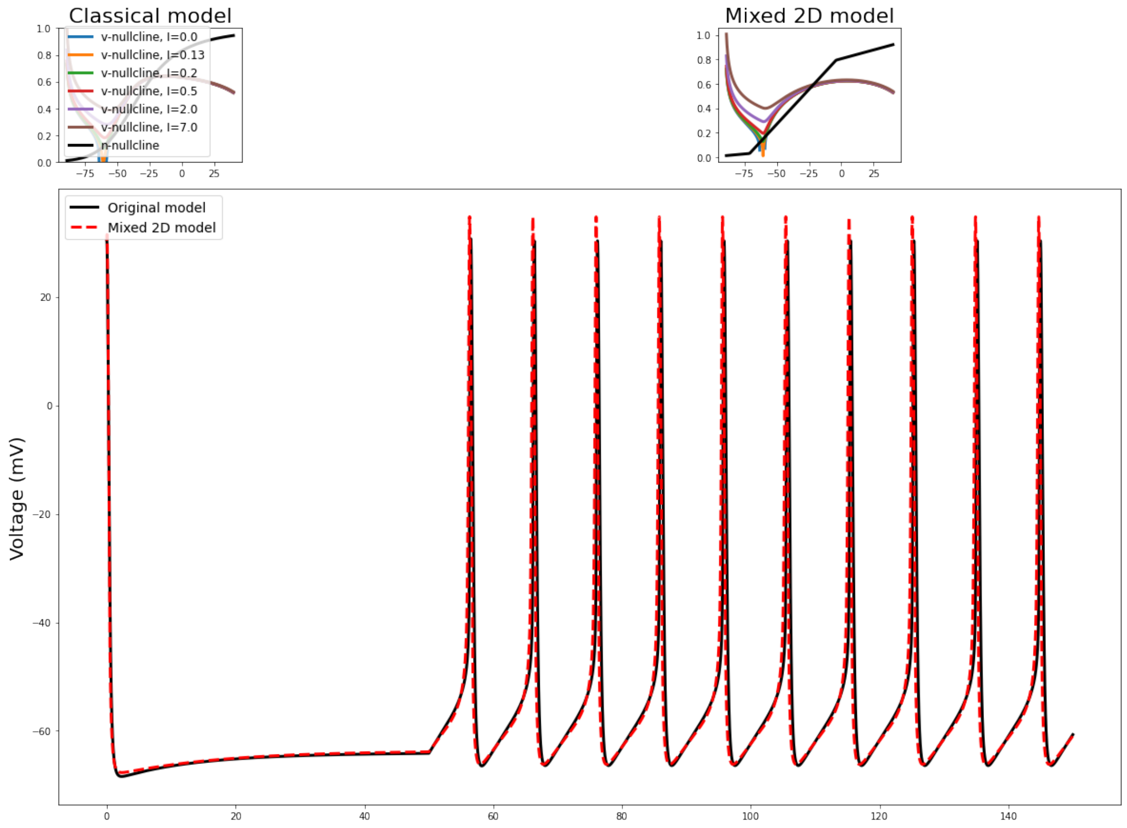

3.2.3 Lookup Table

[29]:

```
tbl_x = array([
    (v,minf(v),hinf(v),ninf(v),htau(v),ntau(v)) for v in linspace(Ek, ENa,201)
])
tbl_stp = tbl_x[1,0]-tbl_x[0,0]
def tbl_rhs(Y,t):
    tbl_idx = int( floor((Y[0]-Ek)/tbl_stp) )
    tbl_v0,tbl_minf0,tbl_hinf0,tbl_ninf0,tbl_htau0,tbl_ntau0 = tbl_x[tbl_idx  ,:
 ↪]
    tbl_v1,tbl_minf1,tbl_hinf1,tbl_ninf1,tbl_htau1,tbl_ntau1 = tbl_x[tbl_idx+1,:
 ↪]
    tbl_ras = (Y[0]-tbl_v0)/tbl_stp
    tbl_minf0 += (tbl_minf1 - tbl_minf0)*tbl_ras
    tbl_hinf0 += (tbl_hinf1 - tbl_hinf0)*tbl_ras
```

```
    tbl_ninf0 += (tbl_ninf1 - tbl_ninf0)*tbl_ras
    tbl_htau0 += (tbl_htau1 - tbl_htau0)*tbl_ras
    tbl_ntau0 += (tbl_ntau1 - tbl_ntau0)*tbl_ras
    return[
        I*float(t>50.
 ↪)+gl*(El-Y[0])+gk*Y[1]**4*(Ek-Y[0])+gNa*tbl_minf0**3*Y[2]*(ENa-Y[0]),
        (tbl_ninf0-Y[1])/tbl_ntau0,
        (tbl_hinf0-Y[2])/tbl_htau0
    ]
at=np.arange(0, 150.0, 0.01)
tbl_asol = integrate.odeint(tbl_rhs, [30.32243, 0.5407895, 0.1174041], ␣
 ↪ at)


figure(3,figsize=(20,12))

ylabel("Voltage (mV)", fontsize=20)
plot(at,    asol[:,0],"k-",lw=3,label="Original model")
plot(at,tbl_asol[:,0],"r--",lw=3,label="Lookup Table")
legend(loc=0,fontsize=14)
savefig("2.1-orig-vs-tbl-traces.svg")
show()
```

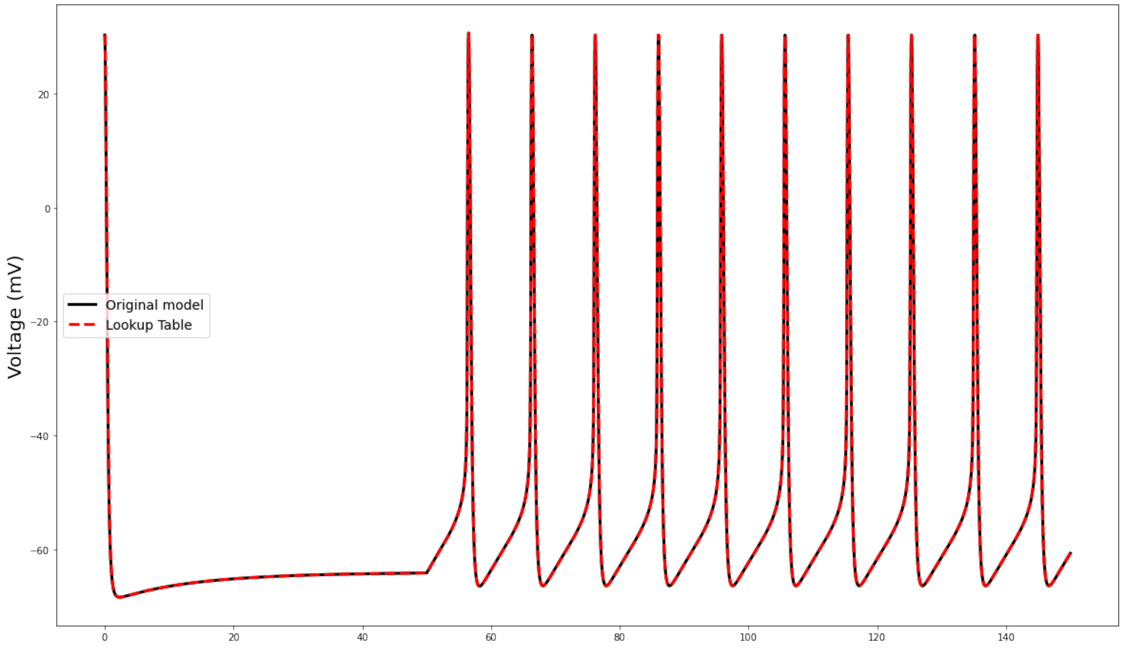

4 Part III: Accuracy Benchmark

4.1 F-I curves

[30]:
```
xvtau = lambda v:0.4+(-38-v)*0.032*float(v<-38)
L2    = lambda x,s0,x0,y0,s1: (y0+s0*(x0-x)) if x<x0 else (y0+s1*(x-x0))

I=4./10000.
El=-65.0
Ek=-90.0
ENa=55.0
gl=0.1
gk=9.
gNa=35
#    0  1  2  3  4  5
#Y=[v0,n0,h0,v1,n1,h1]
def rhs(Y,t):
    return[
        I*(t-20.)*float(t>20.
↪)+gl*(El-Y[0])+gk*Y[1]**4*(Ek-Y[0])+gNa*minf(Y[0])**3*Y[2]*(ENa-Y[0]),
        (ninf(Y[0])-Y[1])/ntau(Y[0]),
        (hinf(Y[0])-Y[2])/htau(Y[0])
    ]


def mix_rhs(Y,t):
    return[
        (I*(t-20.)*float(t>20.)-0.13+6e-4*(Y[0]+60.6)**2*(55-Y[0])/L2(Y[0],1.
↪,-60.6,1.,0.0)+gk*Y[1]**4*(Ek-Y[0]))/xvtau(Y[0]),
        (pwl_ninf(Y[0])-Y[1])/pwl_ntau(Y[0]),
    ]

def pwl_rhs(Y,t):
    return[
        I*(t-20.)*float(t>20.
↪)+gl*(El-Y[0])+gk*Y[1]**4*(Ek-Y[0])+gNa*pwl_minf(Y[0])**3*Y[2]*(ENa-Y[0]),
        (pwl_ninf(Y[0])-Y[1])/pwl_ntau(Y[0]),
        (pwl_hinf(Y[0])-Y[2])/pwl_htau(Y[0])
    ]

def pol_rhs(Y,t):
    return[
        I*(t-20.)*float(t>20.
↪)+gl*(El-Y[0])+gk*Y[1]**4*(Ek-Y[0])+gNa*pol_minf(Y[0])**3*Y[2]*(ENa-Y[0]),
        (pol_ninf(Y[0])-Y[1])/pol_ntau(Y[0]),
```

```
        (pol_hinf(Y[0])-Y[2])/pol_htau(Y[0])
    ]

def tbl_rhs(Y,t):
    #tbl_idx = int( floor((Y[0]-Ek)/tbl_stp) )
    tbl_idx = int( (Y[0]-Ek)/tbl_stp )
    tbl_ras = (Y[0]-tbl_x[tbl_idx  ,0])/tbl_stp
    tbl_cur = tbl_x[tbl_idx  ,1:] + (tbl_x[tbl_idx+1,1:] -tbl_x[tbl_idx  ,1:
↪])*tbl_ras
    return[
        I*(t-20.)*float(t>20.
↪)+gl*(El-Y[0])+gk*Y[1]**4*(Ek-Y[0])+gNa*tbl_cur[0]**3*Y[2]*(ENa-Y[0]),
        (tbl_cur[2]-Y[1])/tbl_cur[4],
        (tbl_cur[1]-Y[2])/tbl_cur[3]
    ]
at=np.arange(0, 10000.0, 0.01)
print("Solving Original model")
asol     = integrate.odeint(rhs,     [30.32243, 0.5407895, 0.1174041], at)
print("Solving piecewise-Linear model")
pwl_asol = integrate.odeint(pwl_rhs, [30.32243, 0.5407895, 0.1174041], at)
print("Solving Polynomial model")
pol_asol = integrate.odeint(pol_rhs, [30.32243, 0.5407895, 0.1174041], at)
print("Solving Mixed 2D model")
mix_asol = integrate.odeint(mix_rhs, [30.32243, 0.5407895],            at)
print("Solving Lookup Table model")
tbl_asol = integrate.odeint(tbl_rhs, [30.32243, 0.5407895, 0.1174041], at)
print("Getting FI curves")
def getFI(rec):
    sp = where(rec[:,0]>10.)[0]
    sp = [ r for l,r in zip(sp[:-1],sp[1:]) if l+1 != r ]
    sp = at[sp]
    return array([ ((0.5*(t1+t2)-20)*I,1000./(t2-t1)) for t1,t2 in zip(sp[:
↪-1],sp[1:]) ])

clsfi = getFI(asol)
mixfi = getFI(mix_asol)
polfi = getFI(pol_asol)
pwlfi = getFI(pwl_asol)
tblfi = getFI(tbl_asol)
print("DONE")
```

```
Solving Original model
Solving piecewise-Linear model
Solving Polynomial model
Solving Mixed 2D model
Solving Lookup Table model
Getting FI curves
```

```
DONE
```

[31]:

```
figure(5,figsize=(9,9))
plot(mixfi[::20,0],mixfi[::20,1],"x",label="Mixed 2D",␣
 ↪ms=15,mfc='#7e0021',mec='#7e0021')
plot(polfi[::20,0],polfi[::20,1],"+",label="Polynomial",␣
 ↪ms=15,mfc='#579d1c',mec='#579d1c')
plot(pwlfi[::20,0],pwlfi[::
 ↪20,1],"d",label="piecewise-Linear",ms=15,mfc='#ffd320',mec='#ffd320')
plot(tblfi[::20,0],tblfi[::20,1],"o",label="Lookup table",␣
 ↪ms=15,mfc='#ff420e',mec='#ff420e')
plot(clsfi[::20,0],clsfi[::20,1],".",label="Original",␣
 ↪ms=20,mfc='#004586',mec='#004586',c='#004586')

ylim(ymin=0)
xlim(xmin=0)
legend(loc=0,fontsize=12)
savefig("2.3-fi-curves.svg")
show()
```

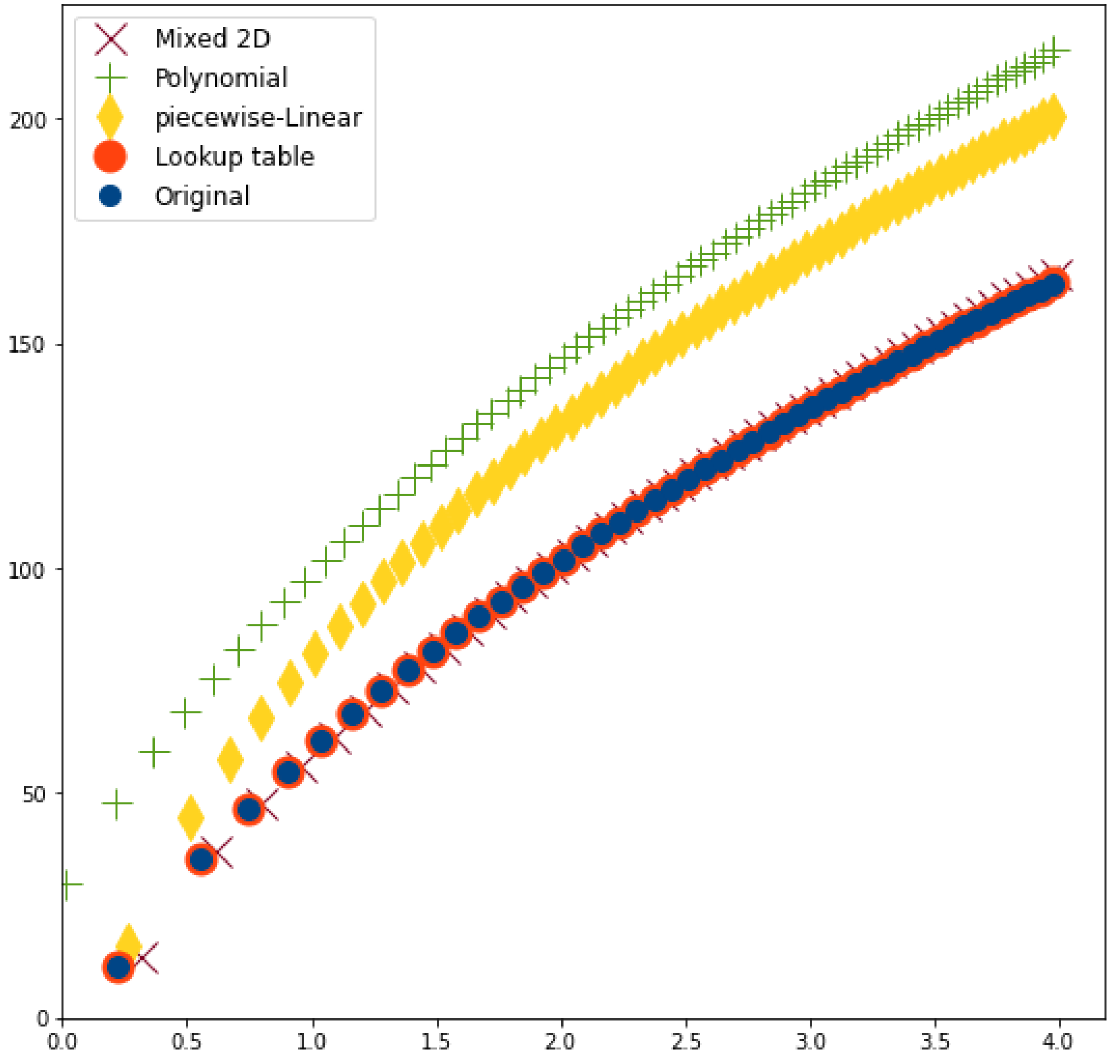

[32]:

```
mixAerr = abs( (interp(clsfi[:,0], mixfi[:,0],mixfi[:,1])-clsfi[:,1]) )
polAerr = abs( (interp(clsfi[:,0], polfi[:,0],polfi[:,1])-clsfi[:,1]) )
pwlAerr = abs( (interp(clsfi[:,0], pwlfi[:,0],pwlfi[:,1])-clsfi[:,1]) )
tblAerr = abs( (interp(clsfi[:,0], tblfi[:,0],tblfi[:,1])-clsfi[:,1]) )

for t,err in zip("Tbl,PL2D,Pol,Pwl".
 ↪split(","),[tblAerr,mixAerr,polAerr,pwlAerr]):
    errmax = argmax(err)
    print(f"The maximal absolute error of {t} approximation:{err[errmax]} Hz")
    print(f"The maximal relative error of {t} approximation:{err[errmax]/
 ↪clsfi[errmax,1]} ")
    print(f"The maximal range    error of {t} approximation:{err[errmax]/1.5}␣
 ↪%")
    print()
```

```
The maximal absolute error of Tbl approximation:0.2683051166180235 Hz
The maximal relative error of Tbl approximation:0.0016393442625362797
The maximal range    error of Tbl approximation:0.178870077745349 %

The maximal absolute error of PL2D approximation:4.88657517734892 Hz
The maximal relative error of PL2D approximation:0.2425207260518269
The maximal range    error of PL2D approximation:3.25771678489928 %

The maximal absolute error of Pol approximation:52.120395580391346 Hz
The maximal relative error of Pol approximation:0.32001922886357254
The maximal range    error of Pol approximation:34.746930386927566 %

The maximal absolute error of Pwl approximation:37.085772992998756 Hz
The maximal relative error of Pwl approximation:0.22993179255661927
The maximal range    error of Pwl approximation:24.72384866199917 %
```
