# Supplementary material for: Polynomial, piecewise-Linear, Step (PLS): A Simple, Scalable, and Efficient Framework for Modeling Neurons
Source: Front Neuroinform. 2021 May 6;15:642933. doi: 10.3389/fninf.2021.642933 (PMC8134741; doi:10.3389/fninf.2021.642933)
Supplement: Supplementary Data Sheet 2 — Listing of Python 3 Jupyther-notebook page with two phenomenological models described in section 4. [file Data_Sheet_2.PDF]

PLS-Integrator

March 1, 2021

1 Integrating neuron (Type 1)

1.1 without adaptaion

$$
\begin{aligned}
L_1(v,v_3,r_0,r_1,0) \quad \dot{v} &= P_3(v,v_0,v_1,v_2)L_1(v,v_0,a_0,a_1,0)+I-w^2 \\
S_2(v,v_6,,v_7,s_0,s_1,s_2) \quad \dot{w} &= L_2(v,v_4,0,v_5,1,0,0)-w
\end{aligned}
$$

1.1.1 with saddale-node depolarization block

[5]:
```
%matplotlib inline
from numpy import *
from matplotlib.pyplot import *
from scipy.integrate import odeint
import sympy
from pls import *
v0,v1,v2 = -65.,-45., 55.
a0,a1     = 3.5e-6,-1e-4 #a2 = 0.
vmin,vmax= -55.45176241886681, 18.78509575220014
I0        = -a0*(vmin-v0)*(vmin-v1)*(v2-vmin)
I1        = 1.-a0*(vmax-v0)*(vmax-v1)*(v2-vmax)
#--- \tau_v
v3        = -35.
r0,r1     = 0.04, -0.004 #r2 = 0.
#--- n_\infty
v4,v5     = -40.,-5.
#--- \tau_n
v6,v7     = vmin,vmax
s0,s1,s2 = 5.,7.6,1.8
print(I0,I1)
```

```
0.038579219773071036 0.32260596541211417
```

[6]:
```
def safesqrt(x,y,limit=None):
    if limit is None:
        return column_stack((x[where(y>=0.)],sqrt(y[where(y>=0.)])))
    else:
        z = safesqrt(x,y)
        return z[where(z[:,1]<limit)]
```

```
vm      = linspace(-70,55,1251)
v0c    = safesqrt(vm, vectorize(P3)(vm,v0,v1,v2)*vectorize(L1)(vm,v0,a0,a1,0.)+0.
 ↪ ,1)
v0cI0 = safesqrt(vm, vectorize(P3)(vm,v0,v1,v2)*vectorize(L1)(vm,v0,a0,a1,0.
 ↪)+I0 ,1)
v0cI05= safesqrt(vm, vectorize(P3)(vm,v0,v1,v2)*vectorize(L1)(vm,v0,a0,a1,0.
 ↪)+(I0+I1)/2 ,1)
v0cI1 = safesqrt(vm, vectorize(P3)(vm,v0,v1,v2)*vectorize(L1)(vm,v0,a0,a1,0.
 ↪)+I1 ,1)

f1=figure(1,figsize=(12,6))
subplot(121)
plot(v0c[:,0],v0c[:,1])
plot(v0cI0[:,0], v0cI0[:,1],label='{:0.2g}'.format(I0))
plot(v0cI05[:,0],v0cI05[:,1],label='{:0.2g}'.format((I0+I1)/2))
plot(v0cI1[:,0], v0cI1[:,1],label='{:0.2g}'.format(I1))
plot(vm,vectorize(L2)(vm,v4,0.,v5,1.,0.,0.),"k-")
legend(loc=0)

subplot(122)
plot(vm,vectorize(L1)(vm,v3,r0,r1,0.))
plot(vm,vectorize(S2)(vm,v6,v7,s0,s1,s2),"k-")
f1.savefig("pls-t1-nulls-saddlenode-depblk.svg")
f1.savefig("pls-t1-nulls-saddlenode-depblk.png")
show()
```

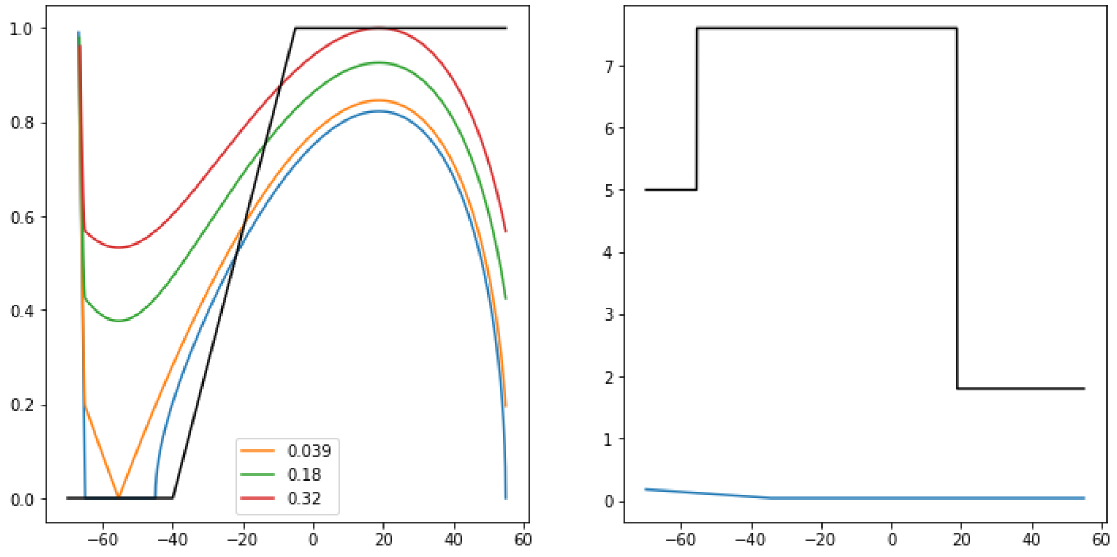

[7]:
```
symV, symW, symI = sympy.symbols('v w I')
```

```
def getroots(I):
    vm = linspace(v0-1.,v2+1.,int(v2-v0)*100+102)
    vnull = vectorize(P3)(vm,v0,v1,v2)*vectorize(L1)(vm,v0,a0,a1,0.)+I
    wnull = vectorize(L2)(vm,v4,0.,v5,1.,0.,0.)
    vnull = sqrt(vnull)
    idx = where((vnull-wnull)**2<1e-3)[0]
    if   len(idx) > 2:
        idx = [idx[0]]+[l2 for l1,l2 in zip(idx[:-1],idx[1:]) if l1+1 != l2]
    return vm[idx],vectorize(L2)(vm[idx],v4,0.,v5,1.,0.,0.)

def getJacobian(v,w,I):
    vrhs = (symP3(symV,v,v0,v1,v2)*symL1(symV,v,v0,a0,a1,0.)+symI-symW**2)/
↪symL1(symV,v,v3,r0,r1,0.)
    wrhs = (symL2(symV,v,v4,0.,v5,1.,0.,0.)-w)/symS2(symV,v,v6,v7,s0,s1,s2)
    dvdv, dvdw = vrhs.diff(symV),vrhs.diff(symW)
    dwdv, dwdw = wrhs.diff(symV),wrhs.diff(symW)
    return array([
        [dvdv.subs(symV,v).subs(symW,w).subs(symI,I),dvdw.subs(symV,v).
↪subs(symW,w).subs(symI,I)],
        [dwdv.subs(symV,v).subs(symW,w).subs(symI,I),dwdw.subs(symV,v).
↪subs(symW,w).subs(symI,I)]
    ])

def getStability(I,verb=False):
    vx,wx = getroots(I)
    ret = []
    for v,w in zip(vx,wx):
        #print("vwi",v,w,I)
        m = getJacobian(v,w,I)
        #print("m",m.tolist())
        if verb:
            print("=== FIX POINT ===")
            print(" > V*={:0.2f} W*={:0.2f}".format(v,w))
            #print(" > tau={:0.2f} det={:0.2f}".format(m[0,0]+m[1,1],␣
↪m[0,0]*m[1,1]-m[1,0]*m[0,1]))
        ev, eV = linalg.eig(m.astype(complex))
        if verb:
            print(ev)
            print(eV)
        ret.append( (v,w,ev,eV))
    return ret
print( getStability(0.) )
```

```
[(-65.04222791504834, 0.0, array([-0.17959872+0.j,  0.        +0.j]),
array([[1.+0.j, 0.+0.j],
       [0.+0.j, 1.+0.j]])), (-44.999586811007354, 0.0, array([0.08750651+0.j, 0.
+0.j]), array([[1.+0.j, 0.+0.j],
```

```
       [0.+0.j, 1.+0.j]])), (-24.05966449053797, 0.45543815741320093,
array([0.17652953+0.23333667j, 0.17652953-0.23333667j]), array([[0.99991747+0.j
, 0.99991747+0.j        ],
       [0.00775143-0.01024584j, 0.00775143+0.01024584j]]))]
```

```
<ipython-input-7-14e363a02e63>:7: RuntimeWarning: invalid value encountered in
sqrt
  vnull = sqrt(vnull)
```

[8]:

```
def getCurr(t):
    if          t <  500: return I0
    elif 500 <= t < 9000: return I0*1.01
    else                : return I1*(0.9+0.2*(t-9000)/1000)
def t1rhs(Y,t):
    v,w = Y
    return[
        (P3(v,v0,v1,v2)*L1(v,v0,a0,a1,0.)+getCurr(t)-w**2)/L1(v,v3,r0,r1,0.),
        (L2(v,v4,0.,v5,1.,0.,0.)-w)/S2(v,v6,v7,s0,s1,s2)
    ]
I = I1/(10000-500)
at = arange(0,10000,0.01)
res = odeint(t1rhs,[vmin,0],at)
```

[9]:

```
f2 = figure(2, figsize=(12,18))
subplot2grid((4,2),(0,0),colspan=2)
plot(at,res[:,0])
subplot2grid((4,2),(1,0),colspan=2)
plot(at,vectorize(getCurr)(at))
plot([500.,9000.],[I0,I1],'o')

subplot2grid((4,2),(2,0))
plot(at[:200000],res[:200000,0])
subplot2grid((4,2),(2,1))
plot(at[-200000:],res[-200000:,0])
subplot2grid((4,2),(3,0))
plot(v0c[:,0],v0c[:,1])
plot(v0cI0[:,0],v0cI0[:,1])
plot(vm,vectorize(L2)(vm,v4,0.,v5,1.,0.,0.),"k-")
plot(res[:200000,0],res[:200000,1])
subplot2grid((4,2),(3,1))
plot(v0c[:,0],v0c[:,1])
plot(v0cI1[:,0],v0cI1[:,1])
plot(vm,vectorize(L2)(vm,v4,0.,v5,1.,0.,0.),"k-")
plot(res[-200000:,0],res[-200000:,1])
f2.savefig("pls-t1-ramp-saddlenode-depblk.svg")
f2.savefig("pls-t1-ramp-saddlenode-depblk.png")
show()
```

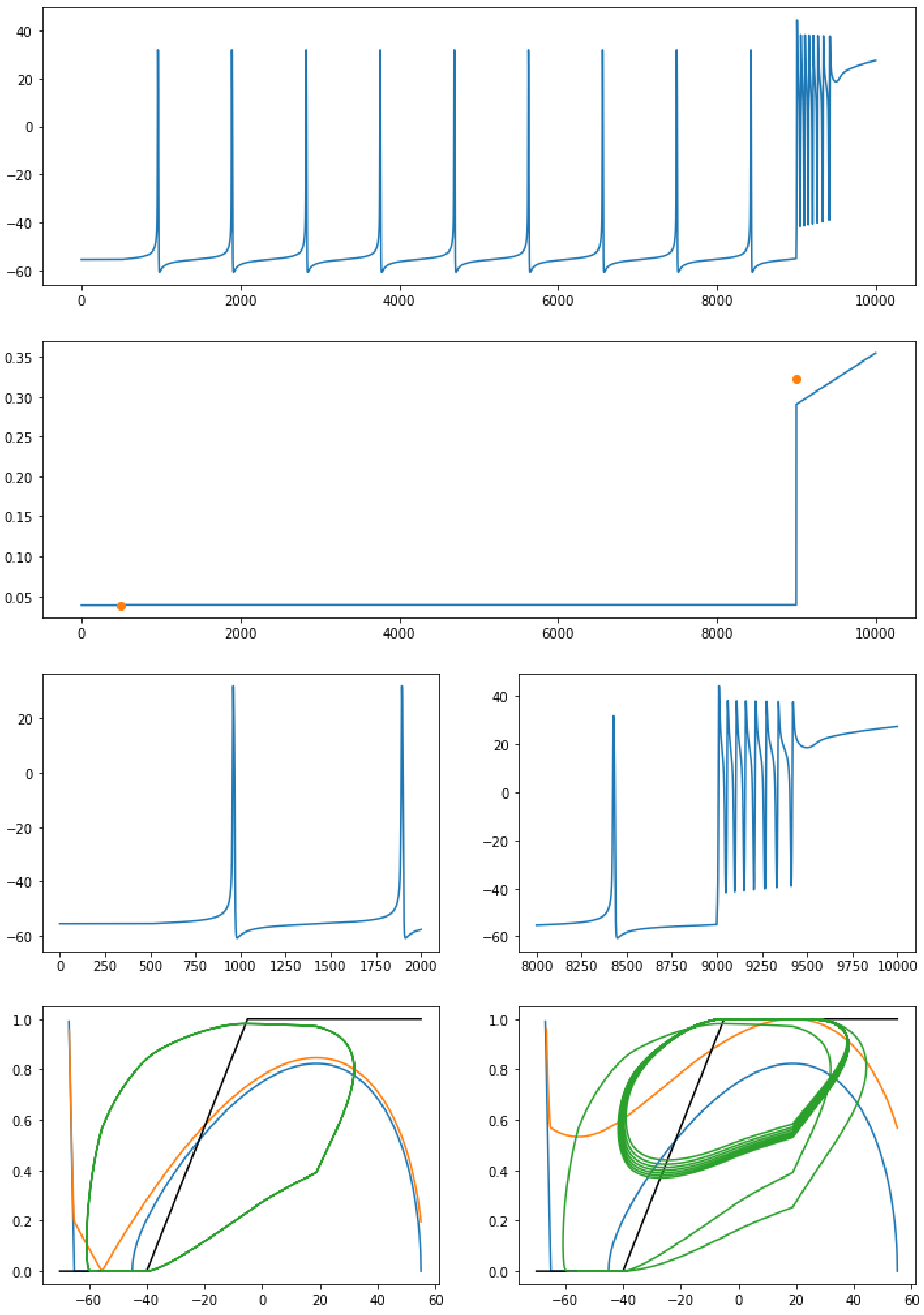
40
20
0
−20
−40
−60
0
2000
4000
6000
8000
10000
0.35
0.30
0.25
0.20
0.15
0.10
0.05
0
250
500
750
1000
1250
1500
1750
2000
8000
8250
8500
8750
9000
9250
9500
9750
10000
1.0
0.8
0.6
0.4
0.2
0.0
−60
−40
−20
0
20
40
60

1.1.2 with andronove-hopf depolarization block

[10]:
```
v4,v5    = vmin+2.5,vmax+2.5
```

[11]:
```
I   = I0*0.9
ret = getStability(I)
while len(ret) != 1:
    I += I0*0.05
    ret = getStability(I)


fv,fw,fev,feV = ret[0]

while not (fev[0].real <= 0. and fev[1].real <= 0.):
    Ic = I
    I += I0*0.01
    ret = getStability(I)
    fv,fw,fev,feV = ret[0]

print(Ic,I,fev)
I2=(Ic+I)/2
#I2=I2*(3500-50)/(10000-500)
```

```
<ipython-input-7-14e363a02e63>:7: RuntimeWarning: invalid value encountered in
sqrt
  vnull = sqrt(vnull)
```

```
0.31827856312784014 0.31866435532557086 [-0.00012688+0.60136721j
-0.00012688-0.60136721j]
```

[12]:
```
v0c   = safesqrt(vm, vectorize(P3)(vm,v0,v1,v2)*vectorize(L1)(vm,v0,a0,a1,0.)+0.
 ↪ ,1)
v0cI0 = safesqrt(vm, vectorize(P3)(vm,v0,v1,v2)*vectorize(L1)(vm,v0,a0,a1,0.
 ↪)+I0 ,1)
v0cI1 = safesqrt(vm, vectorize(P3)(vm,v0,v1,v2)*vectorize(L1)(vm,v0,a0,a1,0.
 ↪)+I2 ,1)

f1=figure(1,figsize=(12,6))
subplot(121)
plot(v0c[:,0],v0c[:,1])
plot(v0cI0[:,0],v0cI0[:,1])
plot(v0cI1[:,0],v0cI1[:,1])
plot(vm,vectorize(L2)(vm,v4,0.,v5,1.,0.,0.),"k-")

subplot(122)
plot(vm,vectorize(L1)(vm,v3,r0,r1,0.))
plot(vm,vectorize(S2)(vm,v6,v7,s0,s1,s2),"k-")
show()
```

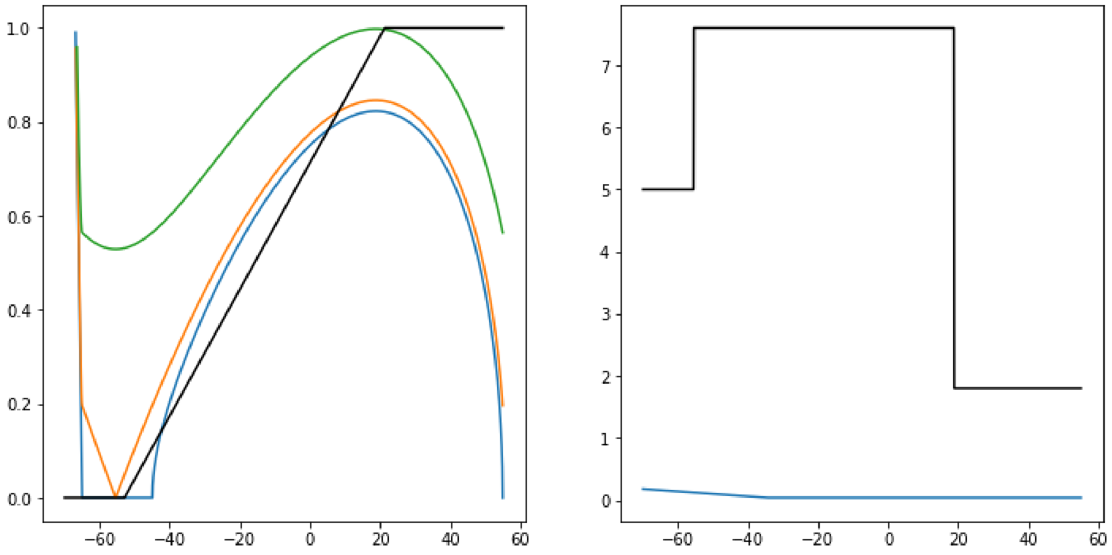

[13]:
```
def t1rhs(Y,t):
    v,w = Y
    return[
        (P3(v,v0,v1,v2)*L1(v,v0,a0,a1,0.)+I*(t-50)-w**2)/L1(v,v3,r0,r1,0.),
        (L2(v,v4,0.,v5,1.,0.,0.)-w)/S2(v,v6,v7,s0,s1,s2)
    ]
I = I2/(10000-500)
at = arange(0,10000,0.01)
res = odeint(t1rhs,[v0,0],at)
```

[14]:
```
f2 = figure(2, figsize=(12,18))
subplot2grid((3,2),(0,0),colspan=2)
plot(at,res[:,0])
subplot2grid((3,2),(1,0))
plot(at[200000:450000],  res[200000:450000,0])
subplot2grid((3,2),(1,1))
plot(at[-200000:],res[-200000:,0])
subplot2grid((3,2),(2,0))
plot(v0c[:,0],v0c[:,1])
plot(v0cI0[:,0],v0cI0[:,1])
plot(vm,vectorize(L2)(vm,v4,0.,v5,1.,0.,0.),"k-")
plot(res[:400000,0],res[:400000,1])
subplot2grid((3,2),(2,1))
plot(v0c[:,0],v0c[:,1])
plot(v0cI1[:,0],v0cI1[:,1])
plot(vm,vectorize(L2)(vm,v4,0.,v5,1.,0.,0.),"k-")
plot(res[-200000:,0],res[-200000:,1])
```

```
show()
```

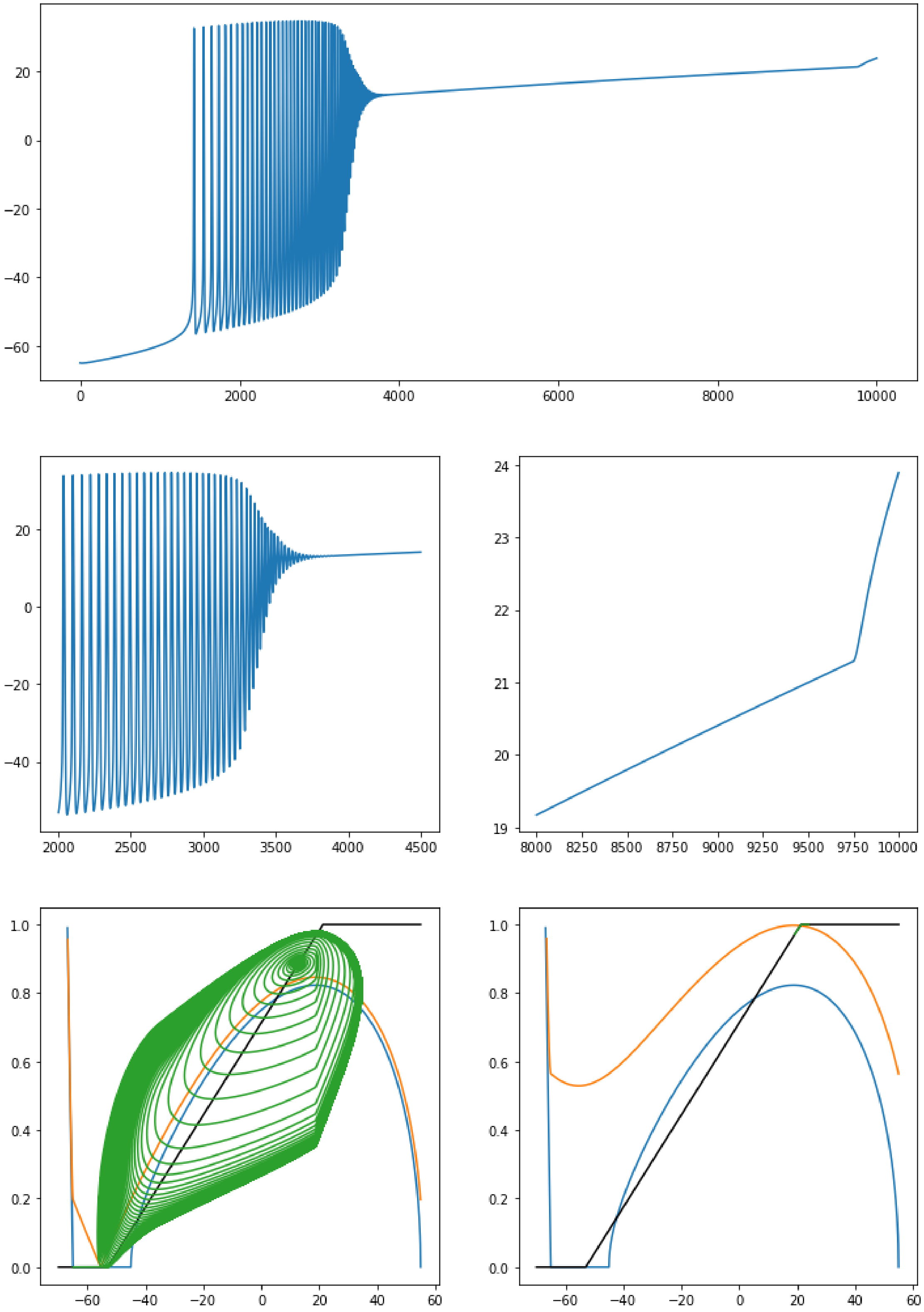

20
0
−20
−40
−60
0 2000 4000 6000 8000 10000
20
0
−20
−40
2000 2500 3000 3500 4000 4500
24
23
22
21
20
19
8000 8250 8500 8750 9000 9250 9500 9750 10000
1.0
0.8
0.6
0.4
0.2
0.0
−60 −40 −20 0 20 40 60
1.0
0.8
0.6
0.4
0.2
0.0
−60 −40 −20 0 20 40 60

1.2 with linear adaptaion

$$
\begin{aligned}
L_1(v, v_3, r_0, r_1, 0) \quad \dot{v} &= P_3(v, v_0, v_1, v_2) L_1(v, v_0, a_0, a_1, 0) + I - w^2 - z \\
S_2(v, v_6, , v_7, s_0, s_1, s_2) \quad \dot{w} &= L_2(v, v_4, 0, v_5, 1, 0, 0) - w \\
q_0 \quad \dot{z} &= L_2(v, v_8, z_0, v_9, z_1, 0, 0) - z
\end{aligned}
$$

[15]:
```
v4,v5     = -40.,-5.
#--- z_\infty
v8,v9     = -35.,10.
z0,z1     = 0., 2.
#--- \tua_z
q0        = 500
```

[16]:
```
def t1rhs(Y,t):
    v,w,z = Y
    return[
        (P3(v,v0,v1,v2)*L1(v,v0,a0,a1,0.)+I1-w**2-z)/L1(v,v3,r0,r1,0.),
        (L2(v,v4,0.,v5,1.,0.,0.)-w)/S2(v,v6,v7,s0,s1,s2),
        (L2(v,v8,z0,v9,z1,0.,0.)-z)/q0
    ]

at = arange(0,2000,0.01)
res = odeint(t1rhs,[v0,0,0],at)
```

[17]:
```
v0c   = safesqrt(vm, vectorize(P3)(vm,v0,v1,v2)*vectorize(L1)(vm,v0,a0,a1,0.)+0.
 ↪ ,1)
v0cI0 = safesqrt(vm, vectorize(P3)(vm,v0,v1,v2)*vectorize(L1)(vm,v0,a0,a1,0.
 ↪)+I1 ,1)
v0cI1 = safesqrt(vm, vectorize(P3)(vm,v0,v1,v2)*vectorize(L1)(vm,v0,a0,a1,0.
 ↪)+I1-amax(res[:,2]) ,1)



f2 = figure(2, figsize=(12,18))
subplot2grid((3,2),(0,0),colspan=2)
plot(at,res[:,0])
subplot2grid((3,2),(1,0))
plot(at,res[:,1])
subplot2grid((3,2),(1,1))
plot(at,res[:,2])
subplot2grid((3,2),(2,0))
plot(v0c[:,0],v0c[:,1])
plot(v0cI0[:,0],v0cI0[:,1])
plot(vm,vectorize(L2)(vm,v4,0.,v5,1.,0.,0.),"k-")
plot(res[:200000,0],res[:200000,1])
```

```
subplot2grid((3,2),(2,1))
plot(v0c[:,0],v0c[:,1])
plot(v0cI1[:,0],v0cI1[:,1])
plot(vm,vectorize(L2)(vm,v4,0.,v5,1.,0.,0.),"k-")
plot(res[-200000:,0],res[-200000:,1])

show()
```

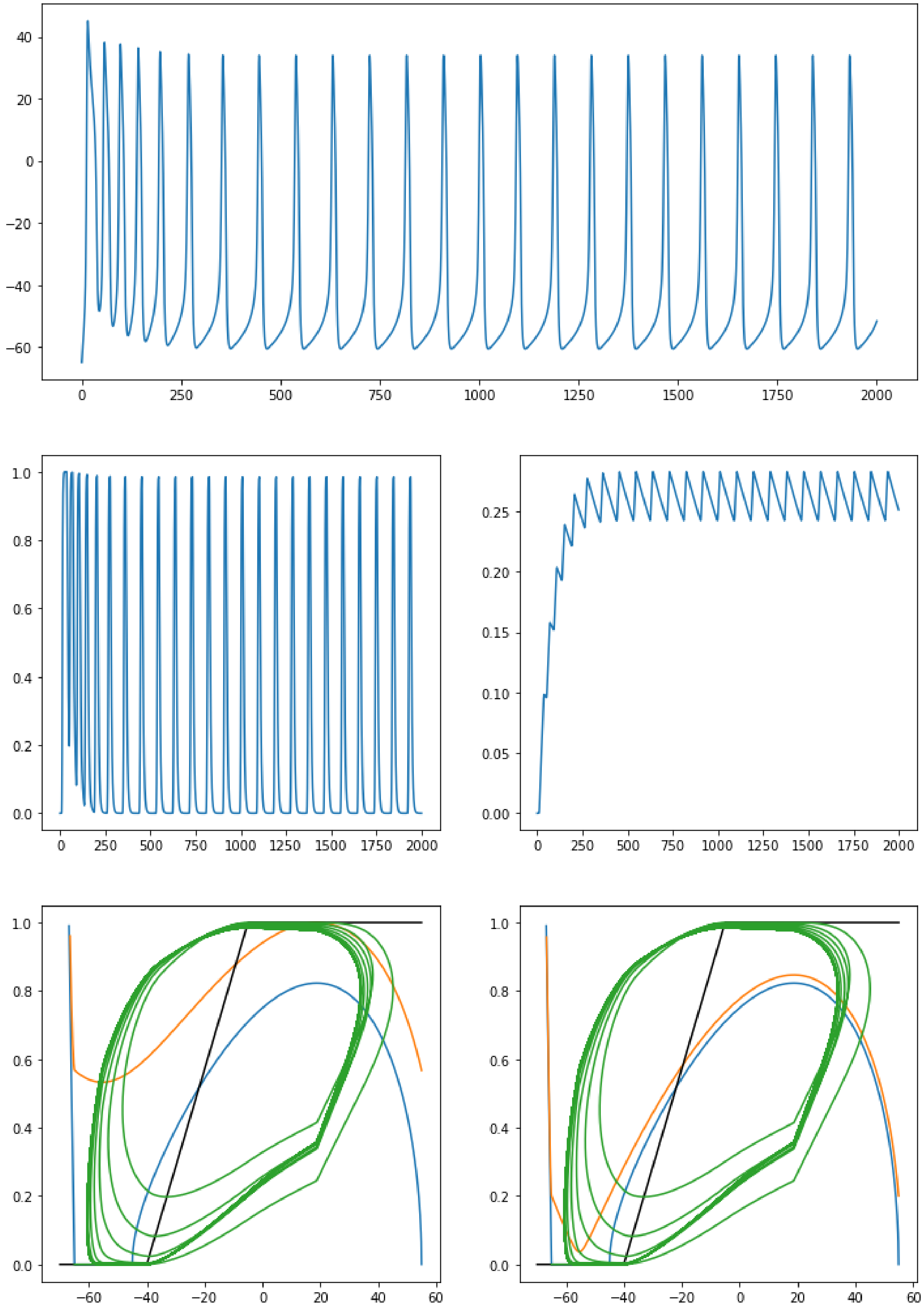
40
20
0
−20
−40
−60
0
250
500
750
1000
1250
1500
1750
2000
1.0
0.8
0.6
0.4
0.2
0.0
0.25
0.20
0.15
0.10
0.05
0.00
−60
−40
−20
20
40
60

1.3 with non-linear adaptaion

$$\begin{aligned} L_1(v,v_3,r_0,r_1,0) \quad \dot{v} &= P_3(v,v_0+z,v1+z,v_2)L_1(v,v_0+z,a_0,a_1,0)+I-w^2 \\ S_2(v,v_6,,v_7,s_0,s_1,s_2) \quad \dot{w} &= L_2(v,v_4,0,v_5,1,0,0)-w \\ q_0 \quad \dot{z} &= L_2(v,v_8,v_1,v_9,v_1+\Delta,0,0)-z \end{aligned}$$

[18]:
```
v4,v5     = -40.,-5.
#--- z_\infty
v8,v9     = -35.,10.
Delta     = 100
z0,z1     = 0,Delta
#--- \tua_z
q0        = 250
```

[19]:
```
def t1rhs(Y,t):
    v,w,z = Y
    return[
        (P3(v,v0+z,v1+z,v2)*L1(v,v0+z,a0,a1,0.)+I1-w**2)/L1(v,v3,r0,r1,0.),
        (L2(v,v4,0.,v5,1.,0.,0.)-w)/S2(v,v6,v7,s0,s1,s2),
        (L2(v,v8,z0,v9,z1,0.,0.)-z)/q0
    ]

at = arange(0,2000,0.01)
res = odeint(t1rhs,[v0,0,0.],at)
```

[20]:
```
v0c   = safesqrt(vm, vectorize(P3)(vm,v0,v1,v2)*vectorize(L1)(vm,v0,a0,a1,0.)+0.
 ↪ ,1)
v0cI0 = safesqrt(vm, vectorize(P3)(vm,v0,v1,v2)*vectorize(L1)(vm,v0,a0,a1,0.
 ↪)+I1 ,1)
zm    = amax(res[:,2])
v0cI1 = safesqrt(vm,␣
 ↪vectorize(P3)(vm,v0+zm,v1+zm,v2)*vectorize(L1)(vm,v0+zm,a0,a1,0.)+I1 ,1)


f2 = figure(2, figsize=(12,18))
subplot2grid((3,2),(0,0),colspan=2)
plot(at,res[:,0])
subplot2grid((3,2),(1,0))
plot(at,res[:,1])
subplot2grid((3,2),(1,1))
plot(at,res[:,2])
subplot2grid((3,2),(2,0))
plot(v0c[:,0],v0c[:,1])
plot(v0cI0[:,0],v0cI0[:,1])
plot(vm,vectorize(L2)(vm,v4,0.,v5,1.,0.,0.),"k-")
plot(res[:200000,0],res[:200000,1])
subplot2grid((3,2),(2,1))
```

```
plot(v0c[:,0],v0c[:,1])
plot(v0cI1[:,0],v0cI1[:,1])
plot(vm,vectorize(L2)(vm,v4,0.,v5,1.,0.,0.),"k-")
plot(res[-200000:,0],res[-200000:,1])

show()
```

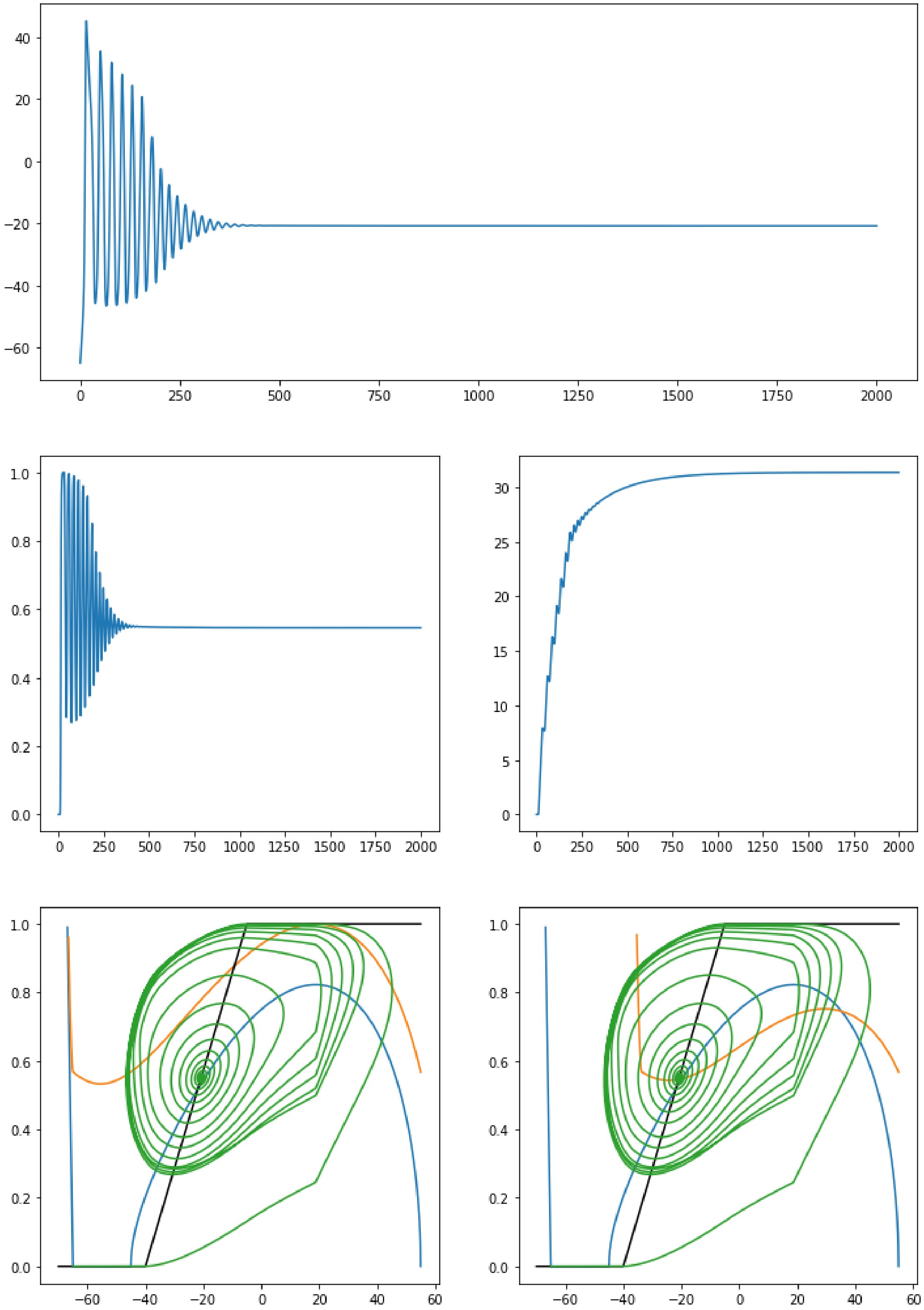

40 20 0 −20 −40 −60
0 250 500 750 1000 1250 1500 1750 2000
1.0 0.8 0.6 0.4 0.2 0.0
0 250 500 750 1000 1250 1500 1750 2000
30 25 20 15 10 5 0
0 250 500 750 1000 1250 1500 1750 2000
1.0 0.8 0.6 0.4 0.2 0.0
−60 −40 −20 0 20 40 60
1.0 0.8 0.6 0.4 0.2 0.0
−60 −40 −20 0 20 40 60

PLS-Resonator

March 1, 2021

1 Resonant neuron (Type 2)

1.1 without adaptaion saddle-node depolarization block

$$\begin{aligned} L_1(v, v_3, r_0, r_1, 0) \quad \dot{v} &= P_{32}(v, v_0, v_2) L_1(v, v_0, a_0, a_1, 0) + I - w^2 \\ S_2(v, v_6, , v_7, s_0, s_1, s_2) \quad \dot{w} &= L_2(v, v_4, 0, v_5, 1, 0, 0) - w \end{aligned}$$

[1]:
```
%matplotlib inline
from numpy import *
from matplotlib.pyplot import *
from scipy.integrate import odeint
import sympy
from pls import *
v0,v2 = -65., 55.
a0,a1    = 3.25e-6,-1e-4 #a2 = 0.
#--- \tau_v
v3       = -35.
r0,r1    = 0.04, -0.004 #r2 = 0.
#--- n_\infty
v4,v5    = -75.,-5.
#--- \tau_n
v6,v7    = -55.5,18.
s0,s1,s2 = 5.,7.6,1.8
wp       = 2
```

[2]:
```
symV, symW, symI = sympy.symbols('v w I')

def getroots(I):
    vm    = linspace(v4-1.,v2+1.,int(v2-v4)*1000+1002)
    vnull = vectorize(P32)(vm,v0,v2)*vectorize(L1)(vm,v0,a0,a1,0.)+I
    wnull = vectorize(L2)(vm,v4,0.,v5,1.,0.,0.)
    if   wp == 4: vnull = sqrt(sqrt(vnull))
    elif wp == 2: vnull = sqrt(     vnull )
    numidx= where(~isnan(vnull))[0]
    nDel  = vnull[numidx]-wnull[numidx]
    idx = where((nDel[:-1]>0.)*(nDel[1:]<=0.)+(nDel[:-1]<=0.)*(nDel[1:]>=0.))[0]
    if   len(idx) > 2:
        idx = [idx[0]]+[l2 for l1,l2 in zip(idx[:-1],idx[1:]) if l1+1 != l2]
```

```
    elif len(idx) == 0:
        #DB>>
        raise Exception("empty index")
        #<<DB
    return vm[numidx[idx]],vectorize(L2)(vm[numidx[idx]],v4,0.,v5,1.,0.,0.)

def getJacobian(v,w,I):
    #vrhs = (symP32(symV,v,v0,v2)*symL1(symV,v,v0,a0,a1,0.)+symI-symW**4)/
↪symL1(symV,v,v3,r0,r1,0.)
    vrhs = (symP32(symV,v,v0,v2)*symL1(symV,v,v0,a0,a1,0.)+symI-symW**wp    )/
↪symL1(symV,v,v3,r0,r1,0.)
    wrhs = (symL2(symV,v,v4,0.,v5,1.,0.,0.)-symW)/symS2(symV,v,v6,v7,s0,s1,s2)
    dvdv, dvdw = vrhs.diff(symV),vrhs.diff(symW)
    dwdv, dwdw = wrhs.diff(symV),wrhs.diff(symW)
    return array([
        [dvdv.subs(symV,v).subs(symW,w).subs(symI,I),dvdw.subs(symV,v).
↪subs(symW,w).subs(symI,I)],
        [dwdv.subs(symV,v).subs(symW,w).subs(symI,I),dwdw.subs(symV,v).
↪subs(symW,w).subs(symI,I)]
    ])

def getStability(I,verb=False):
    vx,wx = getroots(I)
    ret = []
    for v,w in zip(vx,wx):
        #print("vwi",v,w,I)
        m = getJacobian(v,w,I)
        #print("m",m.tolist())
        if verb:
            print("=== FIX POINT ===")
            print(" > V*={:0.2f} W*={:0.2f}".format(v,w))
            #print(" > tau={:0.2f} det={:0.2f}".format(m[0,0]+m[1,1],␣
↪m[0,0]*m[1,1]-m[1,0]*m[0,1]))
        ev, eV = linalg.eig(m.astype(complex))
        if verb:
            print(ev)
            print(eV)
        ret.append( (v,w,ev,eV))
    return ret
i,ic = 0.,0.
ret = getStability(i)
for pwr in range(-1,-9,-1):
    ibase = ic
    for n in range(0,10):
        ic = i
        i  = ibase + n*10**pwr
        ret = getStability(i)
```

```
        if len(ret) != 1:
            print(ret)
            raise Exception(f"Cannot detect a single fixed point at {i}")
        v,w,ev,eV = ret[0]
        if ev[0].real > 0.: break
    else:
        ic = ibase + 9*10**pwr
        i  = ic

I0=ic
ic=i
for pwr in range(-1,-9,-1):
    ibase = ic
    for n in range(0,10):
        ic = i
        i  = ibase + n*10**pwr
        ret = getStability(i)

        if len(ret) != 1:  break
        v,w,ev,eV = ret[0]
        if ev[0].real < 0.: break
    else:
        ic = ibase + 9*10**pwr
        i  = ic
I1=(i+ic)/2
print(f"I0={I0}, I1={I1}")
```

```
<ipython-input-2-bb42a5087c7d>:8: RuntimeWarning: invalid value encountered in
sqrt
  elif wp == 2: vnull = sqrt(     vnull )
```

```
I0=0.05299323, I1=0.168000005
```

[3]:
```
vm = linspace(-90,60,6001)
I0 = 0.05
def safesqrt(x,y,limit=None):
    if limit is None:
        if   wp == 4:
            return column_stack((x[where(y>=0.)],sqrt(sqrt(y[where(y>=0.)]))))
        elif wp == 2:
            return column_stack((x[where(y>=0.)],     sqrt(y[where(y>=0.)] )))
        else:
            return column_stack((x[where(y>=0.)],          y[where(y>=0.)]  ))
    else:
        z = safesqrt(x,y)
        return z[where(z[:,1]<limit)]
```

```
v0c    = safesqrt(vm, vectorize(P32)(vm,v0,v2)*vectorize(L1)(vm,v0,a0,a1,0.)+0.␣
 ↪,1)
v0cI0  = safesqrt(vm, vectorize(P32)(vm,v0,v2)*vectorize(L1)(vm,v0,a0,a1,0.)+I0␣
 ↪,1)
v0cI1  = safesqrt(vm, vectorize(P32)(vm,v0,v2)*vectorize(L1)(vm,v0,a0,a1,0.)+I1␣
 ↪,1)

f1=figure(1,figsize=(12,6))
subplot(121)
plot(v0c[:,0],v0c[:,1])
#for i in arange(0.,0.05,0.01).tolist()+[0.1,0.3,0.6]:
#    xV = safesqrt(vm, vectorize(P32)(vm,v0,v2)*vectorize(L1)(vm,v0,a0,a1,0.)+i␣
 ↪,1)
#    plot(xV[:,0], xV[:,1],label='{:0.2g}'.format(i))
plot(v0cI0[:,0], v0cI0[:,1],label='{:0.2g}'.format(I0))
plot(v0cI1[:,0], v0cI1[:,1],label='{:0.2g}'.format(I1))
plot(vm,vectorize(L2)(vm,v4,0.,v5,1.,0.,0.),"k-")
legend(loc=0)
#xlim(-55,-35)
#ylim(0.2,0.6)

subplot(122)
plot(vm,vectorize(L1)(vm,v3,r0,r1,0.))
plot(vm,vectorize(S2)(vm,v6,v7,s0,s1,s2),"k-")
f1.savefig("pls-t2-nulls-saddlenode-depblk.svg")
f1.savefig("pls-t2-nulls-saddlenode-depblk.png")
show()
```

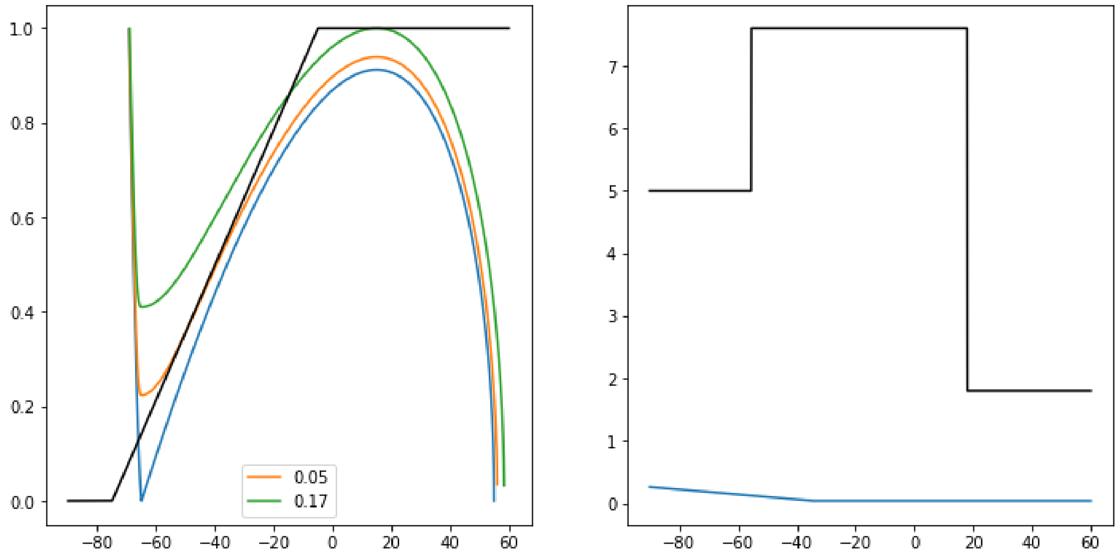

[4]:
```
def getCurr(t):
    if          t <  500: return I0
    elif 500 <= t < 9000: return I0*1.057
    else                : return I1*(0.9+0.2*(t-9000)/1000)
def t2rhs(Y,t):
    v,w = Y
    return[
        (P32(v,v0,v2)*L1(v,v0,a0,a1,0.)+getCurr(t)-w**wp)/L1(v,v3,r0,r1,0.),
        (L2(v,v4,0.,v5,1.,0.,0.)-w)/S2(v,v6,v7,s0,s1,s2)
    ]
I = (I1-I0)/(10000-250)
at = arange(0,10000,0.01)
res = odeint(t2rhs,[-50.,0.35],at)
```

[5]:
```
f2 = figure(2, figsize=(12,18))
subplot2grid((4,2),(0,0),colspan=2)
plot(at,res[:,0])
subplot2grid((4,2),(1,0),colspan=2)
plot(at,vectorize(getCurr)(at))
plot([500.,9000.],[I0,I1],'o')

subplot2grid((4,2),(2,0))
plot(at[:200000],res[:200000,0])
subplot2grid((4,2),(2,1))
plot(at[-200000:],res[-200000:,0])
subplot2grid((4,2),(3,0))
plot(v0c[:,0],v0c[:,1])
plot(v0cI0[:,0],v0cI0[:,1])
plot(vm,vectorize(L2)(vm,v4,0.,v5,1.,0.,0.),"k-")
plot(res[:200000,0],res[:200000,1])
subplot2grid((4,2),(3,1))
plot(v0c[:,0],v0c[:,1])
plot(v0cI1[:,0],v0cI1[:,1])
plot(vm,vectorize(L2)(vm,v4,0.,v5,1.,0.,0.),"k-")
plot(res[-200000:,0],res[-200000:,1])
f2.savefig("pls-t2-ramp-saddlenode-depblk.svg")
f2.savefig("pls-t2-ramp-saddlenode-depblk.png")
show()
```

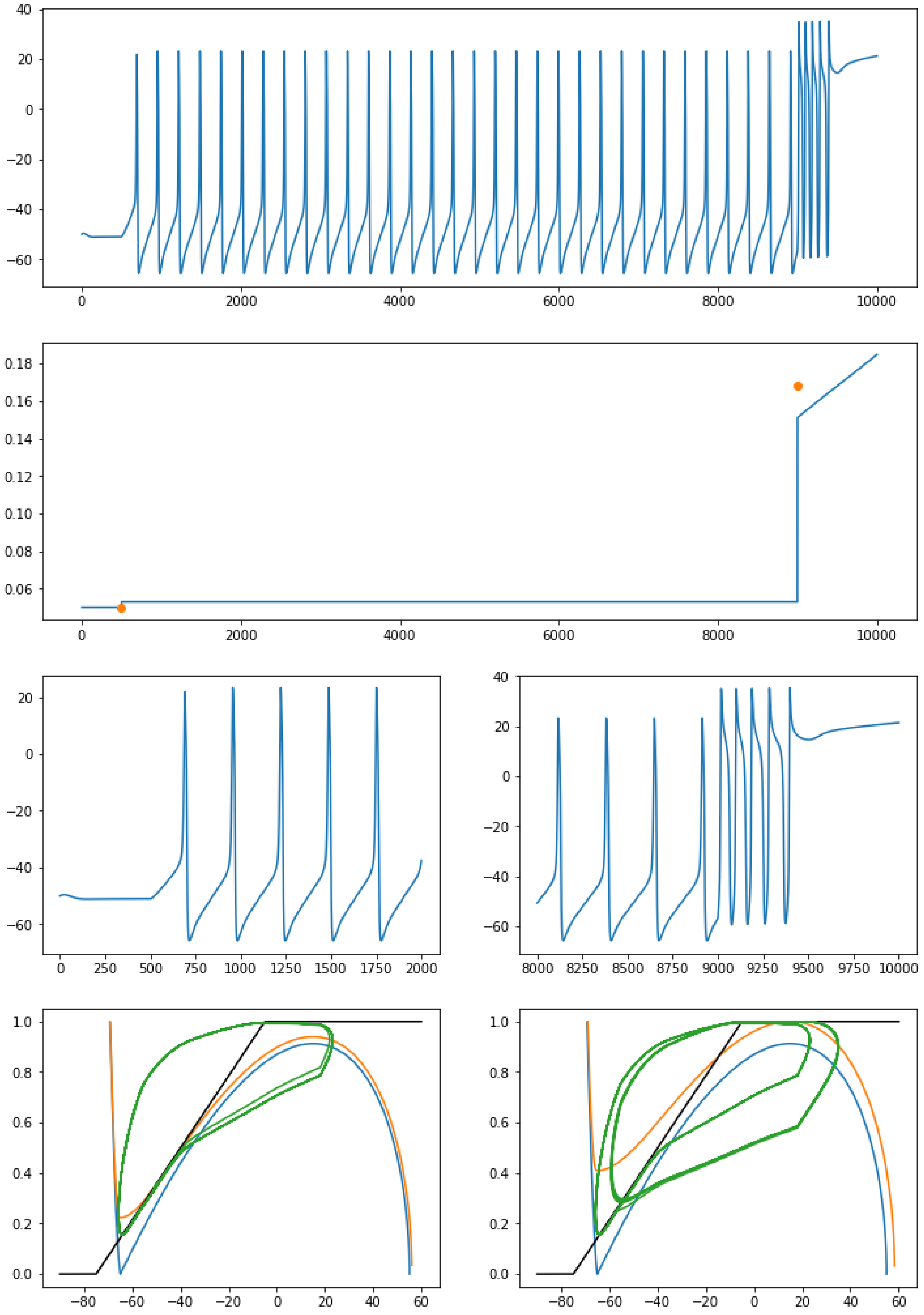

40
20
0
−20
−40
−60
0
2000
4000
6000
8000
10000
0.18
0.16
0.14
0.12
0.10
0.08
0.06
0
250
500
750
1000
1250
1500
1750
2000
8000
8250
8500
8750
9000
9250
9500
9750
10000
1.0
0.8
0.6
0.4
0.2
0.0
−80
−60
−40
−20
0
20
40
60

1.2 bistability at bifurcation

[6]:
```
def t2rhs(Y,t):
    v,w = Y
    return[
        (P32(v,v0,v2)*L1(v,v0,a0,a1,0.)+I0*1.055-w**wp)/L1(v,v3,r0,r1,0.),
        (L2(v,v4,0.,v5,1.,0.,0.)-w)/S2(v,v6,v7,s0,s1,s2)
    ]
at = arange(0,10000,0.01)
res0 = odeint(t2rhs,[-47.,0.4]     ,at[:at.shape[0]//2])
res1 = odeint(t2rhs,[res0[-1,0],0.],at[at.shape[0]//2:])

f3 = figure(3, figsize=(12,18))
subplot2grid((4,2),(0,0),colspan=2)
plot(at,hstack((res0[:,0],res1[:,0])))
show()
```

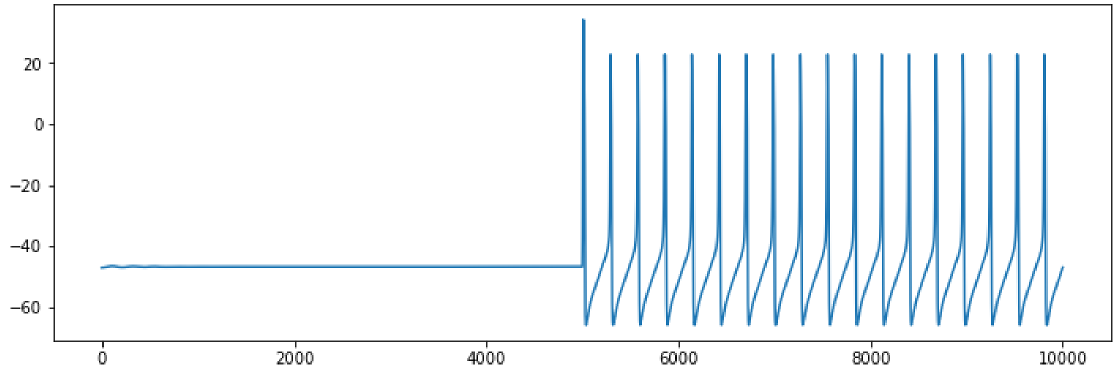

1.3 with linear adaptaion

$$
\begin{aligned}
L_1(v,v_3,r_0,r_1,0) \quad \dot{v} &= P_{32}(v,v_0,v_2)L_1(v,v_0,a_0,a_1,0)+I-w^2-z \\
S_2(v,v_6,,v_7,s_0,s_1,s_2) \quad \dot{w} &= L_2(v,v_4,0,v_5,1,0,0)-w \\
q_0 \quad \dot{z} &= L_2(v,v_8,z_0,v_9,z_1,0,0)-z
\end{aligned}
$$

[7]:
```
#--- z_\infty
v8,v9     = -35.,10.
z0,z1     = 0., 2.
#--- \tua_z
q0        = 500
```

[8]:
```
def t1rhs(Y,t):
    v,w,z = Y
    return[
        (P32(v,v0,v2)*L1(v,v0,a0,a1,0.)+I1-w**wp-z)/L1(v,v3,r0,r1,0.),
```

```
        (L2(v,v4,0.,v5,1.,0.,0.)-w)/S2(v,v6,v7,s0,s1,s2),
        (L2(v,v8,z0,v9,z1,0.,0.)-z)/q0
    ]

at = arange(0,10000,0.01)
res = odeint(t1rhs,[v0,0,0.],at)
```

[9]:

```
v0c   = safesqrt(vm, vectorize(P32)(vm,v0,v2)*vectorize(L1)(vm,v0,a0,a1,0.)+0.␣
 ↪,1)
v0cI0 = safesqrt(vm, vectorize(P32)(vm,v0,v2)*vectorize(L1)(vm,v0,a0,a1,0.)+I1␣
 ↪,1)
v0cI1 = safesqrt(vm, vectorize(P32)(vm,v0,v2)*vectorize(L1)(vm,v0,a0,a1,0.
 ↪)+I1-amax(res[:,2]) ,1)



f2 = figure(2, figsize=(12,18))
subplot2grid((3,2),(0,0),colspan=2)
plot(at,res[:,0])
subplot2grid((3,2),(1,0))
plot(at,res[:,1])
subplot2grid((3,2),(1,1))
plot(at,res[:,2])
subplot2grid((3,2),(2,0))
plot(v0c[:,0],v0c[:,1])
plot(v0cI0[:,0],v0cI0[:,1])
plot(vm,vectorize(L2)(vm,v4,0.,v5,1.,0.,0.),"k-")
plot(res[:200000,0],res[:200000,1])
subplot2grid((3,2),(2,1))
plot(v0c[:,0],v0c[:,1])
plot(v0cI1[:,0],v0cI1[:,1])
plot(vm,vectorize(L2)(vm,v4,0.,v5,1.,0.,0.),"k-")
plot(res[-200000:,0],res[-200000:,1])
show()
```

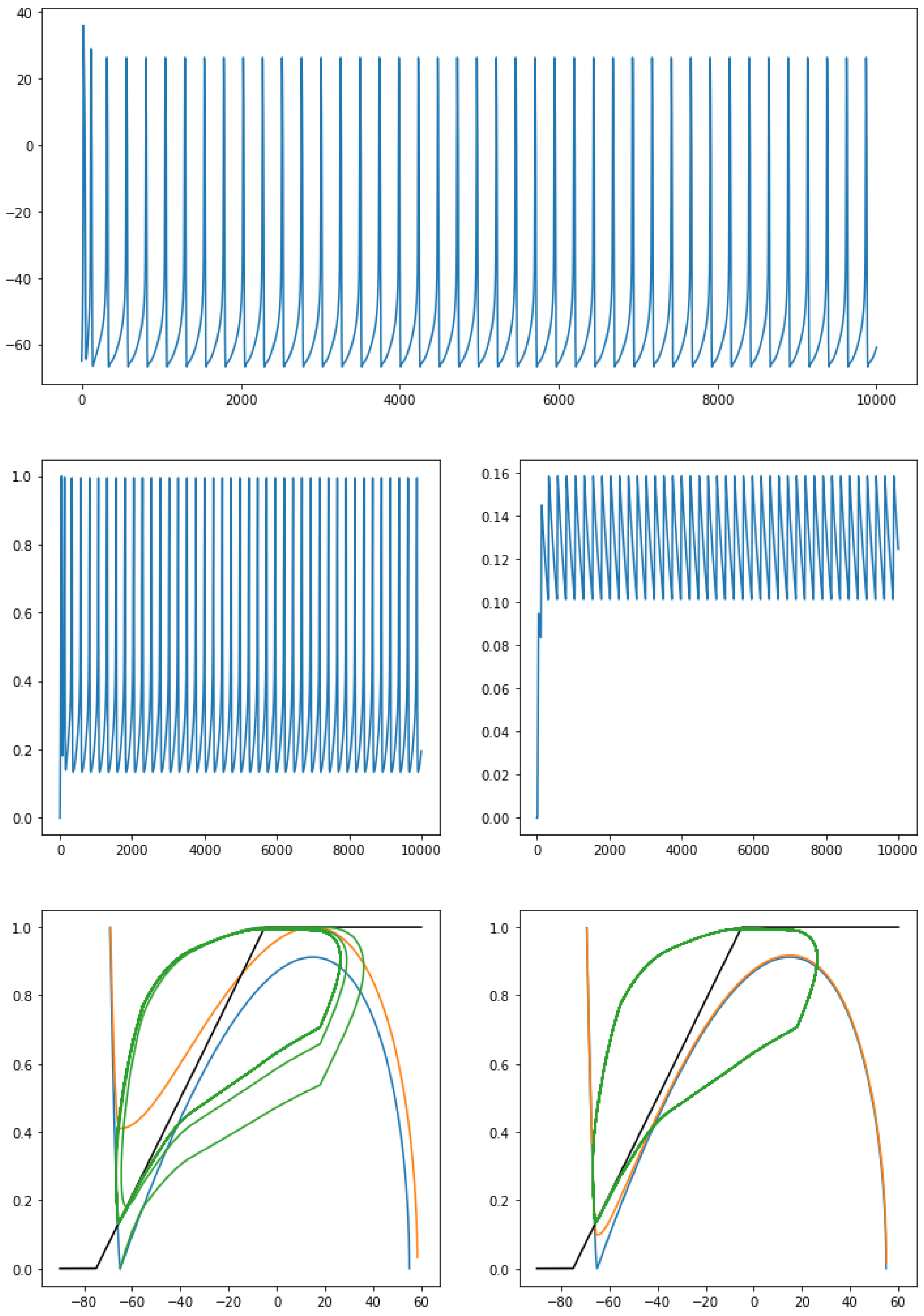
40
20
0
−20
−40
−60
0
2000
4000
6000
8000
10000
1.0
0.8
0.6
0.4
0.2
0.0
0
2000
4000
6000
8000
10000
0.16
0.14
0.12
0.10
0.08
0.06
0.04
0.02
0.00
0
2000
4000
6000
8000
10000
1.0
0.8
0.6
0.4
0.2
0.0
−80
−60
−40
−20
0
20
40
60
1.0
0.8
0.6
0.4
0.2
0.0
−80
−60
−40
−20
0
20
40
60

1.4 with non-linear adaptaion

$$
\begin{aligned}
L_1(v,v_3,r_0,r_1,0) \quad \dot{v} &= P_{32}(v,v_0+z,v_2)L_1(v,v_0+z,a_0,a_1,0)+I-w^2 \\
S_2(v,v_6,,v_7,s_0,s_1,s_2) \quad \dot{w} &= L_2(v,v_4,0,v_5,1,0,0)-w \\
q_0 \quad \dot{z} &= L_2(v,v_8,z_0,v_9,z_1,0,0)-z
\end{aligned}
$$

[10]:
```
#--- z_\infty
v8,v9     = -35.,10.
Delta     = 150
z0,z1     = 0,Delta
#--- \tua_z
q0        = 500
```

[11]:
```
def t1rhs(Y,t):
    v,w,z = Y
    return[
        (P32(v,v0+z,v2)*L1(v,v0+z,a0,a1,0.)+I1-w**wp)/L1(v,v3,r0,r1,0.),
        (L2(v,v4,0.,v5,1.,0.,0.)-w)/S2(v,v6,v7,s0,s1,s2),
        (L2(v,v8,z0,v9,z1,0.,0.)-z)/q0
    ]

at = arange(0,10000,0.01)
res = odeint(t1rhs,[v0,0,0.],at)
```

[12]:
```
v0c   = safesqrt(vm, vectorize(P32)(vm,v0,v2)*vectorize(L1)(vm,v0,a0,a1,0.)+0.␣
↪,1)
v0cI0 = safesqrt(vm, vectorize(P32)(vm,v0,v2)*vectorize(L1)(vm,v0,a0,a1,0.)+I1␣
↪,1)
zm    = amax(res[:,2])
v0cI1 = safesqrt(vm, vectorize(P32)(vm,v0+zm,v2)*vectorize(L1)(vm,v0+zm,a0,a1,0.
↪)+I1 ,1)


f2 = figure(2, figsize=(12,18))
subplot2grid((3,2),(0,0),colspan=2)
plot(at,res[:,0])
subplot2grid((3,2),(1,0))
plot(at,res[:,1])
subplot2grid((3,2),(1,1))
plot(at,res[:,2])
subplot2grid((3,2),(2,0))
plot(v0c[:,0],v0c[:,1])
plot(v0cI0[:,0],v0cI0[:,1])
plot(vm,vectorize(L2)(vm,v4,0.,v5,1.,0.,0.),"k-")
plot(res[:200000,0],res[:200000,1])
subplot2grid((3,2),(2,1))
plot(v0c[:,0],v0c[:,1])
```

```
plot(v0cI1[:,0],v0cI1[:,1])
plot(vm,vectorize(L2)(vm,v4,0.,v5,1.,0.,0.),"k-")
plot(res[-20000:,0],res[-20000:,1])

show()
```

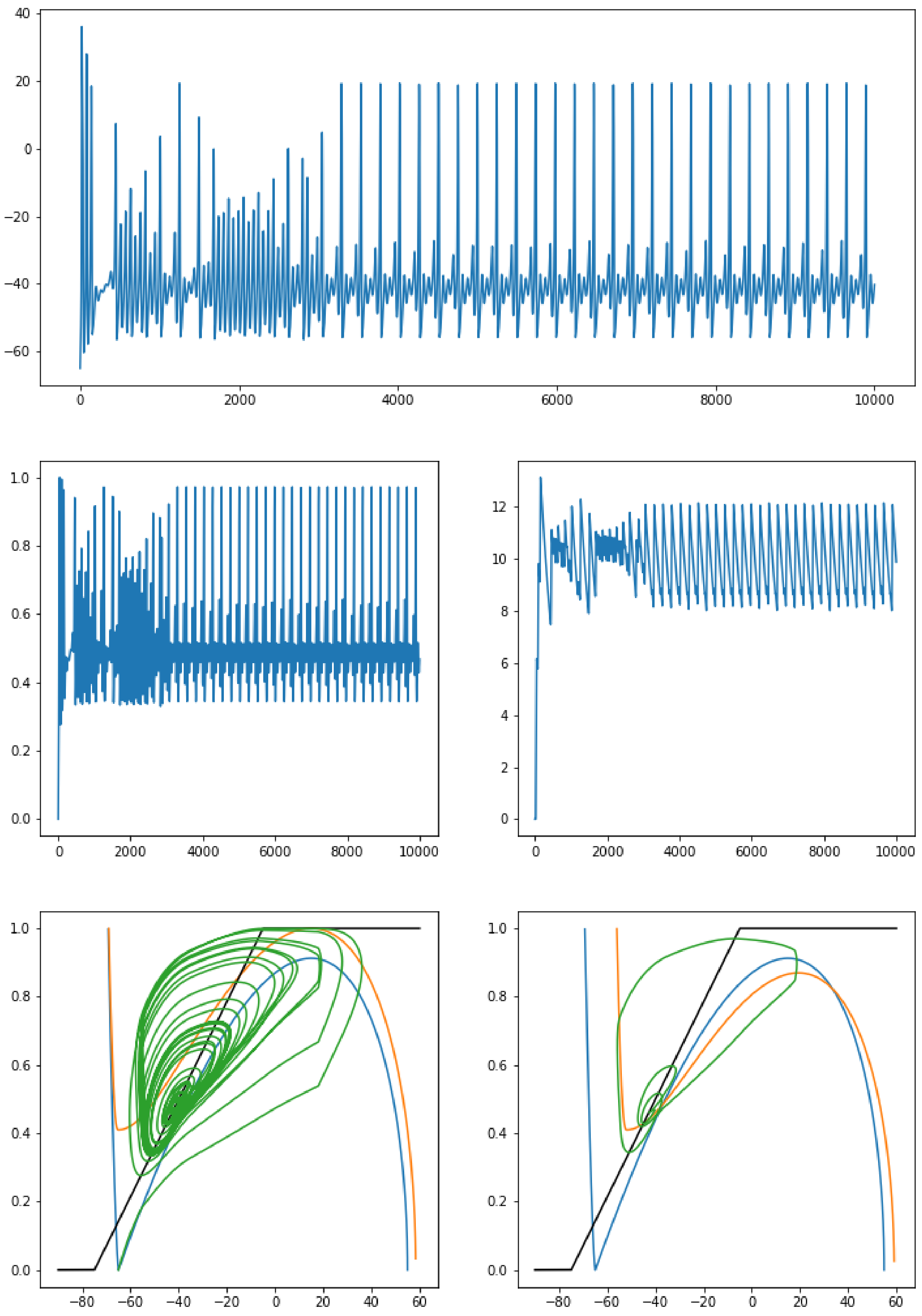

40
20
0
−20
−40
−60
0
2000
4000
6000
8000
10000
1.0
0.8
0.6
0.4
0.2
0.0
0
2000
4000
6000
8000
10000
12
10
8
6
4
2
0
0
2000
4000
6000
8000
10000
1.0
0.8
0.6
0.4
0.2
0.0
−80
−60
−40
−20
0
20
40
60
1.0
0.8
0.6
0.4
0.2
0.0
−80
−60
−40
−20
0
20
40
60

1.5 non biological adaptation

$$\begin{aligned} L_1(v,v_3,r_0,r_1,0) \quad \dot{v} &= P_{32}(v,v_0,v_2)L_1(v,v_0,a_0,a_1,0) + I - w^2 \\ S_2(v,v_6,,v_7,s_0,s_1,s_2) \quad \dot{w} &= L_2(v,v_4-z,0,v_5-z,1,0,0) - w \\ q_0 \quad \dot{z} &= L_2(w,w_0,z_0,w_1,z_1,0,0) - z \end{aligned}$$

[13]:
```
#--- z_\infty
w0,w1      = 0.5,0.8
Delta      = 50
z0,z1      = 0,Delta
#--- \tua_z
q0         = 500

Iapp       = I1*.7
def t1rhs(Y,t):
    v,w,z = Y
    return[
        (P32(v,v0,v2)*L1(v,v0,a0,a1,0.)+Iapp-w**wp)/L1(v,v3,r0,r1,0.),
        (L2(v,v4-z,0.,v5-z,1.,0.,0.)-w)/S2(v,v6,v7,s0,s1,s2),
        (L2(w,w0,z0,w1,z1,0.,0.)-z)/q0
    ]

at = arange(0,10000,0.01)
res = odeint(t1rhs,[v0,0,0.],at)

v0c   = safesqrt(vm, vectorize(P32)(vm,v0,v2)*vectorize(L1)(vm,v0,a0,a1,0.)+0.␣
 ↪,1)
v0cI0 = safesqrt(vm, vectorize(P32)(vm,v0,v2)*vectorize(L1)(vm,v0,a0,a1,0.
 ↪)+Iapp ,1)
zm    = amax(res[:,2])
wm    = linspace(0.,1., 101)
zy    = linspace(z0,z1, 101)
vmean = mean(res[:,0])



f2 = figure(2, figsize=(12,18))
subplot2grid((3,2),(0,0),colspan=2)
plot(at,res[:,0])
subplot2grid((3,2),(1,0))
plot(at,res[:,1])
subplot2grid((3,2),(1,1))
plot(at,res[:,2])
subplot2grid((3,2),(2,0))
plot(v0cI0[:,0],v0cI0[:,1])
plot(vm,vectorize(L2)(vm,v4   ,0.,v5   ,1.,0.,0.),"k-")
plot(vm,vectorize(L2)(vm,v4-zm,0.,v5-zm,1.,0.,0.),"k--")
```

```
plot(res[:,0],res[:,1])
subplot2grid((3,2),(2,1))
plot(wm,vectorize(L2)(wm,w0,z0,w1,z1,0.,0.))
plot(vectorize(L2)(v0   ,v4-zy,0.,v5-zy,1.,0.,0.),zy,'k-')
plot(vectorize(L2)(vmean,v4-zy,0.,v5-zy,1.,0.,0.),zy,'k--')
plot(res[:,1],res[:,2])

show()
```

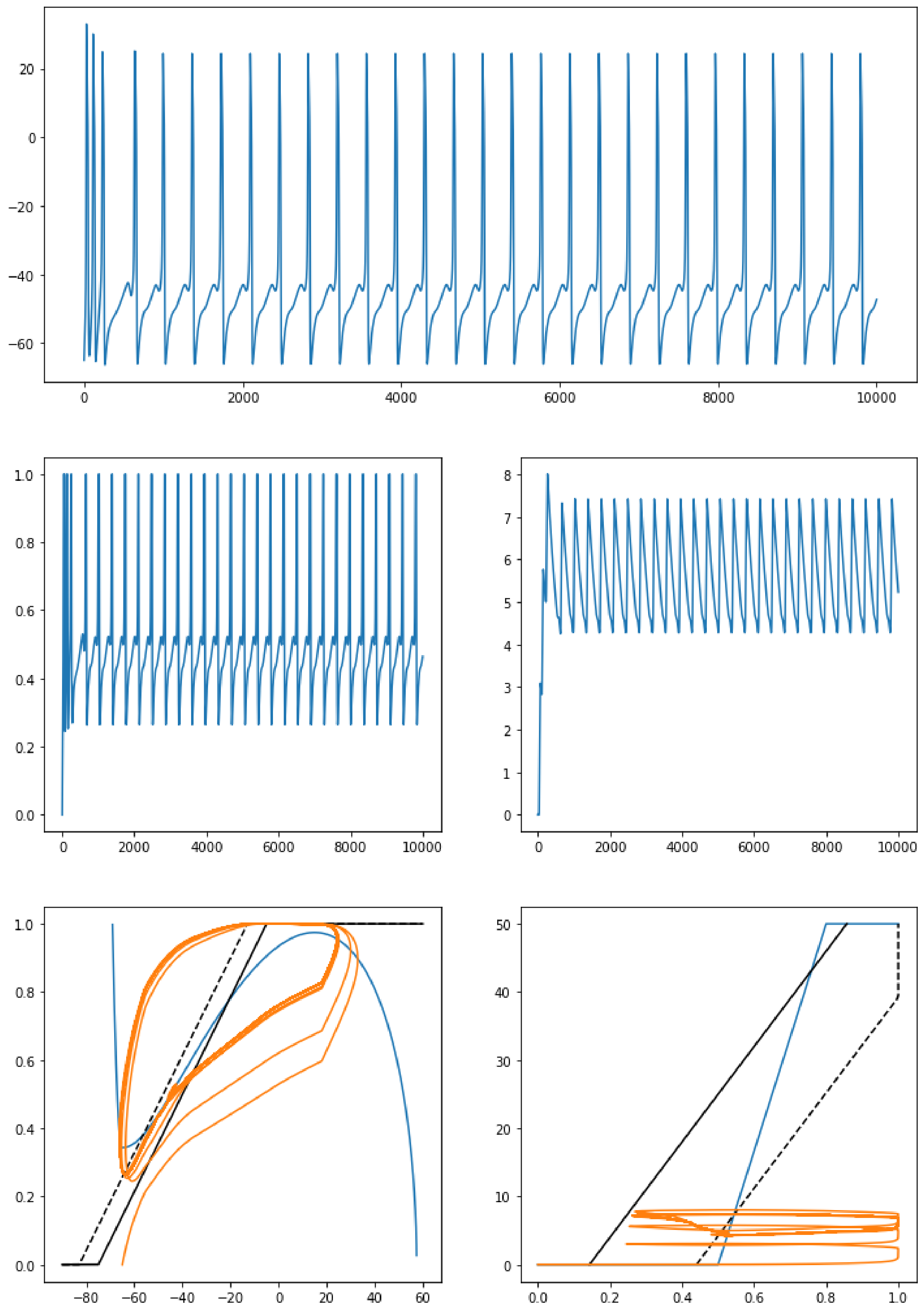

20
0
−20
−40
−60
0
2000
4000
6000
8000
10000
1.0
0.8
0.6
0.4
0.2
0.0
0
2000
4000
6000
8000
10000
8
7
6
5
4
3
2
1
0
0
2000
4000
6000
8000
10000
1.0
0.8
0.6
0.4
0.2
0.0
−80
−60
−40
−20
0
20
40
60
50
40
30
20
10
0
0.0
0.2
0.4
0.6
0.8
1.0
