# Supplementary material for: Polynomial, piecewise-Linear, Step (PLS): A Simple, Scalable, and Efficient Framework for Modeling Neurons
Source: Front Neuroinform. 2021 May 6;15:642933. doi: 10.3389/fninf.2021.642933 (PMC8134741; doi:10.3389/fninf.2021.642933)
Supplement: Supplementary Data Sheet 3 — Listing of Python 3 Jupyther-notebook page with all five model tests in section 5. [file Data_Sheet_3.PDF]

Plateau-Potentials-4models

March 1, 2021

[3]:

```
%matplotlib inline
from numpy import *
from matplotlib.pyplot import *
from brian2 import *

Mg2           = 1.
tAMPA         = 2.*ms
tNMDA1,tNMDA2 = 1.*ms, 200.*ms
Es            = 0.
rates = ones(30)*0.9
rates[3:4] = 13.
R = TimedArray(rates*Hz, dt=1.*second)
P = PoissonGroup(30, rates="R(t)")



#Izhikevich model
izh_gsyn = 0.007
izh_gAMPA, izh_gNMDA  = izh_gsyn, izh_gsyn*4
izh_a,izh_b,izh_c, izh_d = 0.02, 0.2, -65., 8. #Regular firing
equ_izh="""
dv/dt = ( (0.04*v**2+5.*v+140)-u+izh_gNMDA*(b-a)*(Es-v)/(1 + Mg2 * exp(-0.062*v/
 ↪3.57) ) + izh_gAMPA*c*(Es-v) )/ms : 1 (unless refractory)
du/dt = izh_a*(izh_b*v-u)/ms : 1 (unless refractory)
da/dt = -a/tNMDA1   : 1
db/dt = -b/tNMDA2   : 1
dc/dt = -c/tAMPA    : 1
"""
izh = NeuronGroup(1,equ_izh,threshold="v>30", reset="v=izh_c; u=izh_d",␣
 ↪refractory=0.1*ms, method="euler")
izh.v = -60.
p2izh = Synapses(P, izh, on_pre='a_post+=1 ; b_post+=1; c_post += 1')
p2izh.connect()
izh_vmon = StateMonitor(izh, 'v', record=True)

#aduptive eLIF
aelf_gsyn = 1.5
```

```
aelf_gAMPA, aelf_gNMDA  = aelf_gsyn*nS, aelf_gsyn*4*nS
aelf_C,aelf_gL,aelf_EL,aelf_VT = 281*pF,30*nS, -70.6*mV, -50.4*mV
aelf_DeltaT = 2*mV
aelf_Vcut = aelf_VT + 5 * aelf_DeltaT
aelf_tauw, aelf_a, aelf_b, aelf_Vr = 144*ms, 4*nS,         0.0805*nA, -70.6*mV␣
↪# Regular spiking (as in the paper)
#aelf_tauw, aelf_a, aelf_b, aelf_Vr =  20*ms, 4*nS,         0.5*nA,      VT+5*mV␣
↪# Bursting
#aelf_tauw, aelf_a, aelf_b, aelf_Vr = 144*ms, 2*C/(144*ms),0.*nA,       -70.6*mV␣
↪# Fast spiking

aelf_equ = """
dv/dt=(aelf_gL*(aelf_EL-v)+aelf_gL*aelf_DeltaT*exp((v-aelf_VT)/
↪aelf_DeltaT)-w+aelf_gNMDA*(b-a)*(Es*mV-v)/(1 + Mg2 * exp(-0.062*(v/mV)/3.57)␣
↪) + aelf_gAMPA*c*(Es*mV-v))/aelf_C : volt
dw/dt=(aelf_a*(v-aelf_EL)-w)/aelf_tauw : amp
da/dt = -a/tNMDA1   : 1
db/dt = -b/tNMDA2   : 1
dc/dt = -c/tAMPA    : 1
"""
aelf = NeuronGroup(1, aelf_equ, threshold='v > aelf_Vcut', reset="v = aelf_Vr;␣
↪w += aelf_b", method='euler')
aelf.v = aelf_EL
aelf.w = 0*amp
p2aelf = Synapses(P, aelf, on_pre='a_post+=1 ; b_post+=1; c_post += 1')
p2aelf.connect()
aelf_vmon = StateMonitor(aelf, 'v', record=True)
aelf_smon = SpikeMonitor(aelf)

#eLIF with threshold adaptation
#from links below with some adaptations
#https://journals.plos.org/ploscompbiol/article?id=10.1371/journal.pcbi.1003560
#https://journals.plos.org/ploscompbiol/article?id=10.1371/journal.pcbi.
↪1004984#sec025
telf_gsyn = 3.9
telf_gL = 10.
telf_gAMPA, telf_gNMDA  = telf_gsyn/telf_gL, telf_gsyn*4/telf_gL
telf_tauv,telf_EL,telf_DeltaT = 10*ms,-70.*mV,1.*mV
telf_a,telf_ka,telf_kl    = 0.3,7.*mV,8.75*mV
telf_VT,telf_VI,telf_tauw = -61.*mV, -59.6*mV, 26.*ms
telf_Vr = -70.*mV

telf_equ = """
dv/dt=((telf_EL-v)+telf_DeltaT*exp((v-w)/telf_DeltaT)+telf_gNMDA/
↪telf_gL*(b-a)*(Es*mV-v)/(1 + Mg2 * exp(-0.062*(v/mV)/3.57) ) + telf_gAMPA/
↪telf_gL*c*(Es*mV-v))/telf_tauv : volt
```

```
dw/dt=(telf_a*(v-telf_VI)+telf_VT+telf_ka*log(1.+exp((v-telf_VI)/telf_kl))-w)/
 ↪telf_tauw : volt
da/dt = -a/tNMDA1   : 1
db/dt = -b/tNMDA2   : 1
dc/dt = -c/tAMPA    : 1
"""
telf = NeuronGroup(1, telf_equ, threshold='v > w+3*mV', reset="v = telf_Vr",␣
 ↪refractory=0.5*ms, method='euler')
telf.v = telf_EL
telf.w = telf_VT
p2telf = Synapses(P, telf, on_pre='a_post+=1 ; b_post+=1; c_post += 1')
p2telf.connect()
telf_vmon = StateMonitor(telf, 'v', record=True)
telf_smon = SpikeMonitor(telf)

#PLS Type 1 neuron without adaptation
plsdb_gsyn = 0.77e-4
plsdb_gAMPA, plsdb_gNMDA  = plsdb_gsyn, plsdb_gsyn*4
#--- v_\infty
plsdb_v0,plsdb_v1,plsdb_v2 = -65.,-45., 55.
plsdb_a0,plsdb_a1          = 2.7e-6,1e-4
plsdb_vmin,plsdb_vmax      = -55.45176241886681, 18.78509575220014
#--- \tau_v
plsdb_v3                   = -35.
plsdb_r0,plsdb_r1          = 0.04, 0.004
#--- w_\infty
plsdb_v4,plsdb_v5          = -40.,-5
plsdb_v45                  = plsdb_v5-plsdb_v4
#--- \tau_w
plsdb_v6,plsdb_v7          = plsdb_vmin,plsdb_vmax
plsdb_s0,plsdb_s1,plsdb_s2 = 5.,7.6,1.8
plsdb_s01,plsdb_s12        = plsdb_s1-plsdb_s0,plsdb_s2-plsdb_s1

plsdb_equ="""
dv/
 ↪dt=((v-plsdb_v0)*(v-plsdb_v1)*(plsdb_v2-v)*(plsdb_a0+plsdb_a1*int(v<plsdb_v0))-w**2+plsdb_g[illegible]
 ↪(1 + Mg2 * exp(-0.062*v/3.57) ) + plsdb_gAMPA*c*(Es-v))/
 ↪(plsdb_r0+plsdb_r1*(plsdb_v3-v)*int(v<plsdb_v3))/ms : 1
dw/dt=(1.-(plsdb_v5-v)/plsdb_v45*int(v<plsdb_v5)+((plsdb_v5-v)/
 ↪plsdb_v45-1)*int(v<plsdb_v4) - w )/
 ↪(plsdb_s0+plsdb_s01*int(v>plsdb_v6)+plsdb_s12*int(v>plsdb_v7))/ms : 1
da/dt = -a/tNMDA1   : 1
db/dt = -b/tNMDA2   : 1
dc/dt = -c/tAMPA    : 1
"""
```

```
plsdb = NeuronGroup(1, plsdb_equ, threshold='v > 0',  refractory='v<-10',␣
 ↪method='euler')
plsdb.v = plsdb_v0
plsdb.w = 0.
p2plsdb = Synapses(P, plsdb, on_pre='a_post+=1 ; b_post+=1; c_post += 1')
p2plsdb.connect()
plsdb_vmon = StateMonitor(plsdb, 'v', record=True)

#PLS Type 2 neuron without adaptation
plsop_gsyn = 1.27e-4
plsop_gAMPA, plsop_gNMDA  = plsop_gsyn, plsop_gsyn*4
plsop_v0,plsop_v2          = -65., 55.
plsop_a0,plsop_a1          = 3.5e-6,1e-4
#--- \tau_v
plsop_v3                   = -35.
plsop_r0,plsop_r1          = 0.04, 0.004
#--- n_\infty
plsop_v4,plsop_v5          = -75.,-7.
plsop_v45                  = plsop_v5-plsop_v4
#--- \tau_n
plsop_v6,plsop_v7          = -55.5,18.
plsop_s0,plsop_s1,plsop_s2 = 5.,7.6,1.8
plsop_s01,plsop_s12        = plsop_s1-plsop_s0,plsop_s2-plsop_s1
plsop_equ="""
dv/
 ↪dt=((v-plsop_v0)*(v-plsop_v0)*(plsop_v2-v)*(plsop_a0+plsop_a1*int(v<plsop_v0))-w**2+plsop_g
 ↪(1 + Mg2 * exp(-0.062*v/3.57) ) + plsop_gAMPA*c*(Es-v))/
 ↪(plsop_r0+plsop_r1*(plsop_v3-v)*int(v<plsop_v3))/ms : 1
dw/dt=(1.-(plsop_v5-v)/plsop_v45*int(v<plsop_v5)+((plsop_v5-v)/
 ↪plsop_v45-1)*int(v<plsop_v4) - w )/
 ↪(plsop_s0+plsop_s01*int(v>plsop_v6)+plsop_s12*int(v>plsop_v7))/ms : 1
da/dt = -a/tNMDA1   : 1
db/dt = -b/tNMDA2   : 1
dc/dt = -c/tAMPA    : 1
"""

plsop = NeuronGroup(1, plsop_equ, threshold='v > 0',  refractory='v<-10',␣
 ↪method='euler')
plsop.v = plsop_v0
plsop.w = 0.
p2plsop = Synapses(P, plsop, on_pre='a_post+=1 ; b_post+=1; c_post += 1')
p2plsop.connect()
plsop_vmon = StateMonitor(plsop, ['v','w','a','b','c'], record=True)

run(20000*ms)

f1 = figure(1,figsize=(24,24))
```

```
ax = subplot(511)
plot(izh_vmon.t/ms,izh_vmon.v[0])
subplot(512,sharex=ax,sharey=ax)
aelf_v = array(aelf_vmon.v[0]/mV)
for ts in aelf_smon.t:
    aelf_v[argmin((aelf_vmon.t-ts)**2)]=30.
plot(aelf_vmon.t/ms,aelf_v)
subplot(513,sharex=ax,sharey=ax)
telf_v = array(telf_vmon.v[0]/mV)
for ts in telf_smon.t:
    telf_v[argmin((telf_vmon.t-ts)**2)]=30.
plot(telf_vmon.t/ms,telf_v)
subplot(514,sharex=ax,sharey=ax)
plot(plsdb_vmon.t/ms,plsdb_vmon.v[0])
subplot(515,sharex=ax,sharey=ax)
plot(plsop_vmon.t/ms,plsop_vmon.v[0])
f1.savefig("Plateau-Potentials-4m-overall.png",dpi='figure')
f1.savefig("Plateau-Potentials-4m-overall.svg",dpi='figure')
show()
```

```
WARNING    "rates" is an internal variable of group "poissongroup_1", but also
exists in the run namespace with the value array([0.9, 0.9, 0.9, …, 0.9, 0.9,
0.9]). The internal variable will be used.
[brian2.groups.group.Group.resolve.resolution_conflict]
```

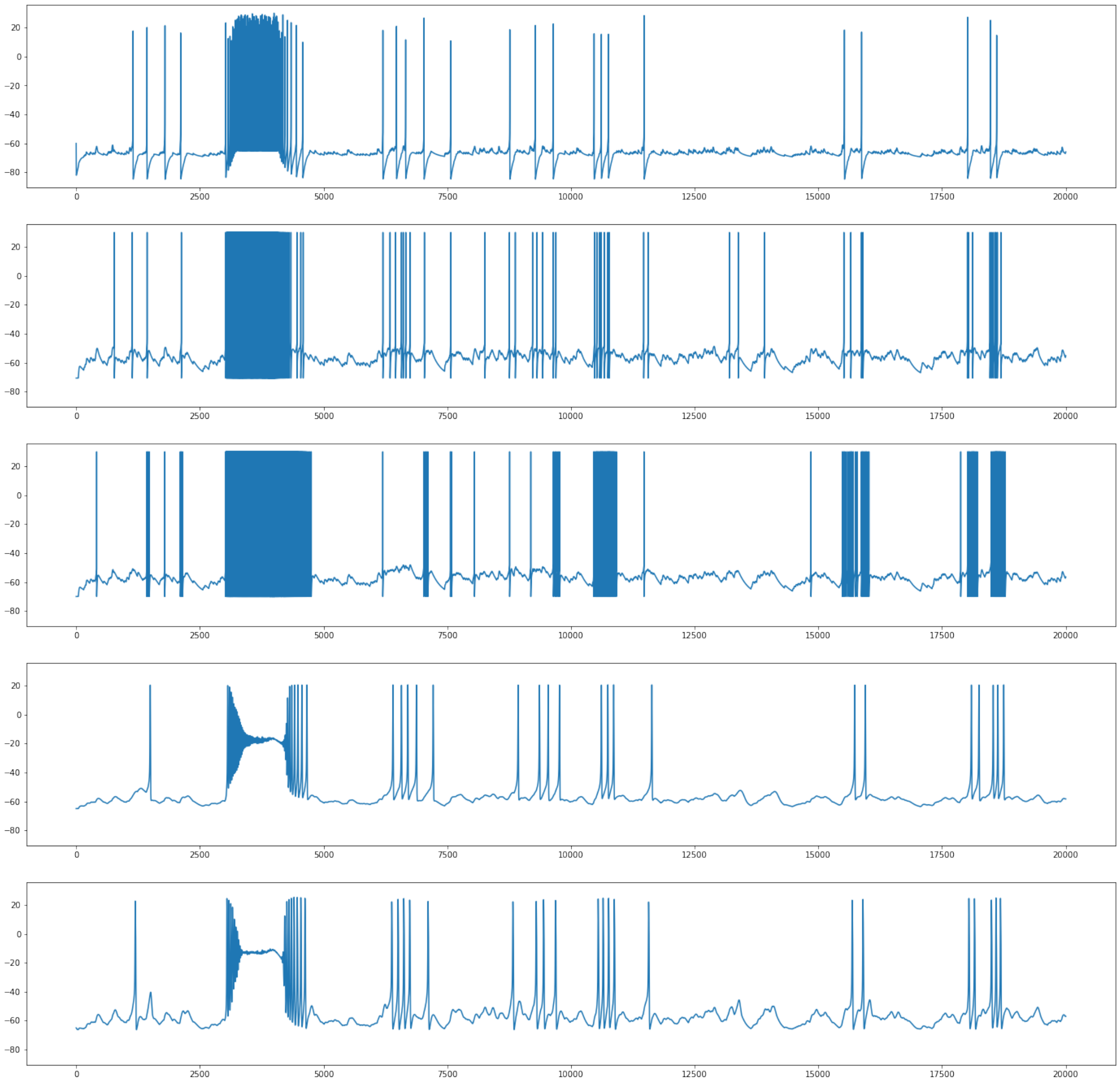

[8]:
```
f2 = figure(2,figsize=(12,24))
#LEFT
ax1 = subplot(5,2,1)
mask=where((izh_vmon.t>2500*ms)*(izh_vmon.t<3500*ms))[0]
plot(izh_vmon.t[mask]/ms,izh_vmon.v[0][mask])
subplot(5,2,3,sharex=ax1,sharey=ax1)
mask=where((aelf_vmon.t>2500*ms)*(aelf_vmon.t<3500*ms))[0]
plot(aelf_vmon.t[mask]/ms,aelf_v[mask])
subplot(5,2,5,sharex=ax1,sharey=ax1)
mask=where((telf_vmon.t>2500*ms)*(telf_vmon.t<3500*ms))[0]
plot(telf_vmon.t[mask]/ms,telf_v[mask])
subplot(5,2,7,sharex=ax1,sharey=ax1)
mask=where((plsdb_vmon.t>2500*ms)*(plsdb_vmon.t<3500*ms))[0]
plot(plsdb_vmon.t[mask]/ms,plsdb_vmon.v[0][mask])
```

```
subplot(5,2,9,sharex=ax1,sharey=ax1)
mask=where((plsop_vmon.t>2500*ms)*(plsop_vmon.t<3500*ms))[0]
plot(plsop_vmon.t[mask]/ms,plsop_vmon.v[0][mask])


#RIGHT
ax2 = subplot(5,2,2)
mask=where((izh_vmon.t>4000*ms)*(izh_vmon.t<5000*ms))[0]
plot(izh_vmon.t[mask]/ms,izh_vmon.v[0][mask])
subplot(5,2,4,sharex=ax2,sharey=ax2)
mask=where((aelf_vmon.t>4000*ms)*(aelf_vmon.t<5000*ms))[0]
plot(aelf_vmon.t[mask]/ms,aelf_v[mask])
subplot(5,2,6,sharex=ax2,sharey=ax2)
mask=where((telf_vmon.t>4000*ms)*(telf_vmon.t<5000*ms))[0]
plot(telf_vmon.t[mask]/ms,telf_v[mask])
subplot(5,2,8,sharex=ax2,sharey=ax2)
mask=where((plsdb_vmon.t>4000*ms)*(plsdb_vmon.t<5000*ms))[0]
plot(plsdb_vmon.t[mask]/ms,plsdb_vmon.v[0][mask])
subplot(5,2,10,sharex=ax2,sharey=ax2)
mask=where((plsop_vmon.t>4000*ms)*(plsop_vmon.t<5000*ms))[0]
plot(plsop_vmon.t[mask]/ms,plsop_vmon.v[0][mask])

f2.savefig("Plateau-Potentials-4m-BurstOnOff.png",dpi='figure')
f2.savefig("Plateau-Potentials-4m-BurstOnOff.svg",dpi='figure')

show()
```

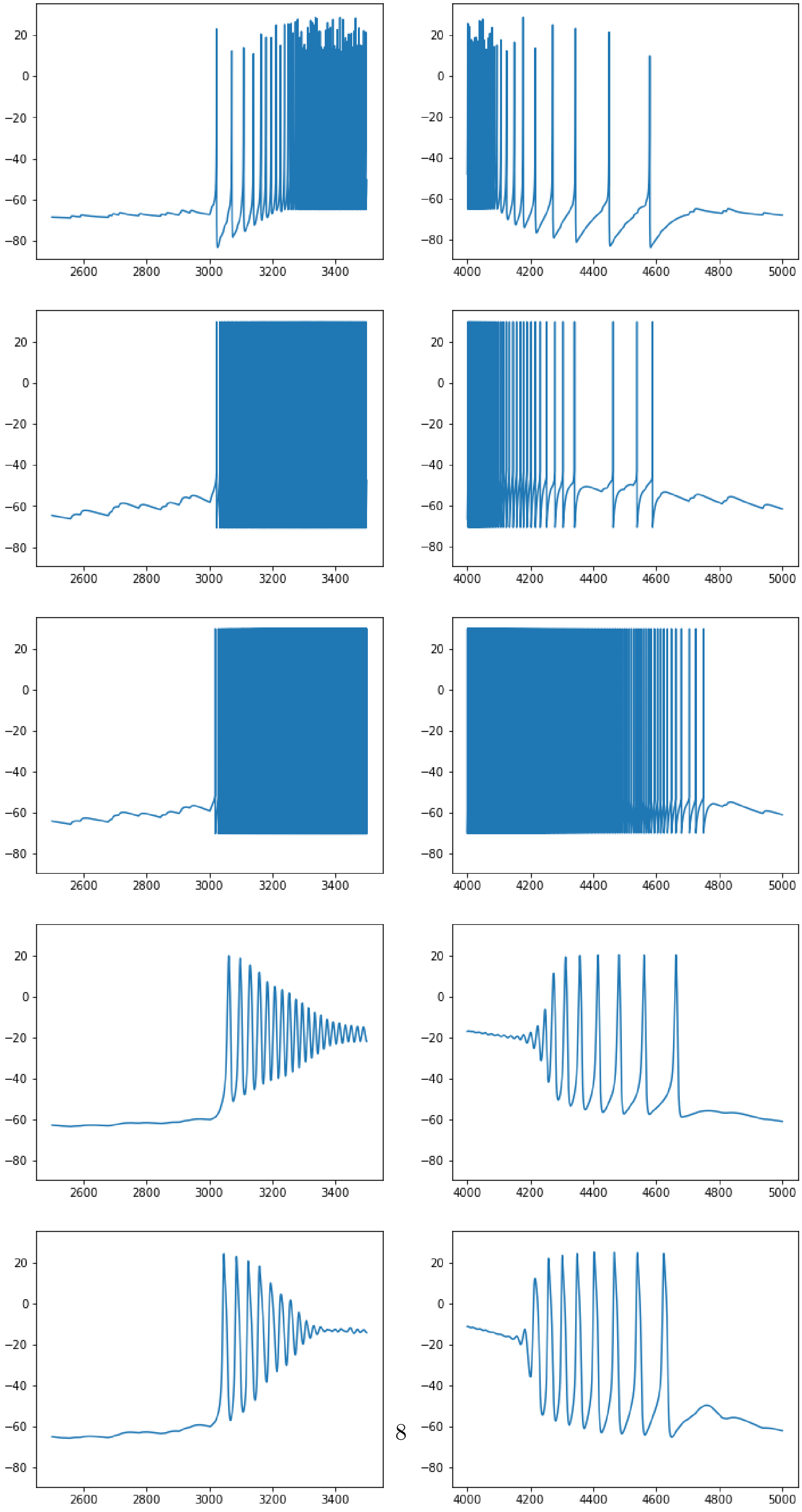

20
0
−20
−40
−60
−80
2600
2800
3000
3200
3400
4000
4200
4400
4600
4800
5000

[9]:

```
f3 = figure(3,figsize=(24,24))

ax1 = subplot2grid((5,3),(0,0),colspan=2)
plot(izh_vmon.t/ms,izh_vmon.v[0],"k-")
ax2 = subplot2grid((5,3),(0,2))
mask=where((izh_vmon.t>2500*ms)*(izh_vmon.t<5000*ms))[0]
plot(izh_vmon.t[mask]/ms,izh_vmon.v[0][mask],"k-")
subplot2grid((5,3),(1,0),colspan=2,sharex=ax1,sharey=ax1)
plot(aelf_vmon.t/ms,aelf_v,"k-")
subplot2grid((5,3),(1,2),sharex=ax2,sharey=ax2)
mask=where((aelf_vmon.t>2500*ms)*(aelf_vmon.t<5000*ms))[0]
plot(aelf_vmon.t[mask]/ms,aelf_v[mask],"k-")
subplot2grid((5,3),(2,0),colspan=2,sharex=ax1,sharey=ax1)
plot(telf_vmon.t/ms,telf_v,"k-")
subplot2grid((5,3),(2,2),sharex=ax2,sharey=ax2)
mask=where((telf_vmon.t>2500*ms)*(telf_vmon.t<5000*ms))[0]
plot(telf_vmon.t[mask]/ms,telf_v[mask],"k-")
subplot2grid((5,3),(3,0),colspan=2,sharex=ax1,sharey=ax1)
plot(plsdb_vmon.t/ms,plsdb_vmon.v[0],"k-")
subplot2grid((5,3),(3,2),sharex=ax2,sharey=ax2)
mask=where((plsdb_vmon.t>2500*ms)*(plsdb_vmon.t<5000*ms))[0]
plot(plsdb_vmon.t[mask]/ms,plsdb_vmon.v[0][mask],"k-")
subplot2grid((5,3),(4,0),colspan=2,sharex=ax1,sharey=ax1)
plot(plsop_vmon.t/ms,plsop_vmon.v[0],"k-")
subplot2grid((5,3),(4,2),sharex=ax2,sharey=ax2)
mask=where((plsop_vmon.t>2500*ms)*(plsop_vmon.t<5000*ms))[0]
plot(plsop_vmon.t[mask]/ms,plsop_vmon.v[0][mask],"k-")

f3.savefig("Plateau-Potentials-4m-All-and-Burst.png",dpi='figure')
f3.savefig("Plateau-Potentials-4m-All-and-Burst.svg",dpi='figure')

show()
```

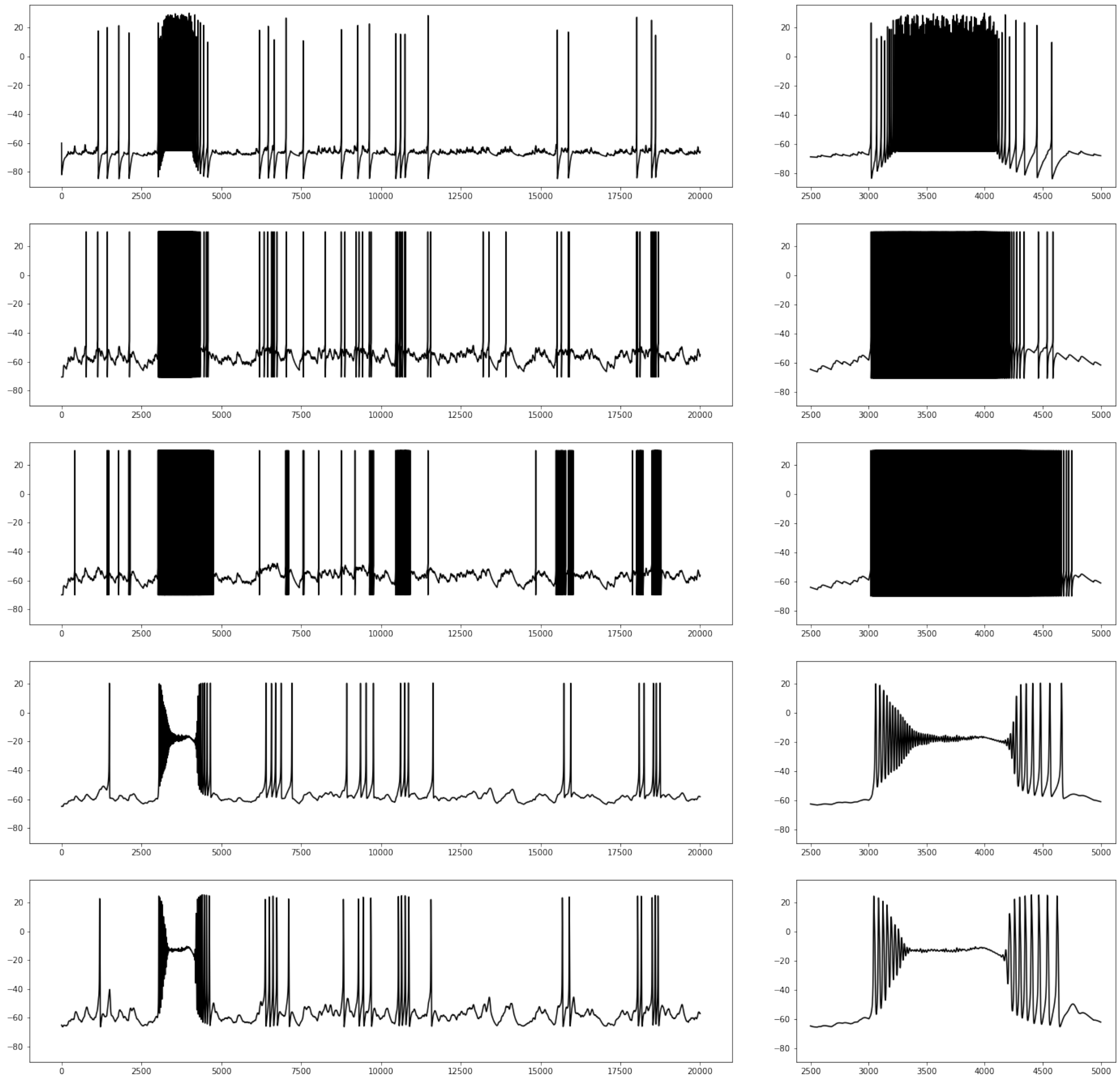
20
0
−20
−40
−60
−80
0
2500
5000
7500
10000
12500
15000
17500
20000
2500
3000
3500
4000
4500
5000
