# Supplementary material for: Polynomial, piecewise-Linear, Step (PLS): A Simple, Scalable, and Efficient Framework for Modeling Neurons
Source: Front Neuroinform. 2021 May 6;15:642933. doi: 10.3389/fninf.2021.642933 (PMC8134741; doi:10.3389/fninf.2021.642933)
Supplement: Supplementary file 8 [file Data_Sheet_4.ZIP › PLS-framework/readme.html]

*These Jupyter notebooks associated with the paper:*

### Polynomial, piecewise-Linear, Step (PLS): a simple, scalable and efficient framework for modeling neurons\*\*

#### by Ruben A. Tikidji-Hamburyan and Matthew T. Colonnese

---

### Requirements

To use this scripts you need **Python 3.8** and python’s libraries:

- numpy
- scipy
- matplotlib and LaTeX for correct graphical interface
- jupyter
- cython
- brian2

### Files in this directory

| File | Description |
| --- | --- |
| WangBuzsaki-model-reduction.ipynb | Wang&Buzsaki model reduction (section 2) |
| WangBuzsaki-model-reduction-euler.ipynb | the same reduction with Euler method |
| WangBuzsaki-model-reduction-c-code/wbcl.c | c-code for *fully computed* benchmark |
| WangBuzsaki-model-reduction-c-code/wbpol.c | c-code for *polynomial* benchmark |
| WangBuzsaki-model-reduction-c-code/wbpwl.c | c-code for *piecewise-Linear* benchmark |
| WangBuzsaki-model-reduction-c-code/wbmix.c | c-code for *PL2D* benchmark |
| WangBuzsaki-model-reduction-c-code/\*.cu | the same benchmarks for CUDA GPU test |
| WangBuzsaki-model-reduction-c-code/makefile | make file for building CPU (`make all`) and GPU (`make gpu`) tests |
| WangBuzsaki-model-reduction-c-code/test-cpu.sh | compile and run test of CPU |
| WangBuzsaki-model-reduction-c-code/test-gpu.sh | compile and run test of GPU |
| PLS-Integrator.ipynb | Phenomenological model: Type 1 (section 4) |
| PLS-Resonator.ipynb | Phenomenological model: Type 2 (section 4) |
| Plateau-Potentials-4models.ipynb | Plateau Potentials test (section 5) |
| pls.py | Helper: PLS Family in Python |
| pls.xpp | Helper: PLS Family in XPP |
| pls.h | Helper: PLS Family in C/C++/NEURON(mod)/etc |
| mrth.py | General Helper |
